# Large-scale in silico mutagenesis experiments reveal optimization of genetic code and codon usage for protein mutational robustness

**DOI:** 10.1186/s12915-020-00870-9

**Published:** 2020-10-20

**Authors:** Martin Schwersensky, Marianne Rooman, Fabrizio Pucci

**Affiliations:** 1 Computational Biology and Bioinformatics, Université Libre de Bruxelles, CP 165/61, Roosevelt Ave. 50, Brussels, 1050 Belgium; 2 Interuniversity Institute of Bioinformatics in Brussels, Boulevard du Triomphe, Brussels, 1050 Belgium

**Keywords:** Protein evolution, Stability prediction, Mutational robustness, Translation accuracy, Genetic code, Codon usage, Codon usage bias

## Abstract

**Background:**

How, and the extent to which, evolution acts on DNA and protein sequences to ensure mutational robustness and evolvability is a long-standing open question in the field of molecular evolution. We addressed this issue through the first structurome-scale computational investigation, in which we estimated the change in folding free energy upon all possible single-site mutations introduced in more than 20,000 protein structures, as well as through available experimental stability and fitness data.

**Results:**

At the amino acid level, we found the protein surface to be more robust against random mutations than the core, this difference being stronger for small proteins. The destabilizing and neutral mutations are more numerous in the core and on the surface, respectively, whereas the stabilizing mutations are about 4% in both regions. At the genetic code level, we observed smallest destabilization for mutations that are due to substitutions of base III in the codon, followed by base I, bases I+III, base II, and other multiple base substitutions. This ranking highly anticorrelates with the codon-anticodon mispairing frequency in the translation process. This suggests that the standard genetic code is optimized to limit the impact of random mutations, but even more so to limit translation errors. At the codon level, both the codon usage and the usage bias appear to optimize mutational robustness and translation accuracy, especially for surface residues.

**Conclusion:**

Our results highlight the non-universality of mutational robustness and its multiscale dependence on protein features, the structure of the genetic code, and the codon usage. Our analyses and approach are strongly supported by available experimental mutagenesis data.

## Background

Amino acid mutations can have different impacts on protein stability and fitness. Most are substantially destabilizing and potentially cause the partial or complete loss of structure and function. However, the large majority of amino acid mutations that become fixed upon evolution, called amino acid substitutions in the evolutionary field, are regarded as neutral with respect to protein fitness [1]. Note that substitutions can also lead to the emergence of new functions, although with a very low frequency of about 10^−9^ per site, thus driving functional evolution [2].

Two concepts play a central role in these matters. The first is mutational robustness, which refers to the capacity to tolerate mutations without changing the molecular and/or organism’s phenotype. It is anticorrelated with the evolutionary or selection pressure, which means that residues that are more robust to mutations are less constrained by selection. The second concept is evolvability, which is defined as the capacity of proteins to acquire new functions, hence allowing them to adapt to modifications in the environment.

Despite recent advances, the role of the evolutionary mechanisms in the complex interplay between the optimization of these two fundamental but sometimes conflicting characteristics is still a major issue in molecular evolution and protein biophysics [3–10]. A wide variety of disciplines, from synthetic biology to protein design, would definitely benefit from a better understanding of these mechanisms and from the ability of accurately predicting the future evolutionary processes from the analysis of the past [11].

Mutational robustness and evolvability can be viewed as two sides of the same coin, which drive evolution in an entangled way. On the one hand, physical principles are expected to favor structured proteins with a high degree of stability, while on the other hand, the selection for function imposes opposite constraints in targeted regions, such as the presence of amino acids carrying specific chemical moieties or a required degree of structural flexibility. Once the functional criteria are satisfied, mutational robustness ensures better tolerance of random mutations in non-functional regions and thus confers an evolutionary advantage [12, 13]. Note, however, that too high tolerance to mutations can also prevent necessary adaptation to environmental changes [14].

Results obtained from experimental analyses and theoretical models of population genetics suggest that mutational robustness is favored or disfavored, and impedes or facilitates adaptative evolution, according to the polymorphicity and size of the population, the mutation rate, and the fitness landscape [5, 14–16].

To further shed light on these challenging issues, we performed an extensive in silico mutagenesis study, in which we computed the change in protein thermodynamic stability caused by all single point mutations inserted in the structurome, defined as the ensemble of all protein structures available in the Protein Data Bank [17]. This is the first systematic and comprehensive investigation at such a large scale, using bioinformatics tools of which the validity has largely been demonstrated in different contexts. On top of that, we also analyzed available experimental data on stability changes and fitness, which brings experimental support to our analyses.

The first issue that we studied in detail is how the mutational robustness is influenced by some protein characteristics. A series of papers have studied the impact of residue- to organism-level properties, such as residue hydrophobicity, protein size, organism type, and growth temperature [18–21]. We focused here on protein length and residue solvent accessibility, as their influence has to be taken care of when examining nucleobase-level impacts.

A second question concerns the relation between the mutational robustness and the standard genetic code (SGC). It has been shown that this code has evolved to minimize the costs of amino acid replacements. Indeed, from the observation of the SGC table (Additional file 1: Figure S1), we immediately see that amino acids that share similar biophysical characteristics tend to be encoded in codons that differ by only a single base. However, a long and controversial debate regards the level of optimality that the SGC has reached [22–28].

On the basis of the nucleobase sequence of the whole structurome, we also investigated the relation between the mutational robustness, the codon choice, and the codon usage bias. Indeed, the degeneracy of the genetic code introduces some variability into protein encoding in nucleobase sequences, which opens alternative pathways in the evolutionary landscape that are likely to allow, e.g., the minimization of translational errors and an effective increase of protein mutational robustness [29–31]. Codons are selected for other reasons too, such as the matching of tRNA abundance and the mRNA stability for improved translation efficiency [31–34].

## Results and discussion

The central question addressed here concerns the protein robustness against mutations, its dependence on various parameters at the codon, residue, and protein levels, and its link with evolutionary rates.

With this objective in mind, we estimated with the PoPMuSiC ^sym^ algorithm [35, 36] the change in folding free energy (*ΔΔG*) for all single-site mutations in the non-redundant set $\mathcal {D}$ of protein X-ray structures representing the protein structurome, as described in the “Methods” section. In parallel, we considered the smaller ensembles of experimentally measured *ΔΔG* values and fitness scores. These three sets of mutations, that we call $\mathcal {M}_{\text {PoP}}, \mathcal {M}_{\text {Exp}}$, and $\mathcal {M}_{\text {Fit}}$ contain about 1.0×10^8^,2.6×10^3^, and 1.4×10^4^ mutations, respectively.

Most of the natural amino acid mutations are the result of a single base substitution (SBS) in the codon, as the evolutionary probability to have simultaneously two or three base substitutions is small. However, we would like to point out that only a subset of all possible amino acid mutations can be obtained through SBSs. We call such amino acid mutations *μ*SBS and limit ourselves to this subset unless otherwise stated. The amino acid mutations that result from multiple base substitutions (MBS) are called *μ*MBSs.

### Relative solvent accessibility

**Fig. 1.**
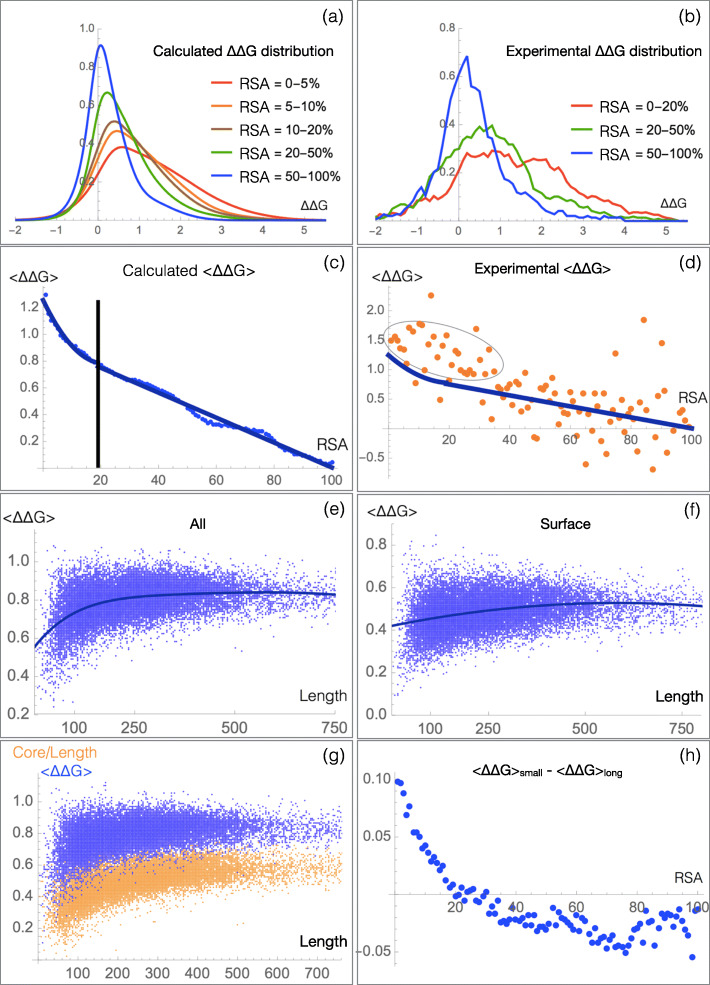
Influence of the protein length and of the mutated residues’ RSA (in %) on the mutational robustness, evaluated from the *ΔΔG* values (in kcal/mol) of *μ*SBSs from the sets ${\mathcal {M}_{\text {PoP}}}$ (a,c,e-h) and ${\mathcal {M}_{\text {Exp}}}$ (b,d). **a**, **b** *ΔΔG* distribution for different RSA ranges. **c**, **d** Mean *ΔΔG* per RSA bin as a function of the RSA; the chosen bin width is equal to 1%. **e** Mean *ΔΔG* per protein as a function of the protein length for all residues and **f** for surface residues (RSA >20%). **g** Mean *ΔΔG* per protein as a function of protein length (blue points) and protein core to length ratio, defined as the number of residues in the core over the number of residues in the protein (orange points). **h** Difference between the mean *ΔΔG* per RSA bin of long proteins (*L*>200 residues) and short proteins (*L*≤200 residues) as a function of RSA

We started by analyzing the effect of the relative solvent accessibility (RSA) of the mutated residues on the mutational robustness. This effect is clearly visible in Fig. 1a, b: the *ΔΔG* distribution of *μ*SBSs is much more spread out and shifted toward destabilizing mutations for core residues than for surface residues in agreement with earlier findings [37, 38]. Random mutations are thus on average much more destabilizing when introduced in the core, where close packing and specific interactions tend to impede changes in residue size and physicochemical properties. In contrast, surface residues are more robust to mutations than core residues, in the sense that they have a smaller impact on the thermodynamic stability.

It has to be stressed that these results are almost identical whether using the set of computed or experimental *ΔΔG*s from $\mathcal {M}_{\text {PoP}}$ and $\mathcal {M}_{\text {Exp}}$ (*cf.* Fig. 1a, b). This supports the validity and accuracy of PoPMuSiC ^sym^’s *ΔΔG* predictions.

We also found that the relationship between RSA and *ΔΔG* values is linear above an RSA threshold of about 20% and non-linear below this threshold, where the curve is well fitted by a second-degree polynomial function (Fig. 1c-d). Again, the same trend is observed for the computed and experimental mutations of $\mathcal {M}_{\text {PoP}}$ and $\mathcal {M}_{\text {Exp}}$, with an even stronger deviation from linearity at small RSA values for the latter; note that the number of mutations in $\mathcal {M}_{\text {Exp}}$ is low, which explains the noisy behavior.

### Protein length

The effects of residue RSA and protein size on the mutational robustness are entangled. Indeed, mutations of residues located in the core, which have a low RSA, have on average a larger impact on stability than surface mutations, which have a large RSA. As a consequence, proteins of different sizes, which have different core to surface ratios, appear to have different tolerances to mutations [39].

The dependence of mutational robustness on protein length *L* is shown in Fig. 1e. On average, shorter proteins that have a smaller core to surface ratio are more robust than longer proteins for which this ratio is larger. Above about 400 residues, the robustness remains roughly constant. Such large proteins are usually multi-domain proteins, which implies that the core to surface ratio does not increase any more.

To gain insights into this effect, we computed the *L*-dependence separately for core and surface residues. We found that shorter proteins tend to have a less robust core and a more robust surface than larger proteins, as shown in Fig. 1e, f and Addional file 1: Figure S2, in agreement with some previous studies [40–43].

The former observation can be attributed to the larger compactness and hydrophobicity of the core of short proteins, which is therefore less able to accommodate mutations. We indeed checked that the core becomes less and less hydrophobic as the protein size increases (Additional file 1: Figure S3). In fact, the increase in core to surface ratio is compensated up to a certain level by variations in the amino acid composition. However, this compensation is far from perfect, and the core of small proteins is definitely more hydrophobic than that of large proteins [40]. For example, the hydrophobic residues (Val, Ile, Leu, Phe) represent about 45% of buried residues in proteins of *L*≤200, 41% for medium-size proteins (200<*L*≤400) residues, and only about 37% in larger proteins (400<*L*).

Several hypotheses can be formulated to explain the lower hydrophobicity of the core of large proteins. It can simply be due to “incomplete” evolution, in the sense that their hydrophobic character would still be increasing throughout natural evolution [40]. Alternatively, it can be argued that core contacts have to be stronger in small than in large proteins and thus on the average more hydrophobic, given that short proteins have a smaller number of native contacts per residue that can be used to compensate the loss of conformational entropy upon folding [41, 42].

The second observation, i.e., the higher mutational robustness of the surface of small proteins compared to the surface of longer proteins, can be explained by the larger fraction of functional residues. These residues are known to be poorly optimized for protein stability, but well optimized for function, including protein-protein and protein-ligand interactions, conformational changes, and catalytic activity [21, 44, 45]. Therefore, their substitutions are likely to be stabilizing, which confers a higher mutational robustness to the surface of small proteins. Another explanation could be related to different levels of negative design pressure, which tends to destabilize and thus avoid misfolded structures, in contrast to positive design that strengthens native interactions [41–43].

Finally note that the mean *ΔΔG* per protein, 〈*ΔΔG*〉, is, on average, proportional to the fraction of residues in the core, as seen in Fig. 1g. This follows from the facts that core mutations have much larger *ΔΔG* values on average than surface mutations and that their effect dominates when computing the mean.

### Evolutionary rate

We compared the mutational robustness analyzed in the previous sections with the evolutionary rate, defined as the ratio of nonsynonymous to synonymous base substitutions, which has been estimated in a series of papers on the basis of sequence evolution models [46–50]. These two quantities are expected to be related given that stability is known to be one of the major factors contributing to the evolutionary pressure [51–53].

The dependence of the evolutionary rate on RSA was investigated in [46, 47]. A larger rate was found for surface than for core residues. This is in agreement with our findings of a larger mutational robustness. In brief, surface residues, whose mutations have on average smaller effects on protein stability, evolve faster than buried residues.

However, while the relationship between RSA and evolutionary rate appears to be linear [46, 47], the relationship between RSA and mutational robustness is shown to be linear only for RSA values larger than 20% (Fig. 1c, d). This suggests that mutational robustness and evolutionary rate are linearly correlated solely for surface residues. The relation becomes non-linear for core residues, with the robustness decreasing more than the rate.

Note that our results indicate a monotonic relation between mutational robustness and evolutionary rate, whereas other predictions based on stability against misfolding rather suggest a non-monotonic relation [54].

Finally, the RSA-evolutionary rate regression line has been suggested to have a larger slope for large than for small proteins [46, 47]. More precisely, surface residues from large proteins seem to evolve faster than those from small proteins, whereas almost no difference is observed for core residues. These results appear a priori to be in contradiction with ours. Indeed, we found that small proteins have a more robust surface and a less robust core than large proteins on the basis of both predicted and experimental *ΔΔG* values (Fig. 1h and S4).

There is in fact no contradiction. Rather, we run here up against the limits of the correlation between evolutionary rate and mutational robustness: small proteins have a more robust and slower evolving surface than large proteins and a less robust and equally evolving core. The interpretation of this difference lies in the fact that a significant proportion of surface residues are functional, especially in small proteins. These functional residues increase the robustness by lowering the 〈*ΔΔG*〉 as they are not optimized for stability [21, 44, 45] and decrease the evolutionary rate as many mutations render the protein non-functional. Note that this counterintuitive result is due to our definition of robustness in terms of protein stability rather than fitness (see the next section).

### Experimental fitness

To compare the computed mutational robustness with experimental fitness measures, we subdivided the mutations into stabilizing, neutral, and destabilizing, using the free energy thresholds: *ΔΔG*<−0.5 kcal/mol, − 0.5 kcal/mol ≤*ΔΔG*≤0.5 kcal/mol, and 0.5 kcal/mol <*ΔΔG*, respectively.

**Fig. 2.**
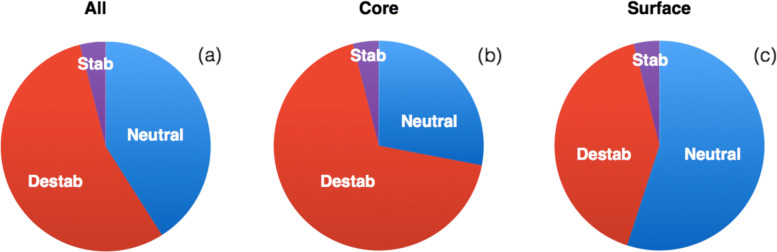
Fraction of stabilizing *μ*SBSs (*ΔΔG*<−0.5 kcal/mol), neutral *μ*SBSs (− 0.5 ≤*ΔΔG*≤0.5 kcal/mol) and destabilizing *μ*SBSs (*ΔΔG*>0.5 kcal/mol) in $\mathcal {M}_{\text {PoP}}$ for **a** all residues, **b** core residues (RSA ≤20*%*), and **c** surface residues (RSA >20*%*)

With these definitions, the fractions of destabilizing, neutral, and stabilizing *μ*SBSs from $\mathcal {M}_{\text {PoP}}$ are (68%, 28%, 4%) in the core, (41%, 55%, 4%) on the surface and (55%, 41%, 4%) overall (Fig. 2 and Additional file 1: Table S1 for more detailed RSA dependence).

Note that, in the set of experimental *μ*SBSs of $\mathcal {M}_{\text {Exp}}$, the fraction of stabilizing mutations is slightly higher (about 10 to 12%, according to whether they are introduced in the core or at the surface). This is not surprising as these mutations are non-random; they are engineered and biased toward stabilizing mutations.

The fraction of stabilizing mutations obtained via a single base substitution is thus constant and equal to 4% of the total number of mutations both in the core and on the surface. In contrast, destabilizing *μ*SBSs dominate in the core and neutral *μ*SBSs dominate on the surface. Of course, the precise fractions of stabilizing, neutral, and destabilizing mutations depend on the somewhat arbitrary threshold energy values of − 0.5 and + 0.5 kcal/mol.

We compared these results with experimentally characterized fitness values of random mutations, taken from three different studies and grouped in $\mathcal {M}_{\text {Fit}}$ (Table 1). Note that the concept of fitness is not precisely defined and depends on the experimental setup used to characterize it. Stability is for sure a major factor [51], but fitness contains also other factors, related to, e.g., protein expression, solubility, and function.

**Table 1:** Comparison between mutational robustness and fitness: computed fraction of destabilizing, neutral, and stabilizing *μ*SBSs from $\mathcal {M}_{\text {PoP}}$ and experimentally characterized fraction of deleterious, neutral, and advantageous mutations. The fitness thresholds for defining the mutation phenotypes are chosen by the authors for mutations in [55]; for the other sets of experimental mutations: deleterious if the fitness is lower than the mean of loss-of-function and wild-type scores, neutral if the fitness is between that threshold and 1.25 times the wild-type score, and advantageous otherwise

Mutation set	Destabilizing	Neutral	Stabilizing	Reference
$\mathcal {M}_{\text {PoP}}$	55%	41%	4%	This paper
Mutations in	Deleterious	Neutral	Advantageous	Reference
AraC/D/E	53%	43%	4%	[55]
UBE2I/SUMO1/CALM1/TPK1	51%	44%	5%	[56]
TEM-1	37%	59%	4%	[57]

The first study involves about 150 mutations inserted in three proteins (the transcription factor AraC, the enzyme AraD and the transporter AraE) [55]. Among these mutations, the number of deleterious, neutral and advantageous mutations were found to be equal to 53%, 43%, and 4% on average, with some differences between the three tested proteins. These values are close to the fractions of destabilizing, neutral, and stabilizing mutations that we predicted for the full structurome.

A second experimental investigation used deep mutagenesis scanning to investigate about 13,000 mutations in four proteins (SUMO E2 conjugase, a small ubiquitin-like modifier, thiamin pyrophosphokinase and calmodulin). The percentage of deleterious (51%), neutral (44%), and advantageous (5%) mutations [56] also fits very well with our predictions.

The third series of experimental results concerns the mutational landscape of TEM-1 *β*-lactamase, with about 800 mutants [57]. In this case, a bigger fraction of neutral than of destabilizing mutations was found. This could suggest that the activity of this enzyme is particularly well optimized as already observed in [57].

We would like to underline the good agreement between our predictions and these mutagenesis data, which contain about 10 times more mutations than the training set of our predictor (basically $\mathcal {M}_{\text {Exp}}$) and has negligible overlap with it. This proofs the good generalization properties of our predictor (as also discussed in the “Methods” section) which successfully generalizes the statistics-based rules derived from the small training dataset $\mathcal {M}_{\text {Exp}}$ to large independent datasets.

### Similarity matrices

Similarity matrices, such as the series of BLOSUM matrices [58], are commonly used in sequence alignment methods to account for the similarity between the 20 amino acids and the ease with which they are mutated into each other. They are derived from multiple sequence alignments of homologous proteins and thus reflect both the physicochemical similarity of the substituted amino acids, the evolutionary mechanisms acting on protein sequences, and the structure of the genetic code.

We expected a certain correlation between BLOSUM scores and mutational robustness, as they share stability as one of their main ingredients [18, 59] and more specifically, hydrophobicity [60]. Moreover, BLOSUM and fitness scores have been shown to correlate well in the mutational landscape of TEM-1 *β*-lactamase [57].

We focused here on mutational robustness rather than fitness, expanded the analysis to the ensemble of all *μ*SBSs in the structurome set $\mathcal {M}_{\text {PoP}}$, and computed the *ΔΔG* distribution as a function of the BLOSUM scores. We considered for that purpose the commonly used BLOSUM62 matrix.

We clearly observe a strong correlation between the mean *ΔΔG* and the BLOSUM62 score, with a linear correlation coefficient as high as *r* = −0.97. As shown in Table 2, the substitutions that are the most likely to occur during natural evolution are mostly neutral for stability and only a small fraction is destabilizing. The picture is completely reversed for the substitutions that are less likely to occur. Indeed, these substitutions impact on average quite strongly on protein stability, while only a very small fraction is neutral. Interestingly, the fraction of stabilizing mutations is almost constant, between 3 and 5%, except for mutations between very similar amino acids where it drops to 1%.

**Table 2:** Mean *ΔΔG* (in kcal/mol) of all *μ*SBSs in $\mathcal {M}_{\text {PoP}}$ as a function of the BLOSUM62 class. Positive BLOSUM scores indicate more likely amino acid substitutions and negative scores, less likely substitutions. The fraction of stabilizing (Stab), neutral (Neut), and destabilizing (Dest) substitutions in each class is also reported

BLOSUM	〈*ΔΔG*〉	Stab (%)	Neut (%)	Dest (%)
− 4	1.58	4	18	78
− 3	1.15	5	29	66
− 2	1.11	4	27	69
− 1	0.83	4	34	62
0	0.56	5	46	49
1	0.33	3	65	32
2	0.28	3	71	26
3	0.25	1	78	21

**Fig. 3.**
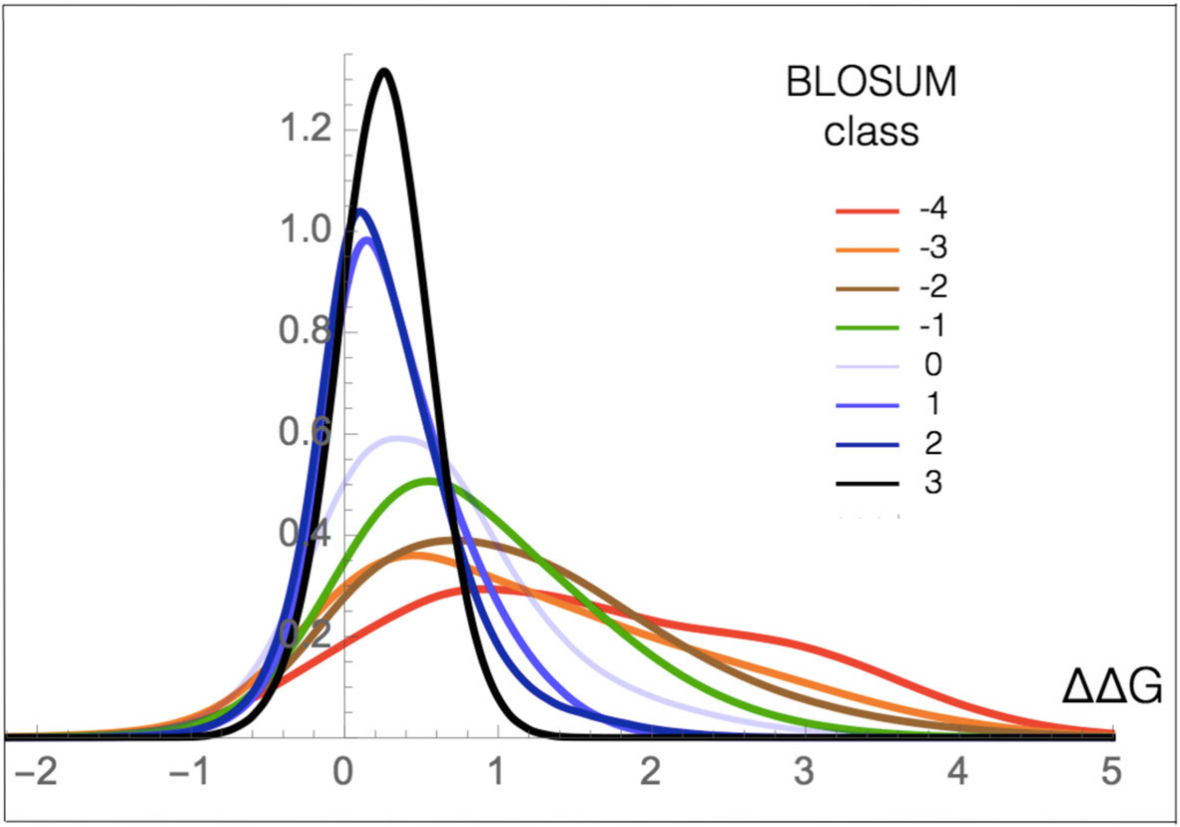
*ΔΔG* distribution (in kcal/mol) of all *μ*SBSs in $\mathcal {M}_{\text {PoP}}$ as a function of the BLOSUM62 score. Positive BLOSUM scores indicate more likely amino acid substitutions and negative scores, less likely substitutions

The relation between mutational robustness and BLOSUM scores is clearly seen in Fig. 3: the *ΔΔG* distribution extends more and more toward positive values—i.e. toward destabilizing mutations—when the BLOSUM62 score decreases.

### Structure of the genetic code

We investigated the relation between the mutational robustness and the structure of the standard genetic code. In the codon-to-amino acid mapping, single base substitutions lead to some but not all amino acid mutations. To get them all, the simultaneous substitution of two or three bases has to be considered, which occur at a much lower rate.

We thus compared the mutational *ΔΔG* profiles of single versus multiple base substitutions (*μ*SBSs versus *μ*MBSs) to better understand the extent to which the standard genetic code is optimized to ensure mutational robustness. Note that we call *μ*MBS, amino acid mutations that cannot be reached by any SBS.

**Fig. 4.**
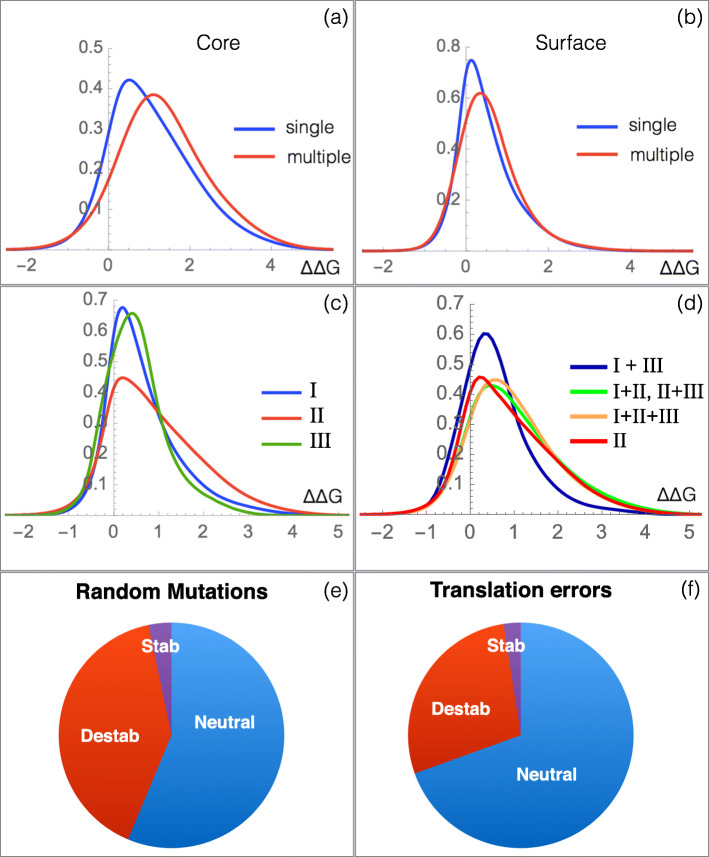
Effects of single and multiple base substitutions and the nucleobase position in the codon. **a**–**d** *ΔΔG* distribution (in kcal/mol) of amino acid mutations in $\mathcal {M}_{\text {PoP}}$. **a** *μ*SBSs and *μ*MBSs in the core (RSA ≤20*%*) and **b** on the surface (RSA >20*%*). **c** *μ*SBSs resulting from substitutions of bases I, II, or III in the codon. **d** *μ*MBSs resulting from simultaneous substitutions of two or three bases in the codon. Ratio of stabilizing, destabilizing, and neutral mutations considering random mutations (that occur with equal frequency at each codon position) **e**, or considering translation errors (that occur with different frequency at each codon position) **f**. Note that in **e**, **f**, the synonymous mutations and mutation degeneracy are included in the computations

First of all, we found that mutations resulting from single base substitutions are on average less destabilizing than those resulting from multiple base substitutions, for both the core and surface regions (Fig. 4a, b and Table 3, and Additional file 1: Table S1 and Figure S5 for more detailed RSA dependence). This suggests that the structure of the standard genetic code is optimized, at least partially, for protein mutational robustness through the minimization of the destabilizing impact of random mutations.

**Table 3:** Comparison between the mean *ΔΔG* values (in kcal/mol) of single and multiple nucleotide substitutions (*μ*SBS and *μ*MBS) in $\mathcal {M}_{\text {PoP}}$ and the fraction of stabilizing, neutral and destabilizing mutations. Core residues have an RSA ≤20*%* and surface residues an RSA >20*%*

Region	〈*ΔΔG*〉	Stab (%)	Neut (%)	Dest (%)
*μ*SBS				
All	0.81	4	41	55
Core	1.09	4	28	68
Surface	0.49	4	55	41
*μ*MBS				
All	0.97	4	32	64
Core	1.35	4	18	78
Surface	0.56	5	47	48

However, a deeper investigation leads to nuance this view. Indeed, there is a large difference according to which bases in the codon are substituted, as seen in Fig. 4c, d and Table 4. We denote as I, II, and III the three bases in the codons.

**Table 4:** Mean *ΔΔG* (in kcal/mol) for *μ*SBSs from $\mathcal {M}_{\text {PoP}}$ obtained from SBSs at different codon positions (I, II, III). In the lower part of the table, the mean *ΔΔG* is computed by considering also the synonymous mutations (with *ΔΔG*=0) and the degeneracy (the number of SBSs leading to a *μ*SBS)

Position	〈*ΔΔG*〉	Stab (%)	Neut (%)	Dest (%)
Without synonymous mutations and degeneracy				
I	0.65	3	49	48
II	0.91	5	36	59
III	0.51	4	50	46
With synonymous mutations and degeneracy				
I	0.64	3	50	47
II	0.91	5	36	59
III	0.16	2	84	14

Clearly, the substitution of base II in the codon yields the most destabilizing amino acid mutations, on average. At the other extreme, the least destabilizing SBSs involve base III, followed by base I. This is related to the structure of the genetic code and the smallest physicochemical property changes caused by base III substitutions and the largest changes caused by base II substitutions. Again, the trends are more pronounced for core than for surface residues (Additional file 1: Table S1 and Figure S5).

An important result is that we find the same trends with experimental stability values from $\mathcal {M}_{\text {Exp}}$ than with computed values from $\mathcal {M}_{\text {PoP}}$, as shown in Additional file 1: Table S2.

Moreover, only 14 amino acid mutations are reachable by varying base III, against 64 for base I, and 80 for base II, as can be deduced by looking at the genetic code table (Additional file 1: Figure S1). Thus, not only is base III the most optimized for stability, but it is also the base that leads to the lowest number of non-synonymous mutations. Base II is the least optimized for stability and moreover leads to the highest number of non-synonymous mutations.

As a consequence, the difference between the three base substitutions is even clearer when including the synonymous mutations in the estimation of the mean *ΔΔG*, which consist of base substitutions that lead to the same amino acid and thus to *ΔΔG* values equal to zero. We have in that case also to count the degeneracy, that is the number of different base substitutions that yields the same amino acid mutation. The results are shown in Table 4: the mean *ΔΔG* is lowest for base III (0.16 kcal/mol), medium for base I (0.64 kcal/mol), and highest for base II (0.91 kcal/mol). Analogous differences can be observed at any values of the solvent accessibility and become even more important in the core while decrease at the surface (Additional file 1: Table S1).

So, there seems to be a stronger positive selection pressure on base I and even more on base III, whereas base II appears much more constrained across evolution. This has sometimes been related to the origin of the genetic code and considered as a by-product of the expansion of the primitive code through the diversification of the amino acids repertoire [61, 62]. Another interpretation is more straightforward in the present context: our results are related to the codon-anticodon pairing and mispairing in the translation process. Indeed, transfer RNA reads with much higher accuracy base II in the codon than base I and even more, than base III [22, 63]. However, whether the standard genetic code has adapted to the translation machinery or *vice versa* is impossible to know at this stage.

Our results can thus be taken to mean that natural selection, through targeted adaptation of the standard genetic code and/or the translation machinery, primarily favors an increased translation accuracy, rather than the minimization of the impact of random mutations.

This interpretation is supported by the finding of a high anticorrelation between the mean *ΔΔG* per position in the codon and the frequency of the translation error at these positions; these frequencies are equal to (31.3%, 6.2%, 62.5%) [25]. Indeed, the Pearson’s linear correlation coefficient is almost perfect: *r*=−0.996 (*P* value ≃0.05).

We also compared the impact of single and multiple nucleotide substitutions (Fig. 4c, d and Additional file 1: Table S1 and Figure S5). We found that the *ΔΔG* profile obtained from *μ*MBSs of the two bases I+III are less destabilizing than base II *μ*SBSs and only slightly more destabilizing than base I or base III *μ*SBSs. Furthermore, the *ΔΔG* profile of base II *μ*SBSs strongly resembles the profiles of bases I+II and bases II+III *μ*MBSs.

In summary, we have the following increased destabilization ranking: III, I, I+III, II, II+III, I+II, I+II+III. The comparison of these results with the frequency of translation errors yields a very interesting result that further confirms our hypotheses: the anticorrelation between the mean *ΔΔG* and the frequency of the translation errors for all these different types of substitutions is extremely high *r*=−0.951 (*P* value <0.001).

Finally, we computed the fraction of stabilizing, destabilizing and neutral mutations according to whether they result from random mutations or from errors in translation. In the latter case, the frequencies of the translation errors at the three positions in the codon must be taken into account. As shown in Fig. 4e, f, a much larger number of neutral mutations and a reduced fraction of destabilizing mutations are found if we consider translation errors. This trend is even more pronounced in the core, as seen in Additional file 1: Figure S6.

This result signals a better optimization of the standard genetic code for minimizing the consequences of errors in translation. It is also optimized to minimize the effects of random mutations in the DNA, but to a lesser extent; indeed, random mutations occur with equal frequency at the three codon positions.

The error rates are known to be of the order of 10^−8^ in genome replication with a substantial variation as a function of the organism and of the order of 10^−5^ in transcription. Instead, the error rate in protein synthesis is higher with a value of about 10^−4^. This suggests that the mRNA translation process is the real bottleneck in proteome accuracy maintenance [64, 65] and explains our finding that the standard genetic code evolved to primarily favor robustness against mutations caused by defaults in the translation machinery.

### Nucleotide composition

Let us now study the mutational robustness as a function of the nucleotide composition of the mRNA sequence, which is often biased and varies from GC- to AT-rich. The GC-content influences the amino acid composition of the encoded protein and has even been used to predict the amino acid frequencies. For low or high GC content, mutual evolutionary adjustments between genomic GC content and amino acid composition are observed [20]. Moreover, the GC-content, especially at the third position in the codon, has been shown to correlate with the hydrophobic amino acid content and thus with protein stability [66–68], as well as with gene expression efficiency in mammalian cells [69]. However, the relative weight of these different effects is still debated [70].

**Fig. 5.**
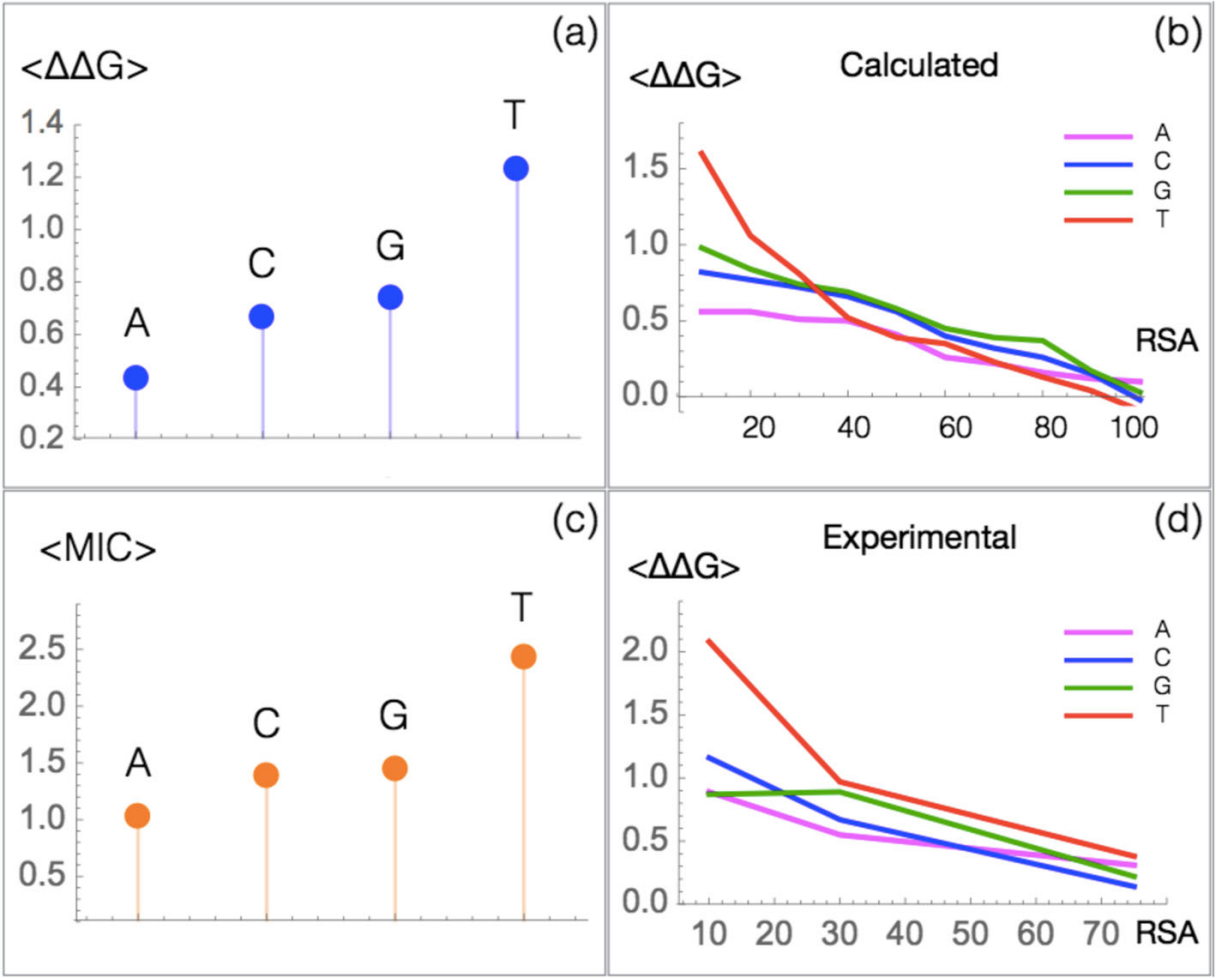
Mean *ΔΔG* (in kcal/mol) and fitness of amino acid mutations caused by the substitution of each of the four nucleobases. **a** Computed 〈*ΔΔG*〉 of SBSs in the ${\mathcal {M}_{\text {PoP}}}$ set. **b** Computed 〈*ΔΔG*〉 of SBSs as a function of the residue solvent accessibility (RSA) in the $\mathcal {M}$PoP set. **c** Fitness score of mutations measured via the minimum inhibitory concentration (MIC) to *β*-lactam amoxicillin [57]. **d** Computed 〈*ΔΔG*〉 of SBSs as a function of the RSA in the $\mathcal {M}_{\text {Exp}}$ set. Note that here only three RSA bin (0–20%, 20–50%, 50–100%) are considered due to the limited number of entries

Here, we investigated the protein mutational robustness as a function of the mutated nucleotide type by estimating the mean *ΔΔG* of *μ*SBSs resulting from the substitution of each of the four nucleobases, independently of their position in the codon (Table 5, Fig. 5a and Additional file 1: Table S3). We observed that substitutions of A yields the most robust amino acid mutations and substitutions of T the least robust mutations. C and G show similar intermediate behaviors. The same trends are observed for experimental mutations from $\mathcal {M}_{\text {Exp}}$ (Additional file 1: Table S4).

**Table 5:** Mean *ΔΔG* (in kcal/mol) and fraction of stabilizing, neutral and destabilizing *μ*SBSs from $\mathcal {M}_{\text {PoP}}$ which result from the substitution of one of the four nucleobases

Base	〈*ΔΔG*〉	Stab (%)	Neut (%)	Dest (%)
A	0.43	6	55	39
C	0.67	3	41	55
G	0.74	5	46	49
T	1.23	2	27	71

The low robustness of T is not surprising as it is the least frequently substituted base in exome regions [70, 71] and there is thus a strong evolutionary pressure acting on it. In contrast, the high robustness of A is a priori surprising since it is usually less frequently mutated than G and C bases.

In fact, the differences between the four bases are mainly observed in the protein core. This can be clearly seen from Fig. 5b, d, where the mean *ΔΔG* as a function of the solvent accessibility is plotted for each kind of wild-type nucleobase, for computed and experimental *ΔΔG*s.

We can thus hypothesize that these differences are linked to the hydrophobicity of the encoded amino acids [72]. This is indeed the case: the T content of the codons is correlated with the hydrophobicity of the encoded amino acids (*r* = 0.55 using the hydrophobicity scale of Kyte-Doolittle, *P* value <10^−7^), the A content is anticorrelated with it (*r* = − 0.28, *P* value <0.005). No correlation is observed for C and G.

Thus, T-containing codons code preferentially for hydrophobic amino acids, and their mutations in the core induce on average strong destabilization. In contrast, A-containing codons tend to encode polar amino acids, and their mutations in the core are often neutral or stabilizing. This explains the observed mutational robustness profile upon specific base substitutions and the absence of link with the rate of SBSs in exome regions [71].

To better assess our observations, we compared them with the mutagenesis data from $\mathcal {M}_{\text {Fit}}$. We found a nice agreement between our stability predictions (Fig. 5a) and the experimental fitness data of TEM-1 *β*-lactamase [57] as measured via the minimum inhibitory concentration (MIC) to *β*-lactam amoxicillin (Fig. 5c). A similar agreement was found with the other fitness data from $\mathcal {M}_{\text {Fit}}$ (Additional file 1: Figure S7).

### Transition to transversion bias

Transitions are substitutions that interchange purines (A ⇔G) or pyrimidines (C ⇔T), whereas transversions interchange purines and pyrimidines (C,T ⇔A,G). Transitions are known to be from 2 to 5 times more frequent than transversions [73, 74], an observation called the transition to transversion bias. However, the origin of this bias is a longstanding problem in molecular evolution.

Recently, the relationship between this bias and the fitness score was analyzed on a set of about 1,200 mutations, in which a probability of 53% was found for the transitions to be fitter than the transversions [74]. However, this tiny difference cannot justify the large bias observed in evolutionary investigations and thus essentially discard a selection effect as main explanation.

In another recent study [75], transitions were seen to be significantly less detrimental than transversions in deep mutagenesis scanning experiments on the influenza and HIV viruses. This suggests instead that the selective hypothesis cannot be totally ruled out, but that it could contribute, together with other mutational biases, to explain the observed transition to transversion substitution rate.

Our results are basically in agreement with the first aforementioned analysis. Indeed, we found the transitions to be slightly more robust than transversions, with a mean 〈*ΔΔG*〉 of 0.51 and 0.60 kcal/mol, respectively, when considering the mutation degeneracy and the synonymous mutations. However, this free energy difference is too small to explain the large bias observed.

Note that, if only the non-synonymous mutations are included in the 〈*ΔΔG*〉 computation, the opposite trend is observed, both using computed and experimental stability data (Table 6 and Additional file 1: Tables S5 and S6). This is due to the fact that transitions are enriched in synonymous mutations.

**Table 6:** Mean *ΔΔG* (in kcal/mol) for *μ*SBSs from $\mathcal {M}_{\text {PoP}}$ obtained from transitions and transversions. In the upper part of the table, the mean *ΔΔG* includes the synonymous mutations (with *ΔΔG*=0), while the lower part is without them

Mutation	〈*ΔΔG*〉	Stab (%)	Neut (%)	Dest (%)
With synonymous mutations				
Transitions	0.51	2	63	35
Transversions	0.60	4	53	43
Without synonymous mutations				
Transitions	0.79	3	44	53
Transversions	0.73	5	43	52

### Codon usage

The understanding of the codon usage and its evolution are strongly debated in the molecular evolution field. Indeed, the codon usage is intrinsically connected with a wide range of factors whose contributions are difficult to disentangle [76]. For example, relations of codon usage with tRNA abundance, translation elongation rate, protein expression levels, and stability of mRNA secondary structure have been observed, which suggests an explanation in terms of selection for translation efficiency [77–79].

Another interesting hypothesis is that codon usage is shaped to minimize errors at the protein level. This adaptive hypothesis suggests that a selective pressure for mutational robustness acts on codon usage to reduce the deleterious impacts of genetic variants [30, 31, 34, 80–82]. In [83], the comparison between wild type and engineered capsid poliovirus, in which synonymous mutations are introduced, suggests that the former has a higher mutational robustness than the latter, and thus that codon choice is directly connected to robustness.

Codon usage could also be related to protein evolvability, since synonymous codons allow the exploration of different evolutionary pathways displaying different sets of proximal amino acid mutations [29].

In order to deepen the hypothesis of the role of the codon usage in minimizing errors at the protein level, we compared the mutational robustness of proteins when using the actual codon or synonymous codons. More specifically, we analyzed how the 〈*ΔΔG*〉 that results from random mutations or translation errors differs according to the codon usage. We also analyzed the 〈*ΔΔG*〉 at each codon position to study a possible position-dependent codon selection.

The difference in 〈*ΔΔG*〉 when using the actual or a synonymous codon is reported in Table 7 and Additional file 1: Table S7 for predicted stability values and in Additional file 1: Table S8 for experimental ones. We observe that the used codons lead in general to higher robustness than synonymous ones. The difference can amount to about 10% of the standard deviation of the *ΔΔG* distributions. This effect is apparent for mutations inserted at each of the three positions in the codon, although to a different extent, and both for random mutations, which do not distinguish between the positions in the codon, and for translation errors, in which the error rate depends on the position.

**Table 7:** Difference between 〈*ΔΔG*〉 for *μ*SBSs in $\mathcal {M}_{\text {PoP}}$ reached from synonymous codons (syn) or from the wild-type codon (used), according to the position of the substituted base in the codon (I, II and III), and according to whether the position-dependent frequency of translation errors is taken into account (translation) or not (random). *σ* is the standard deviation of the *ΔΔG* distribution: *σ*^2^(*ΔΔG*)=*σ*^2^(*ΔΔG*^used^)+*σ*^2^(*ΔΔG*^syn^)

	(〈*ΔΔG*^syn^〉−〈*ΔΔG*^used^〉)/*σ*		
	All (%)	Core (%)	Surface (%)
I	3	− 2	11
II	7	6	6
III	11	9	8
Random	7	3	9
Translation	6	4	7

**Fig. 6.**
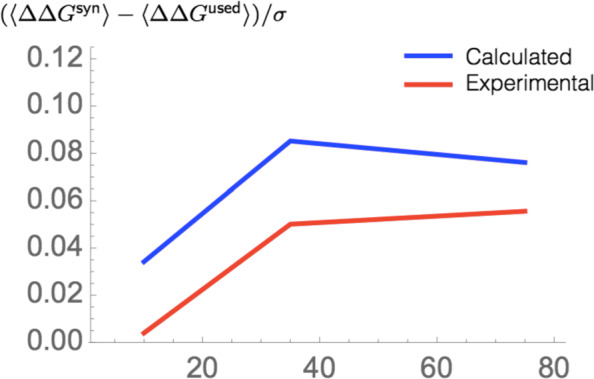
Difference between 〈*ΔΔG*〉 for *μ*SBSs in $\mathcal {M}_{\text {PoP}}$ and $\mathcal {M}_{\text {Exp}}$ reached from synonymous codons (syn) or from the used codon (used) as a function of the RSA

Interestingly, the higher robustness of used compared to synonymous codons is on average smaller for core residues and bigger for surface residues (Fig. 6, Table 7 and Additional file 1: Tables S7 and S8). It has however to be noted that this trend is basically due to substitutions at codon position I; no difference is observed for substitutions at positions II or III when compared to the standard deviation of the *ΔΔG* distribution. It has to be underlined that we obtained this result for both experimental and predicted stability values (Additional file 1: Tables S7 and S8). The observed difference between mutations on the surface and in the core could be related to the fact that the former evolve faster than the latter.

Another interesting result is that the codon choice seems to minimize to the same extent the impacts of translation errors and of random mutations.

We also analyzed the C+G-content in the codons. We found that the difference in CG content between used and synonymous codon is equal to be about − 4%. This indicates that codons with higher CG content have a slightly higher chance to be used. Several reasons have been proposed to explain this bias [84], among which improved mRNA translation efficiency [69].

### Codon usage bias

Some codons occur much more frequently than others, and this effect, known as the codon usage bias, strongly depends on the host organism [85]. This bias has been related to the tRNA pool in the organisms; indeed a correlation between the codon frequency and the concentration of tRNAs with the complementary anticodons has been found in many genomes. This correlation could contribute to the efficiency of the translational process by tuning the elongation rate [86–88].

We analyzed here whether there is a link between codon choice, codon usage bias, and mutational robustness. More precisely, we investigated if the used codon is better optimized for mutational robustness than synonymous codons in the biased or unbiased subsets of codons.

To explore this question, we retrieved the codon usage frequency tables [89] of the host organisms of the proteins from the structurome set $\mathcal {D}$, and defined codons as biased if their frequency deviates by more than 12.5% from equiprobability [89]. We then compared the 〈*ΔΔG*〉 of *μ*SBSs reached from synonymous and used codons, according to whether these codons are biased or not in the protein’s host organism.

For unbiased codons, the wild-type and synonymous codons appear to have basically the same mutational robustness (Table 8). In contrast, for biased codons, the used codons clearly appear to be more robust than the synonymous ones.

**Table 8:** Difference between 〈*ΔΔG*〉 for *μ*SBSs in $\mathcal {M}_{\text {PoP}}$ reached from synonymous codons (syn) or from the wild-type codon (used), according to whether the position-dependent frequency of translation errors is taken into account (translation) or not (random). *σ* is the standard deviation of the *ΔΔG* distribution (see legend to Table 7)

	(〈*ΔΔG*^syn^〉−〈*ΔΔG*^used^〉)/*σ*	
	Biased (%)	Unbiased (%)
Random	8	0
Translation	8	1

Note that we also dropped the assumption of equiprobability of the four nucleobases and defined the codon usage as biased or unbiased on the basis of the deviation from the expected codon frequencies calculated from the observed nucleotide frequencies in the specific host organism [89]. We found similar though slightly less pronounced trends, as shown in Additional file 1: Table S9.

This interesting result suggests that the codon usage bias is not only related to the optimization of the translation efficiency, but also to increase the mutational robustness. It points out the non-trivial role of the selection for error minimization at the protein level in shaping the codon usage, in agreement with an adaptationist hypothesis [31]. Here, for the first time, we quantified these effects that certainly play an important role in the highly complex interdependence with other factors, such as translation elongation speed, initiation efficiency, and mRNA stability. These interrelationships need to be further explored.

### Outliers

We would like to emphasize that all the above results represent average tendencies. Additional insights can be gained from analyzing proteins or protein regions which deviate from these average tendencies. For example, we found that the least mutationally robust protein in $\mathcal D$ is wheat agglutinin isolectin 3 (PDB code 2X52). The average *ΔΔG* over all its *μ*SBSs is equal to 1.08 kcal/mol, which is much higher than the average value of 0.81 kcal/mol (Table 3). A closer look at this protein shows that it has many disulfide bridges: 16 for 170 residues. Computing separately the *ΔΔG* values of residues involved in disulfide bridges and of those that are not yields 〈*ΔΔG*〉 values of 2.07 and 0.84 kcal/mol, respectively. The per-residue 〈*ΔΔG*〉 is shown in Additional file 1: Figure S8.

As expected, mutations affecting disulfide bridges are strongly destabilizing, which makes this protein much less robust against mutations on average than other proteins. The lower robustness of disulfide bridge-containing proteins can be expected to be a general result. We will perform a systematic analysis of outliers at large scale in a forthcoming study.

## Conclusion

The mutational robustness of proteomes and its adaptation across natural evolution are key questions in protein science. Answering these would be proficuous not only for fundamental understanding but also for a wide range of biotechnological and biopharmaceutical applications. To deepen this issue, we investigated here how a series of factors influences protein mutational robustness through large-scale in-silico deep-mutagenesis scanning experiments and the analysis of experimental mutagenesis data.

A first point to emphasize is that, whenever the amount of experimental data is sufficient, experimental and computed results largely coincide. This strongly supports the accuracy and unbiased nature of our predictions and the validity of our structurome-scale approach. The good generalization properties of our predictor are further detailed in the “Methods” section.

### Summary of the results and importance of the 3D structure

Our results can be summarized as follows:

- Core residues are much less robust on average and evolve slower than mutations on the surface, as they are more structurally constrained.
- Short proteins have a less robust core and a more robust surface than longer proteins, as they have larger proportions of buried hydrophobic residues and of exposed functional residues.
- The fraction of stabilizing mutations is almost identical on the surface and in the core (about 4%), the fraction of neutral mutations is higher on the surface, and the fraction of destabilizing mutations is higher in the core. They nicely agree with the fractions of beneficial, neutral, and deleterious mutations estimated in experimental mutagenesis studies. This result supports the pivotal role of thermodynamic stability in the fitness cost of mutations [51].
- The mean frequency of substitutions across evolution, characterized by the BLOSUM62 matrix, is highly correlated with their mutational robustness: rare substitutions are on average more destabilizing that frequent ones.
- Single base substitutions are on average less destabilizing for the protein than multiple base substitutions, which occur at a much lower rate. This led to the first conclusion that the standard genetic code evolved to minimize the errors of random mutations and to preserve the genome information at all stages, from DNA replication and transcription to mRNA translation and protein synthesis.
- Not all bases in the codon are optimized in the same way. The mean robustness upon single and multiple base substitutions decreases according to the following ranking: base III, I, I+III, II, II+III, I+II, and I+II+III. Notably, the corresponding 〈*ΔΔG*〉 values are almost perfectly anticorrelated with the frequency of translation errors. The genetic code is thus primarily optimized to limit mRNA translation errors. As these errors are more frequent than transcription and replication errors, their minimization can be viewed as an overall optimization of the genetic material encoding.
- Wild-type codons are on average more robust than synonymous codons, in the sense that SBSs of the wild-type codon yield less destabilizing amino acid mutations. The codon is thus selected, at least partly, to minimize the effect of both transcriptional and translational errors. Note that our results show that the codon usage is partially optimized for the precision of translation. This effect adds to the codon optimization for translation efficiency and for mRNA stability [64, 77–79].
- The codon selection for mutational robustness seems on average stronger at the protein surface, where the substitution rate is higher and thus where natural selection has had more opportunities for optimization.
- The codon selection for mutational robustness is stronger for biased than for unbiased codons, suggesting that also the codon usage bias could be partly due to mutational robustness optimization.

We would like to underline that the use of 3D structural information is a fundamental piece in our analyses, which allowed us to gain a deeper understanding of the link between thermodynamic constraints and natural evolution. We believe that this is a general trend, and that the integration of structure and sequence data is needed to further improve our understanding of the evolutionary mechanisms and how the biophysical features shape and are shaped by them.

### Perspectives

Different questions still need to be addressed. A first issue is the origin of the mutational robustness and whether it can be considered as an emergent property or a property that depends on an intricate combination of factors, some of which are analyzed in this paper [90]. Other biophysical features such as protein dynamics, conformational disorder, and thermal stability as well as environmental and life-style variables such as the type of organism, the organism growth temperature (OGT), and the aerobic or anaerobic environment should be explored and integrated in the current analysis to better understand the mutational robustness and its highly complex dependencies. For example, using large-scale experimental data about protein melting temperatures [91] and OGT [92], it would be very interesting to further investigate how robustness with respect to mutations is related to these two quantities.

## Methods

### Protein structurome

The non-redundant set $\mathcal {D}$ of protein structures analyzed here, which represent the structurome, was obtained by following steps, schematically depicted in Additional file 1: Figure S9:

1. We used the PISCES protein culling server [93] to get the subset of proteins from the Protein Data Bank (PDB) [17] which have an experimental X-ray structure of at most 2.5Å resolution and share less than 95% pairwise sequence identity. We considered each of these proteins in the context of their biological unit referenced in the PDB.
2. We filtered out the membrane proteins, viral capsid proteins, and antibodies on the basis of PDB annotations. The first series of proteins is overlooked because the PoPMuSiC ^sym^ predictor is applicable to globular proteins only, the second series because they form very large oligomeric assemblies, and the last because antibodies have variable sequences and the mutations in their complementarity determining regions have a clear functional role. We obtained in this way a uniform set of globular proteins.
3. For each protein entry, we retrieved the DNA sequence from the EMBL webserver [94, 95].
4. To check the protein-DNA mapping, we aligned the translated DNA sequences with the protein sequences from the PDB using CLUSTALW [96]. We kept only the DNA sequences which yield at least 95% sequence identity with the PDB sequences.

With this procedure, we obtained 21,540 X-ray structures amounting to 5,368,279 residues in total. The list of organisms to which these proteins belong, the total protein number per organism and the average *ΔΔG* of all possible single-site mutations are listed in Additional file 1: Tables S10-S13. The code for generating and analyzing the data is available in our GitHub repository [97].

### Large-scale in silico mutagenesis experiments

We estimated the folding free energy changes (*ΔΔG*) caused by all possible single-site mutations introduced in all collected structures, using the unbiased version of our in-house predictor PoPMuSiC, called PoPMuSiC ^sym^ [35, 36]. The set of mutations so obtained is called $\mathcal {M}_{\text {PoP}}$. It contains 101,997,301 mutations among which 100,149,646 have a known wild-type codon. Note that mutations from or to stop codons are not taken into account.

The model structure of PoPMuSiC ^sym^ consists of a linear combination of energy terms estimated using different types of statistical potentials. These have been derived from frequencies of sequence-structure associations in sets of protein X-ray structures, transformed into folding free energies using the Boltzmann law. The coefficients of the linear combinations are sigmoid functions of the RSA of the mutated residues. The PoPMuSiC model is based on the assumptions that the structure of the native state is only slightly modified upon mutations and that the stability of the reference state mimicking an unfolded state remains unchanged.

We refer to [35, 36] for further technical details about the PoPMuSiC ^sym^ predictor.

### PoPMuSiC ^sym^ and its generalization properties

The question of how much predictions of a data-driven computational tool such as PoPMuSiC ^sym^ are not overfitted or overly biased toward the training set is a longstanding issue in computational biology, often called the generalization problem [98]. Extensive tests assessing PoPMuSiC ^sym^’s generalization properties have been performed during its validation phase, such as *N*-fold cross-validation and application to independent test sets [35, 36]. To avoid overestimating the performance, we carefully checked that there was no similarity between the folds and between training and test sets.

Another source of prediction biases can be hidden in the content of the training set. For example, training sets are usually strongly enriched in destabilizing mutations, with the consequence that most predictors are biased toward these mutations. We carefully checked that PoPMuSiC ^sym^ does not show this bias [35] by training it on three sets of mutations: a set with a majority of stabilizing mutations, a set with a majority of destabilizing mutations and a set with an equal number of stabilizing and destabilizing mutations. These three PoPMuSiC ^sym^ versions gave very similar predictions when applied on test sets, which demonstrates their independence on the training set.

We would like to emphasize that the good generalization capacity of PoPMuSiC ^sym^ is one of its strengths, which is not achieved by all stability predictors [36]. Nevertheless, it is important to acknowledge that it is never possible to completely rule out the presence of hidden biases at the residue and protein levels. However, biases related to the genetic code, the codon usage and the codon bias are highly unlikely as PoPMuSiC ^sym^ does not use any of these features in its model construction and training. It is indeed based only on the proteins’ amino acid sequence and structure. Our results at the codon and nucleobase levels can thus not be attributed to statistical biases or overfitting, which is what makes them even more interesting.

### Experimentally characterized stability changes

We also considered the set of 2,648 mutations of which the *ΔΔG* folding free energy change upon single-site mutations has been experimentally measured. This set, that we call $\mathcal {M}_{\text {Exp}}$, was manually curated as described in [99] and was further annotated according to the previously described pipeline. It has been used to train the PoPMuSiC predictors. The list of mutations of $\mathcal {M}_{\text {Exp}}$ can be found in the supplementary material of [99].

## Supplementary information

**Additional file 1** Large-scale in-silico mutagenesis experiments reveal optimization of genetic code and codon usage for protein mutational robustness

## Data Availability

The list of organisms to which the proteins in the dataset $\mathcal {D}$ belong, the total protein number per organism, and the average *Δ**Δ**G* of all possible single-site mutations are listed in Additional file 1: Tables S10-S13. The code for generating and analyzing the data is available on our GitHub repository [97].

## References

[1] Kimura M (1968). Evolutionary rate at the molecular level. Nature.

[2] Achoch M, Dorantes-Gilardi R, Wymant C, Feverati G, Salamatian K, Vuillon L, Lesieur C (2016). Protein structural robustness to mutations: an in silico investigation. Phys Chem Chem Phys.

[3] Bloom JD, Labthavikul ST, Otey CR, Arnold FH (2006). Protein stability promotes evolvability. Proc Natl Acad Sci USA.

[4] Lenski RE, Barrick JE, Ofria C (2006). Balancing robustness and evolvability. PLoS Biol.

[5] Bloom JD, Lu Z, Chen D, Raval A, Venturelli OS, Arnold FH (2007). Evolution favors protein mutational robustness in sufficiently large populations. BMC Biol.

[6] Serohijos AW, Rimas Z, Shakhnovich EI (2012). Protein biophysics explains why highly abundant proteins evolve slowly. Cell Rep.

[7] Sikosek T, Chan HS (2014). Biophysics of protein evolution and evolutionary protein biophysics. J R Soc Interface.

[8] Wagner A (2008). Robustness and evolvability: a paradox resolved. Proc R Soc B.

[9] Tokuriki N, Tawfik DS (2009). Protein dynamism and evolvability. Science.

[10] Tokuriki N, Tawfik DS (2009). Stability effects of mutations and protein evolvability. Curr Opin Struct Biol.

[11] Lassig M, Mustonen V, Walczak AM (2017). Predicting evolution. Nat Ecol Evol.

[12] Bloom JD, Silberg JJ, Wilke CO, Drummond DA, Adami C, Arnold FH (2005). Thermodynamic prediction of protein neutrality. Proc Natl Acad Sci USA.

[13] Besenmatter W, Kast P, Hilvert D (2007). Relative tolerance of mesostable and thermostable protein homologs to extensive mutation. Proteins.

[14] Draghi JA, Parsons TL, Wagner GP, Plotkin JB (2010). Mutational robustness can facilitate adaptation. Nature.

[15] van Nimwegen E, Crutchfield JP, Huynen M (1999). Neutral evolution of mutational robustness. Proc Natl Acad Sci USA.

[16] Bloom JD, Raval A, Wilke CO (2007). Thermodynamics of neutral protein evolution. Genetics.

[17] Berman HM, Westbrook J, Feng Z, Gilliland G, Bhat TN, Weissig H, Shindyalov IN, Bourne PE (2000). The protein data bank. Nucleic Acids Res.

[18] Bastolla U, Porto M, Roman HE, Vendruscolo M (2005). Looking at structure, stability, and evolution of proteins through the principal eigenvector of contact matrices and hydrophobicity profiles,. Gene.

[19] Goncearenco A, Ma BG, Berezovsky IN (2014). Molecular mechanisms of adaptation emerging from the physics and evolution of nucleic acids and proteins,. Nucleic Acids Res.

[20] Goncearenco A, Berezovsky IN (2014). The fundamental tradeoff in genomes and proteomes of prokaryotes established by the genetic code, codon entropy, and physics of nucleic acids and proteins. Biol Direct.

[21] Ma BG, Goncearenco A, Berezovsky IN (2010). Thermophilic adaptation of protein complexes inferred from proteomic homology modeling. Structure.

[22] Haig D, Hurst LD (1991). A quantitative measure of error minimization in the genetic code. J Mol Evol.

[23] Epstein CJ (1966). Role of the amino-acid “code” and of selection for conformation in the evolution of proteins. Nature.

[24] Goldberg AL, Wittes RE (1966). Genetic code: aspects of organization. Science.

[25] Freeland SJ, Hurst LD (1998). The genetic code is one in a million. J Mol Evol.

[26] Di Giulio M, Medugno M (1999). Physicochemical optimization in the genetic code origin as the number of codified amino acids increases. J Mol Evol.

[27] Gilis D, Massar S, Cerf NJ, Rooman M (2001). Optimality of the genetic code with respect to protein stability and amino-acid frequencies. Genome Biol..

[28] Wnȩtrzak M, BłaŻej P, Mackiewicz D, Mackiewicz P (2018). The optimality of the standard genetic code assessed by an eight-objective evolutionary algorithm. BMC Evol Biol.

[29] Cambray G, Mazel D (2008). Synonymous genes explore different evolutionary landscapes. PLoS Genet.

[30] Archetti M (2004). Selection on codon usage for error minimization at the protein level. J Mol Evol.

[31] Archetti M (2006). Genetic robustness and selection at the protein level for synonymous codons. J Evol Biol.

[32] Ikemura T (1981). Correlation between the abundance of *Escherichia coli* transfer RNAs and the occurrence of the respective codons in its protein genes: a proposal for asynonymous codon choice that is optimal for the *E. coli* translational system,. J Mol Biol.

[33] Ikemura T (1985). Codon usage and tRNA content in unicellularand multicellular organisms,. Mol Biol Evol.

[34] Akashi H (1994). Synonymous codon usage in Drosophila melanogaster: natural selection and translational accuracy. Genetics.

[35] Pucci F, Bernaerts KV, Teheux F, Gilis D, Rooman M (2015). Symmetry principles in optimization problems: an application to protein stability prediction. IFAC-PapersOnLine.

[36] Pucci F, Bernaerts KV, Kwasigroch JM, Rooman M (2018). Quantification of biases in predictions of protein stability changes upon mutations. Bioinformatics.

[37] Gilis D, Rooman M (1997). Predicting protein stability changes upon mutation using database-derived potentials: solvent accessibility determines the importance of local versus non-local interactions along the sequence. J Mol Biol.

[38] Tokuriki N, Stricher F, Schymkowitz J, Serrano L, Tawfik DS (2007). The stability effects of protein mutations appear to be universally distributed. J Mol Biol.

[39] Faure G, Koonin EV (2015). Universal distribution of mutational effects on protein stability, uncoupling of protein robustness from sequence evolution and distinct evolutionary modes of prokaryotic and eukaryotic proteins. Phys Biol.

[40] Dehouck Y, Gilis D, Rooman M (2004). Database-derived potentials dependent on protein size for in silico folding and design. Biophys J.

[41] Bastolla U, Demetrius L (2005). Stability constraints and protein evolution:the role of chain length, composition and disulfide bonds. Protein Eng Des Sel.

[42] Minning J, Porto M, Bastolla U (2013). Detecting selection for negative design in proteins through an improved model of the misfolded state,. Proteins.

[43] Arenas M, Sánchez-Cobos A, Bastolla U (2015). Maximum-likelihood phylogenetic inference with selection on protein folding stability,. Mol Biol Evol.

[44] De Laet M, Gilis D, Rooman M (2016). Stability strengths and weaknesses in protein structures detected by statistical potentials: Application to bovine seminal ribonuclease. Biophys J.

[45] Freiberger MI, Guzovsky AB, Wolynes PG, Parra RG, D.U. F (2019). Local frustration around enzyme active sites. Proc Natl Acad Sci U S A.

[46] Franzosa EA, Xia Y (2012). Independent effects of protein core size and expression on residue-level structure-evolution relationships. PLoS ONE.

[47] Ramsey DC, Scherrer MP, Zhou T, Wilke CO (2011). The relationship between relative solvent accessibility and evolutionary rate in protein evolution. Genetics.

[48] Yeh SW, Liu JW, Yu SH, Shih CH, Hwang JK, Echave J (2014). Site-specific structural constraints on protein sequence evolutionary divergence: local packing density versus solvent exposure. Mol Biol Evol.

[49] Franzosa EA, Xia Y (2009). Structural determinants of protein evolution are context-sensitive at the residue level. Mol Biol Evol.

[50] Scherrer MP, Meyer AG, Wilke CO (2012). Modeling coding-sequence evolution within the context of residue solvent accessibility. BMC Evol Biol.

[51] Wylie CS, Shakhnovich EI (2011). A biophysical protein folding model accounts for most mutational fitness effects in viruses. Proc Natl Acad Sci USA.

[52] Echave J, Spielman SJ, Wilke CO (2016). Causes of evolutionary rate variation among protein sites. Nat Rev Genet.

[53] Echave J, Jackson EL, Wilke CO (2015). Relationship between protein thermodynamic constraints and variation of evolutionary rates among sites. Phys Biol.

[54] Jimenez MJ, Arenas M, Bastolla U (2017). Substitution rates predicted by stability-constrained models of protein evolution are not consistent with empirical data. Mol Biol Evol.

[55] Lind PA, Arvidsson L, Berg OG, Andersson DI (2017). Variation in mutational robustness between different proteins and the predictability of fitness effects. Mol Biol Evol.

[56] Weile J, Sun S, Cote AG, Knapp J, Verby M, Mellor JC, Wu Y, Pons C, Wong C, van Lieshout N, Yang F, Tasan M, Tan G, Yang S, Fowler DM, Nussbaum R, Bloom JD, Vidal M, Hill DE, Aloy P, Roth FP (2017). A framework for exhaustively mapping functional missense variants. Mol Syst Biol.

[57] Jacquier H, Birgy A, Le Nagard H, Mechulam Y, Schmitt E, Glodt J, Bercot B, Petit E, Poulain J, Barnaud G, Gros PA, Tenaillon O (2013). Capturing the mutational landscape of the beta-lactamase TEM-1. Proc Natl Acad Sci USA.

[58] Henikoff S, Henikoff JG (1992). Amino acid substitution matrices from protein blocks. Proc Natl Acad Sci USA.

[59] Berezovsky IN, Zeldovich KB, Shakhnovich EI (2007). Positive and negative design in stability and thermal adaptation of natural proteins. PLoS Comput Biol.

[60] Kinjo AR, Nishikawa K (2004). Eigenvalue analysis of amino acid substitution matrices reveals a sharp transition of the mode of sequence conservation in proteins. Bioinformatics.

[61] Chiusano ML, Alvarez-Valin F, Di Giulio M, D’Onofrio G, Ammirato G, Colonna G, Bernardi G (2000). Second codon positions of genes and the secondary structures of proteins. Relationships and implications for the origin of the genetic code. Gene.

[62] Koonin EV, Novozhilov AS (2009). Origin and evolution of the genetic code: the universal enigma. IUBMB Life.

[63] Blazej P, Wnetrzak M, Mackiewicz D, Mackiewicz P (2018). Correction: Optimization of the standard genetic code according to three codon positions using an evolutionary algorithm. PLoS ONE.

[64] Drummond DA, Wilke CO (2008). Mistranslation-induced protein misfolding as a dominant constraint on coding-sequence evolution. Cell.

[65] Mohler K, Ibba M (2017). Translational fidelity and mistranslation in the cellular response to stress. Nat Microbiol.

[66] Mendez R, Fritsche M, Porto M, Bastolla U (2010). Mutation bias favors protein folding stability in the evolution of small populations. PLoS Comput Biol.

[67] D’Onofrio G, Mouchiroud D, Aissani B, Gautier C, Bernardi G (1991). Correlations between the compositional properties of human genes, codon usage, and amino acid composition of proteins. J Mol Evol.

[68] D’Onofrio G, Jabbari K, Musto H, Bernardi G (1999). The correlation of protein hydropathy with the base composition of coding sequences. Gene.

[69] Kudla G, Lipinski L, Caffin F, Helwak A, Zylicz M (2006). High guanine and cytosine content increases MRNA levels in mammalian cells. PLOS Biol.

[70] Chen SL, Lee W, Hottes AK, Shapiro L, McAdams HH (2004). Codon usage between genomes is constrained by genome-wide mutational processes. Proc Natl Acad Sci USA.

[71] Pucci F, Rooman M (2019). Relation between DNA ionization potentials, single base substitutions and pathogenic variants. BMC Genomics.

[72] de la Higuera I, Ferrer-Orta C, de Ávila AI, Perales C, Sierra M, Singh K, Sarafianos SG, Dehouck Y, Bastolla U, Verdaguer N, Domingo E (2015). Molecular and functional bases of selection against a mutation bias in an RNA virus. Genome Biol Evol.

[73] Kumar S (1996). Patterns of nucleotide substitution in mitochondrial protein coding genes of vertebrates. Genetics.

[74] Stoltzfus A, Norris RW (2016). On the causes of evolutionary transition:transversion bias. Mol Biol Evol.

[75] Lyons DM, Lauring AS (2017). Evidence for the selective basis of transition-to-transversion substitution bias in two rna viruses. Mol Biol Evol.

[76] Shah P, Gilchrist MA (2011). Explaining complex codon usage patterns with selection for translational efficiency, mutation bias, and genetic drift. Proc Natl Acad Sci USA.

[77] Coleman JR, Papamichail D, Skiena S, Futcher B, Wimmer E, Mueller S (2008). Virus attenuation by genome-scale changes in codon pair bias. Science.

[78] Tuller T, Waldman YY, Kupiec M, Ruppin E (2010). Translation efficiency is determined by both codon bias and folding energy. Proc Natl Acad Sci USA.

[79] Akashi H, Eyre-Walker A (1998). Translational selection and molecular evolution. Curr Opin Genet Dev.

[80] Archetti M (2009). Genetic robustness at the codon level as a measure of selection. Gene.

[81] Drummond DA, Wilke CO (2009). The evolutionary consequences of erroneous protein synthesis. Nat Rev Genet.

[82] Zhou T, Weems M, Wilke CO (2009). Translationally optimal codons associate with structurally sensitive sites in proteins. Mol Biol Evol.

[83] Lauring A, Acevedo A, Cooper S, Andino R (2012). Codon usage determines the mutational robustness, evolutionary capacity, and virulence of an RNA virus. Cell Host Microbe.

[84] Hildebrand F, Meyer A, Eyre-Walker A (2015). Evidence of selection upon genomic gc-content in bacteria,. PLoS Genet.

[85] Behura SK, Severson DW (2013). Codon usage bias: causative factors, quantification methods and genome-wide patterns: with emphasis on insect genomes. Biol Rev.

[86] Quax TF, Claassens N, Söll D, van der Oost J (2015). Codon bias as a means to fine-tune gene expression. Mol Cell.

[87] Hanson G, Coller J (2018). Codon optimality, bias and usage in translation and MRNA decay. Nat Rev Mol Cell Biol.

[88] LaBella AL, Opulente DA, Steenwyk JL, Hittinger CT, Rokas A (2019). Variation and selection on codon usage bias across an entire subphylum. PLoS Genet.

[89] Athey J, Alexaki A, Osipova E, Rostovtsev A, Santana-Quintero LV, Katneni U, Simonyan V, Kimchi-Sarfaty C (2017). A new and updated resource for codon usage tables. BMC Bioinforma.

[90] Fares MA (2015). The origins of mutational robustness. Trends Genet.

[91] Jarzab A, Kurzawa N, Hopf T, Moerch M, Zecha J, Leijten N, Bian Y, Musiol E, Maschberger M, Stoehr G, Becher I, Daly C, Samaras P, Mergner J, Spanier B, Angelov A, Werner T, Bantscheff M, Wilhelm M, Klingenspor M, Lemeer S, Liebl W, Hahne H, Savitski MM, Kuster B (2020). Meltome atlas-thermal proteome stability across the tree of life. Nat Methods.

[92] Engqvist MKM (2018). Correlating enzyme annotations with a large set of microbial growth temperatures reveals metabolic adaptations to growth at diverse temperatures. BMC Microbiol.

[93] Wang G, Dunbrack RL (2003). PISCES: a protein sequence culling server. Bioinformatics.

[94] Martin A (2005). Mapping PDB chains to UniProtKB entries. Bioinformatics. Bioinformatics.

[95] Kulikova T, Akhtar R, Aldebert P, Althorpe N, Andersson M, Baldwin A, Bates K, Bhattacharyya S, Bower L, Browne P, Castro M, Cochrane G, Duggan K, Eberhardt R, Faruque N, Hoad G, Kanz C, Lee C, Leinonen R, Lin Q, Lombard V, Lopez R, Lorenc D, McWilliam H, Mukherjee G, Nardone F, Pastor MP, Plaister S, Sobhany S, Stoehr P, Vaughan R, Wu D, Zhu W, Apweiler R (2007). EMBL nucleotide sequence database in 2006. Nucleic Acids Res.

[96] Thompson JD, Higgins DG, Gibson TJ (1994). CLUSTAL W: improving the sensitivity of progressive multiple sequence alignment through sequence weighting, position-specific gap penalties and weight matrix choice. Nucleic Acids Res.

[97] Schwersensky M, Rooman M, Pucci F. Analyzing large-scale predictions of stability changes upon mutations. https://github.com/3BioCompBio/LargeScaleMutagenesis. Accessed 7 July 2020.

[98] Bartlett J, Holloway E (2019). Generalized information: a straightforward method for judging machine learning models. Commun Blyth Inst.

[99] Dehouck Y, Grosfils A, Folch B, Gilis D, Bogaerts P, Rooman M (2009). Fast and accurate predictions of protein stability changes upon mutations using statistical potentials and neural networks: PoPMuSiC-2.0. Bioinformatics.

