# Supplementary material for: Large-scale in silico mutagenesis experiments reveal optimization of genetic code and codon usage for protein mutational robustness
Source: BMC Biol. 2020 Oct 20;18:146. doi: 10.1186/s12915-020-00870-9 (PMC7576759; doi:10.1186/s12915-020-00870-9)
Supplement: Supplementary file 1 — Additional file 1 Large-scale in-silico mutagenesis experiments reveal optimization of genetic code and codon usage for protein mutational robustness [file 12915_2020_870_MOESM1_ESM.pdf]

Supplementary Information

Large-scale *in-silico* mutagenesis experiments reveal optimization of genetic code and codon usage for protein mutational robustness

Martin Schwersensky[1,2,†], Marianne Rooman[1,2,*,†], and Fabrizio Pucci[1,2,†]

[1]Computational Biology and Bioinformatics, Université Libre de Bruxelles, CP 165/61, Roosevelt Ave. 50, 1050 Brussels, Belgium
[2] Interuniversity Institute of Bioinformatics in Brussels, Boulevard du Triomphe, 1050 Brussels, Belgium
[†] Contributed equally to this work

Table of Content

- Table S1. Amino acid mutations in $\mathcal{M}_{PoP}$ due to single nucleobase substitutions (μSBS) and due to multiple nucleobase substitutions that cannot be obtained through single base substitutions (μMBS).
- Table S2. Amino acid mutations in $\mathcal{M}_{Exp}$ due to single nucleobase substitutions (μSBS) and due to multiple nucleobase substitutions that cannot be obtained through single base substitutions (μMBS).
- Table S3. Amino acid mutations in $\mathcal{M}_{PoP}$ due to substitutions of a single nucleobase Gua, Cyt, Ade or Thy.
- Table S4. Amino acid mutations in $\mathcal{M}_{Exp}$ due to substitutions of a single nucleobase Gua, Cyt, Ade or Thy.
- Table S5. Amino acid mutations in $\mathcal{M}_{PoP}$ due to single nucleobase substitutions (μSBS) that correspond to transitions or transversions.
- Table S6. Amino acid mutations in $\mathcal{M}_{Exp}$ due to single nucleobase substitutions (μSBS) that correspond to transitions or transversions.
- Table S7. Amino acid mutations in $\mathcal{M}_{PoP}$ due to single nucleobase substitutions (μSBS) and due to multiple nucleobase substitutions that cannot be obtained through single base substitutions (μMBS).
- Table S8. Amino acid mutations in $\mathcal{M}_{Exp}$ due to single nucleobase substitutions (μSBS) and due to multiple nucleobase substitutions that cannot be obtained through single base substitutions (μMBS).
- Table S9. Difference between $\langle \Delta\Delta G \rangle$ for μSBSs in $\mathcal{M}_{PoP}$ reached from synonymous codons (syn) or from the wild-type codon (used), according to whether the position-dependent frequency of translation errors is taken into account (translation) or not (random).
- Figure S1. Standard genetic code.
- Figure S2. Influence of the protein length on the mutational robustness for core residues (RSA < 20%).

- Figure S3. Hydrophobic residue content (Val, Ile, Leu, Phe) in the protein core (RSA≤20%) as a function of the protein length.
- Figure S4. Difference between the mean of the experimental $\Delta\Delta G$ values per RSA bin of long proteins (L >200 residues) and short proteins (L ≤200 residues) as a function of RSA.
- Figure S5. $\Delta\Delta G$ (in kcal/mol) distribution for different RSA ranges, and for different types of single and multiple base substitutions (μSBS and μMBS).
- Figure S6. Ratio of stabilizing, destabilizing and neutral μSBSs considering random mutations (that occur with equal frequency at each codon position)
- Figure S7. Average fitness score of mutations from (Weile et al., 2017) (a) and (Lind et al., 2017) (b)
- Figure S8. Average $\Delta\Delta G$ (in kcal/mol) of μSBSs per residue as a function of the position in the sequence of wheat agglutinin isolectin 3 (PDB code 2X52, chain A).
- Figure S9. Schematic picture of the computational pipeline used in this paper.
- Table S10. List of eukaryote organisms in the dataset $\mathcal{D}$, with their number of proteins and average $\Delta\Delta G$ upon all possible point mutations.
- Table S11. List of bacterial organisms in the dataset $\mathcal{D}$, with their number of proteins and average $\Delta\Delta G$ upon all possible point mutations.
- Table S12. List of archaea organisms in the dataset $\mathcal{D}$, with their number of proteins and average $\Delta\Delta G$ upon all possible point mutations.
- Table S13. List of virus organisms in the dataset $\mathcal{D}$, with their number of proteins and average $\Delta\Delta G$ upon all possible point mutations.

Table S1. Amino acid mutations in $\mathcal{M}_{PoP}$ due to single nucleobase substitutions (μSBS) and due to multiple nucleobase substitutions that cannot be obtained through single base substitutions (μMBS). In $\langle\Delta\Delta G_{(d,s)}\rangle$, the synonymous mutations (with $\Delta\Delta G = 0$) are included in the mean and the degeneracy (the number of different base substitutions yielding the same amino acid mutation) is taken into account. The percentages of mutations refer to $\langle\Delta\Delta G\rangle$, without degeneracy and synonymous mutations.

RSA %	Percentage %	$\langle\Delta\Delta G\rangle$ kcal/mol	$\langle\Delta\Delta G_{(d,s)}\rangle$ kcal/mol	Stabilizing %	Neutral %	Destabilizing %
			μSBSs			
$[0-5]$	32.08	1.22	0.81	4.01	24.60	71.39
$]5-10]$	8.62	0.96	0.67	3.58	31.63	64.79
$]10-20]$	12.57	0.83	0.61	3.62	36.29	60.08
$]20-50]$	28.32	0.62	0.48	2.94	47.94	49.12
$]50-100]$	18.41	0.29	0.23	5.65	66.26	28.09
			μMBSs			
$[0-5]$	31.23	1.54	1.63	3.81	14.26	81.92
$]5-10]$	8.56	1.18	1.24	3.80	21.11	75.09
$]10-20]$	12.47	1.00	1.06	3.95	25.98	70.07
$]20-50]$	28.17	0.74	0.78	3.19	37.63	59.18
$]50-100]$	19.57	0.30	0.35	7.15	61.54	31.31
			base I μSBSs			
$[0-5]$	33.17	0.85	0.78	3.84	36.06	60.10
$]5-10]$	8.81	0.81	0.75	2.74	39.34	57.92
$]10-20]$	12.69	0.76	0.72	2.55	42.25	55.20
$]20-50]$	27.56	0.61	0.61	2.03	51.83	46.14
$]50-100]$	17.77	0.26	0.29	4.95	70.34	24.71
			base II μSBSs			
$[0-5]$	32.01	1.50	1.51	4.26	15.88	79.86
$]5-10]$	8.61	1.07	1.12	4.19	25.77	70.05
$]10-20]$	12.61	0.89	0.95	0.83	31.97	63.67
$]20-50]$	28.62	0.64	0.71	3.45	45.46	51.10
$]50-100]$	18.15	0.31	0.37	6.04	63.40	30.55
			base III μSBSs			
$[0-5]$	28.16	0.85	0.15	3.77	31.23	65.00
$]5-10]$	8.22	0.76	0.16	3.19	35.66	61.15
$]10-20]$	12.25	0.69	0.15	3.24	38.36	58.41
$]20-50]$	29.94	0.49	0.13	2.75	49.59	47.66
$]50-100]$	21.44	0.15	0.04	5.78	73.99	20.23
			bases I+III μMBSs			
$[0-5]$	15.41	1.00	1.03	7.55	24.73	67.72
$]5-10]$	6.69	0.84	0.84	7.60	29.76	62.64
$]10-20]$	11.62	0.68	0.68	7.47	34.99	57.55
$]20-50]$	35.73	0.43	0.43	5.00	49.52	45.49
$]50-100]$	30.55	0.14	0.14	7.57	70.11	22.31
			bases I+II and II+III μMBSs			
$[0-5]$	33.16	1.57	1.59	3.50	13.75	82.75
$]5-10]$	8.75	1.21	1.22	3.39	20.36	76.25
$]10-20]$	12.55	1.05	1.05	3.49	24.98	71.54
$]20-50]$	27.33	0.79	0.77	2.96	36.03	61.00
$]50-100]$	18.21	0.35	0.35	6.68	59.58	33.74
			bases I+II+III μMBSs			
$[0-5]$	27.36	1.52	1.62	5.57	14.78	79.65
$[5-10]$	8.51	1.08	1.09	5.07	22.27	72.67
$]10-20]$	12.47	0.87	0.85	5.46	27.89	66.65
$]20-50]$	29.22	0.65	0.63	3.10	38.45	58.44
$]50-100]$	22.43	0.13	0.11	10.43	66.07	23.51

Table S2. Amino acid mutations in $\mathcal{M}_{Exp}$ due to single nucleobase substitutions (μSBS) and due to multiple nucleobase substitutions that cannot be obtained through single base substitutions (μMBS). When the number of occurrences is too low to yield reliable statistics (< 100), the RSA intervals are combined, and if the number is still too low, no results are given. We did not indicate the percentage of stabilizing, neutral and destabilizing mutations as the mutations are here non-random.

RSA %	Number	$\langle \Delta\Delta G \rangle$ kcal/mol
μSBSs		
[0 − 20]	774	1.23
]20 − 50]	434	0.73
]50 − 100]	286	0.30
μMBSs		
[0 − 20]	544	1.73
]20 − 50]	349	0.88
]50 − 100]	261	0.33
base I μSBSs		
[0 − 20]	387	1.06
]20 − 50]	195	0.68
]50 − 100]	121	0.19
base II μSBSs		
[0 − 20]	392	1.44
]20 − 50]	232	0.78
]50 − 100]	152	0.43
base III μSBSs		
[0 − 100]	104	0.79
bases I+III μMBSs		
[0 − 100]	< 100	-
bases I+II and II+III μMBSs		
[0 − 20]	503	1.80
]20 − 50]	316	0.94
]50 − 100]	216	0.37
bases I+II+III μMBSs		
[0 − 100]	< 100	-

Table S3. Amino acid mutations in $\mathcal{M}_{PoP}$ due to substitutions of a single nucleobase Gua, Cyt, Ade or Thy. In $\langle\Delta\Delta G_{(d)}\rangle$, the degeneracy (the number of different base substitutions yielding the same amino acid mutation) is taken into account, and in $\langle\Delta\Delta G_{(d,s)}\rangle$, the synonymous mutations (with $\Delta\Delta G = 0$ are also considered. The percentages of mutations refer to $\langle\Delta\Delta G_{(d)}\rangle$, without synonymous mutations.

RSA %	Percentage %	$\langle\Delta\Delta G_{(d)}\rangle$ kcal/mol	$\langle\Delta\Delta G_{(d,s)}\rangle$ kcal/mol	Stabilizing %	Neutral %	Destabilizing %
Ade-μSBSs						
$[0-5]$	18.41	0.55	0.42	8.64	42.40	48.95
$]5-10]$	23.47	0.59	0.48	6.65	45.58	47.77
$]10-20]$	26.82	0.56	0.47	5.78	47.39	6.83
$]20-50]$	34.99	0.47	0.40	3.25	54.85	41.90
$]50-100]$	40.52	0.21	0.18	5.85	70.42	23.74
Cyt-μSBSs						
$[0-5]$	21.11	0.82	0.52	4.61	31.77	63.62
$]5-10]$	21.80	0.79	0.52	2.66	34.31	63.02
$]10-20]$	22.11	0.77	0.52	2.26	36.08	61.65
$]20-50]$	21.98	0.65	0.46	2.08	42.36	55.56
$]50-100]$	20.29	0.30	0.22	4.23	62.97	32.80
Gua-μSBSs						
$[0-5]$	26.02	0.99	0.75	6.34	31.51	62.14
$]5-10]$	824.85	0.91	0.70	4.95	36.54	58.51
$]10-20]$	25.61	0.84	0.65	4.70	40.22	55.08
$]20-50]$	27.27.65	0.68	0.54	3.40	50.03	46.57
$]50-100]$	29.98	0.36	0.29	5.81	64.93	29.26
Thy-μSBSs						
$[0-5]$	34.46	1.69	1.33	0.62	13.86	85.52
$]5-10]$	29.88	1.26	0.98	1.19	20.99	77.82
$]10-20]$	25.47	1.06	0.81	1.57	25.88	72.56
$]20-50]$	15.38	0.62	0.43	3.01	45.35	51.64
$]50-100]$	9.21	0.21	0.13	5.75	69.82	24.43

Table S4. Amino acid mutations in $\mathcal{M}_{Exp}$ due to substitutions of a single nucleobase Gua, Cyt, Ade or Thy. In $\langle\Delta\Delta G_{(d)}\rangle$, the degeneracy (the number of different base substitutions yielding the same amino acid mutation) is taken into account. When the number of occurrences is too low to yield reliable statistics (< 100), the RSA intervals are combined.

RSA %	Number	$\langle\Delta\Delta G_{(d)}\rangle$ kcal/mol
Ade-μSBSs		
$[0-20]$	225	0.89
$]20-50]$	150	0.59
$]50-100]$	114	0.31
Cyt-μSBSs		
$[0-20]$	127	1.16
$]20-100]$	105	0.44
Gua-μSBSs		
$[0-20]$	195	0.87
$]20-100]$	202	0.58
Thy-μSBSs		
$[0-20]$	197	2.08
$]20-100]$	115	0.80

Table S5. Amino acid mutations in $\mathcal{M}_{PoP}$ due to single nucleobase substitutions (μSBS) that correspond to transitions or transversions. In $\langle \Delta\Delta G_{(d)} \rangle$, the degeneracy (the number of different base substitutions yielding the same amino acid mutation) is taken into account, and in $\langle \Delta\Delta G_{(d,s)} \rangle$, the synonymous mutations (with $\Delta\Delta G = 0$) are also included. The percentages of mutations refer to $\langle \Delta\Delta G_{(d,s)} \rangle$.

RSA %	Percentage %	$\langle \Delta\Delta G_{(d)} \rangle$ kcal/mol	$\langle \Delta\Delta G_{(d,s)} \rangle$
Transition μSBSs			
$[0-5]$	31.84	1.17	0.75
$]5-10]$	8.56	0.97	0.63
$]10-20]$	12.47	0.85	0.55
$]20-50]$	28.19	0.61	0.40
$]50-100]$	18.94	0.31	0.20
Transversion μSBSs			
$[0-5]$	32.05	1.09	0.86
$]5-10]$	8.62	0.89	0.72
$]10-20]$	12.51	0.79	0.64
$]20-50]$	28.07	0.59	0.49
$]50-100]$	18.75	0.26	0.22

Table S6. Amino acid mutations in $\mathcal{M}_{Exp}$ due to single nucleobase substitutions (μSBS) that correspond to transitions or transversions. In $\langle \Delta\Delta G_{(d)} \rangle$, the degeneracy (the number of different base substitutions yielding the same amino acid mutation) is taken into account.

RSA %	Number	$\langle \Delta\Delta G_{(d)} \rangle$ kcal/mol
Transition μSBSs		
$[0-20]$	296	1.35
$]20-100]$	249	0.68
Transversion μSBSs		
$[0-20]$	448	1.18
$]20-100]$	437	0.47

Table S7. Amino acid mutations in $\mathcal{M}_{PoP}$ due to single nucleobase substitutions (μSBS) and due to multiple nucleobase substitutions that cannot be obtained through single base substitutions (μMBS). In $\langle \Delta\Delta G_{(d)} \rangle$, the degeneracy (the number of different base substitutions yielding the same amino acid mutation) is taken into account. $\langle \Delta\Delta G^{used} \rangle$ and $\langle \Delta\Delta G^{syn} \rangle$ refer to mean $\Delta\Delta G$ values of mutations that are reached from the wild-type codon or a synonymous codon, respectively. σ is the standard deviation of the $\Delta\Delta G$ distribution: $\sigma^2(\Delta\Delta G) = \sigma^2(\Delta\Delta G^{used}) + \sigma^2(\Delta\Delta G^{syn})$. The percentages of mutations refer to those starting from the wild-type codon.

RSA %	Percentage %	$\frac{\langle \Delta\Delta G^{syn}_{(d)} \rangle - \langle \Delta\Delta G^{used}_{(d)} \rangle}{\langle \Delta\Delta G^{used}_{(d)} \rangle}$ %	$\frac{\langle \Delta\Delta G^{syn}_{(d)} \rangle - \langle \Delta\Delta G^{used}_{(d)} \rangle}{\sigma}$ %	Stabilizing %	Neutral %	Destabilizing %
μSBSs						
[0 – 5]	30.97	4.01	2.83	4.43	27.49	68.08
]5 – 10]	8.53	4.08	2.78	3.73	33.53	62.74
]10 – 20]	12.48	6.62	4.26	3.65	37.58	58.77
]20 – 50]	28.49	15.52	8.52	3.00	49.31	47.69
]50 – 100]	19.53	24.75	7.61	5.50	67.21	27.29
μMBSs						
[0 – 5]	31.19	4.38	4.13	3.79	14.55	81.66
]5 – 10]	8.55	4.99	4.15	3.82	21.30	74.89
]10 – 20]	12.51	6.06	4.60	4.04	26.29	69.67
]20 – 50]	28.44	9.34	5.96	3.34	38.88	57.78
]50 – 100]	19.30	19.92	6.34	6.91	61.96	31.12
base I μSBSs						
[0 – 5]	32.94	-5.85	-3.80	3.59	35.91	60.51
]5 – 10]	8.83	-2.69	-1.76	2.77	39.35	57.89
]10 – 20]	12.70	2.55	1.61	2.54	42.52	54.94
]20 – 50]	27.50	18.48	10.03	2.13	53.95	43.92
]50 – 100]	18.03	35.48	10.40	4.52	71.94	23.54
base II μSBSs						
[0 – 5]	31.35	6.85	5.78	44.78	17.63	77.59
]5 – 10]	8.53	6.53	4.83	4.50	26.41	69.09
]10 – 20]	12.48	6.91	4.66	4.56	31.75	63.69
]20 – 50]	28.43	9.39	5.38	3.69	44.06	52.26
]50 – 100]	19.21	12.84	4.55	6.18	60.34	33.48
base III μSBSs						
[0 – 5]	24.07	15.79	10.24	6.33	34.14	59.53
]5 – 10]	7.67	8.47	5.64	4.27	38.80	56.93
]10 – 20]	11.87	11.73	7.48	4.14	41.35	54.51
]20 – 50]	31.51	18.28	10.20	3.25	52.36	44.39
]50 – 100]	24.88	6.10	1.46	5.93	73.79	20.28
bases I+III μMBSs						
[0 – 5]	22.99	-11.65	-7.18	5.98	35.87	58.15
]5 – 10]	7.47	-6.32	-3.98	5.61	36.21	58.19
]10 – 20]	12.00	-0.75	-0.44	5.84	39.08	55.08
]20 – 50]	32.49	17.89	9.36	4.22	48.28	47.50
]50 – 100]	25.05	30.78	7.15	7.60	67.90	24.50
bases I+II and II+III μMBSs						
[0 – 5]	32.42	2.06	2.00	3.50	13.82	82.68
]5 – 10]	8.72	2.31	1.94	3.60	21.67	74.73
]10 – 20]	12.63	2.54	1.93	3.82	26.98	69.21
]20 – 50]	27.98	4.78	3.07	3.21	39.85	56.94
]50 – 100]	18.25	9.59	3.18	6.48	62.85	30.67
bases I+II+III μMBSs						
[0 – 5]	33.73	-0.45	-0.44	3.52	12.77	83.71
]5 – 10]	8.68	0.63	0.55	3.68	20.79	75.53
]10 – 20]	12.41	1.83	1.41	4.01	26.62	69.37
]20 – 50]	27.32	2.83	1.93	3.00	38.05	58.95
]50 – 100]	17.86	16.46	5.29	7.46	61.90	30.64

Table S8. Amino acid mutations in $\mathcal{M}_{Exp}$ due to single nucleobase substitutions (μSBS) and due to multiple nucleobase substitutions that cannot be obtained through single base substitutions (μMBS). In $\langle \Delta\Delta G_{(d)} \rangle$, the degeneracy (the number of different base substitutions yielding the same amino acid mutation) is taken into account. $\langle \Delta\Delta G^{used} \rangle$ and $\langle \Delta\Delta G^{syn} \rangle$ refer to mean $\Delta\Delta G$ values of mutations that are reached from the wild-type codon or a synonymous codon, respectively. σ is the standard deviation of the $\Delta\Delta G$ distribution: $\sigma^2(\Delta\Delta G) = \sigma^2(\Delta\Delta G^{used}) + \sigma^2(\Delta\Delta G^{syn})$. "Number" refers to the number of mutations starting from the wild-type codon; when it is too low to yield reliable statistics (< 100), the RSA intervals are combined.

RSA %	Number	$\frac{\langle \Delta\Delta G^{syn}_{(d)} \rangle - \langle \Delta\Delta G^{used}_{(d)} \rangle}{\langle \Delta\Delta G^{used}_{(d)} \rangle}$ %	$\frac{\langle \Delta\Delta G^{syn}_{(d)} \rangle - \langle \Delta\Delta G^{used}_{(d)} \rangle}{\sigma}$ %
μSBSs			
$[0-20]$	744	0.67	0.40
$]20-50]$	402	11.03	5.01
$]50-100]$	284	28.42	5.56
μMBSs			
$[0-20]$	1981	10.28	7.32
$]20-50]$	1144	17.14	8.27
$]50-100]$	885	37.11	9.23
base I μSBSs			
$[0-20]$	339	-8.58	-5.38
$]20-50]$	174	13.87	6.74
$]50-100]$	115	38.24	5.67
base II μSBSs			
$[0-20]$	328	8.42	5.15
$]20-50]$	183	7.11	3.30
$]50-100]$	129	14.60	4.03
base III μSBSs			
$[0-100]$	162	2.97	1.05
bases I+III μMBSs			
$[0-100]$	183	44.67	13.08
bases I+II and II+III μMBSs			
$[0-20]$	550	1.48	1.06
$[20-50]$	304	2.01	0.97
$[50-100]$	231	14.36	3.71
bases I+II+III μMBSs			
$[0-100]$	259	36.08	12.60

Table S9. Difference between $\langle\Delta\Delta G\rangle$ for μSBSs in $\mathcal{M}_{PoP}$ reached from synonymous codons (syn) or from the wild-type codon (used), according to whether the position-dependent frequency of translation errors is taken into account (translation) or not (random). σ is the standard deviation of the $\Delta\Delta G$ distribution. Here the biased and unbiased codons, differently from Table 8 of the main text, are not defined in terms of the deviation from the codon equiprobability but in terms of the deviation with respect the expected frequency under the observed base frequencies.

$(\langle\Delta\Delta G^{syn}\rangle - \langle\Delta\Delta G^{used}\rangle)/\sigma$		
	Biased	Unbiased
Random	8%	4%
Translation	9%	2%

Codon Position II

Codon Position I (rows) / Codon Position III (right column)

Codon Position I	T		C		A		G		Codon Position III
T	TTT	Phe	TCT	Ser	TAT	Tyr	TGT	Cys	T
	TTC		TCC		TAC		TGC		C
	TTA	Leu	TCA		TAA	Stop	TGA	Stop	A
	TTG		TCG		TAG		TGG	Trp	G
C	CTT	Leu	CCT	Pro	CAT	His	CGT	Arg	T
	CTC		CCC		CAC		CGC		C
	CTA		CCA		CAA	Gln	CGA		A
	CTG		CCG		CAG		CGG		G
A	ATT	Ile	ACT	Thr	AAT	Asn	AGT	Ser	T
	ATC		ACC		AAC		AGC		C
	ATA		ACA		AAA	Lys	AGA	Arg	A
	ATG	Met	ACG		AAG		AGG		G
G	GTT	Val	GCT	Ala	GAT	Asp	GGT	Gly	T
	GTC		GCC		GAC		GGC		C
	GTA		GCA		GAA	Glu	GGA		A
	GTG		GCG		GAG		GGG		G

Figure S1. Standard genetic code

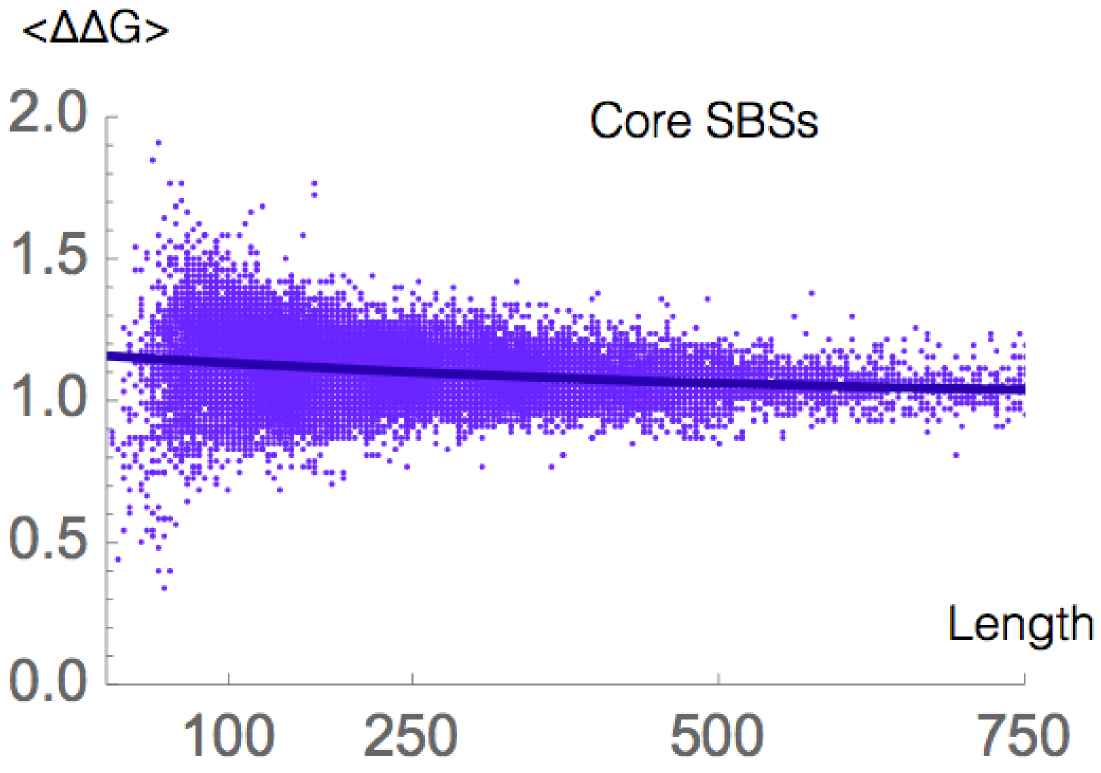

Figure S2. Influence of the protein length on the mutational robustness for core residues (RSA $<$ 20%).

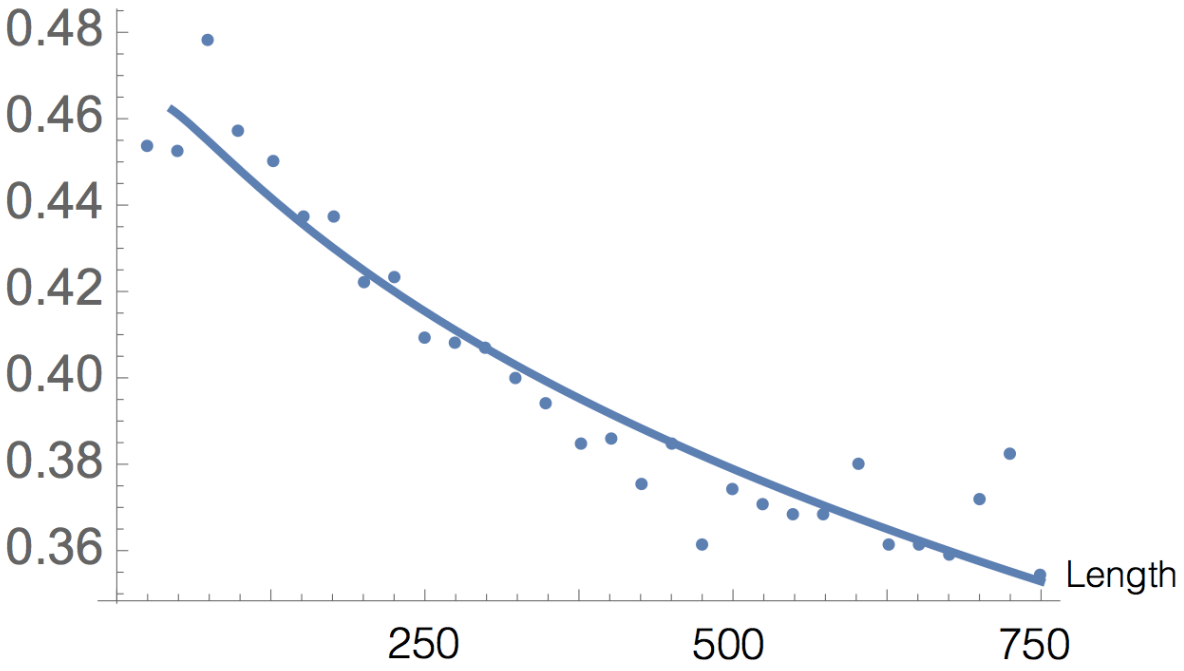

Figure S3. Hydrophobic residue content (Val, Ile, Leu, Phe) in the protein core (RSA$\leq$20%) as a function of the protein length.

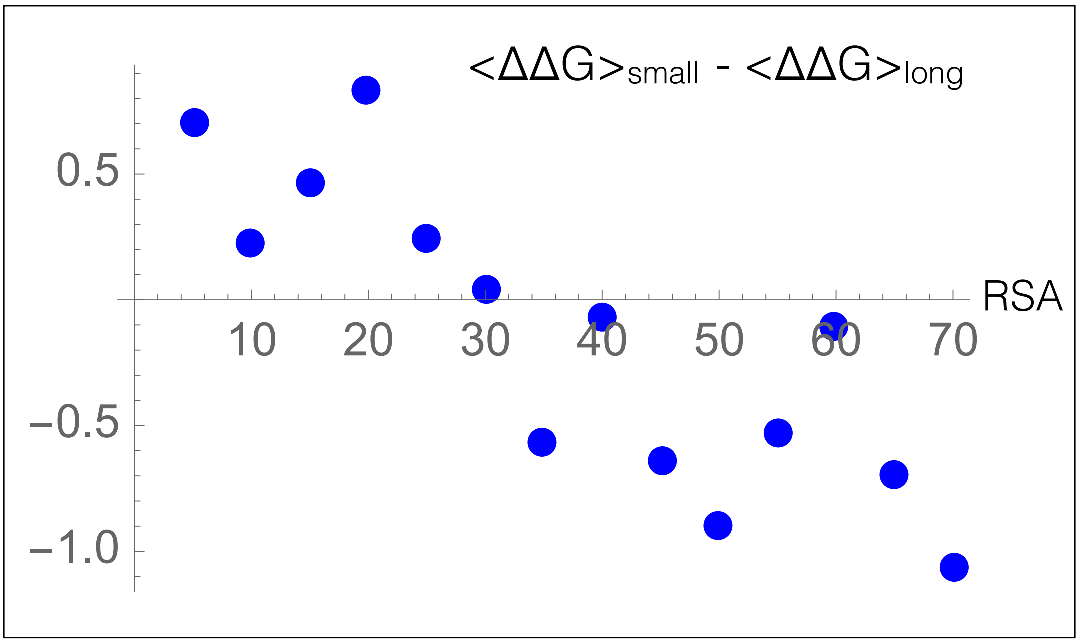

Figure S4. Difference between the mean of the experimental $\Delta\Delta G$ values per RSA bin of long proteins (L >200 residues) and short proteins ($L \leq$200 residues) as a function of RSA.

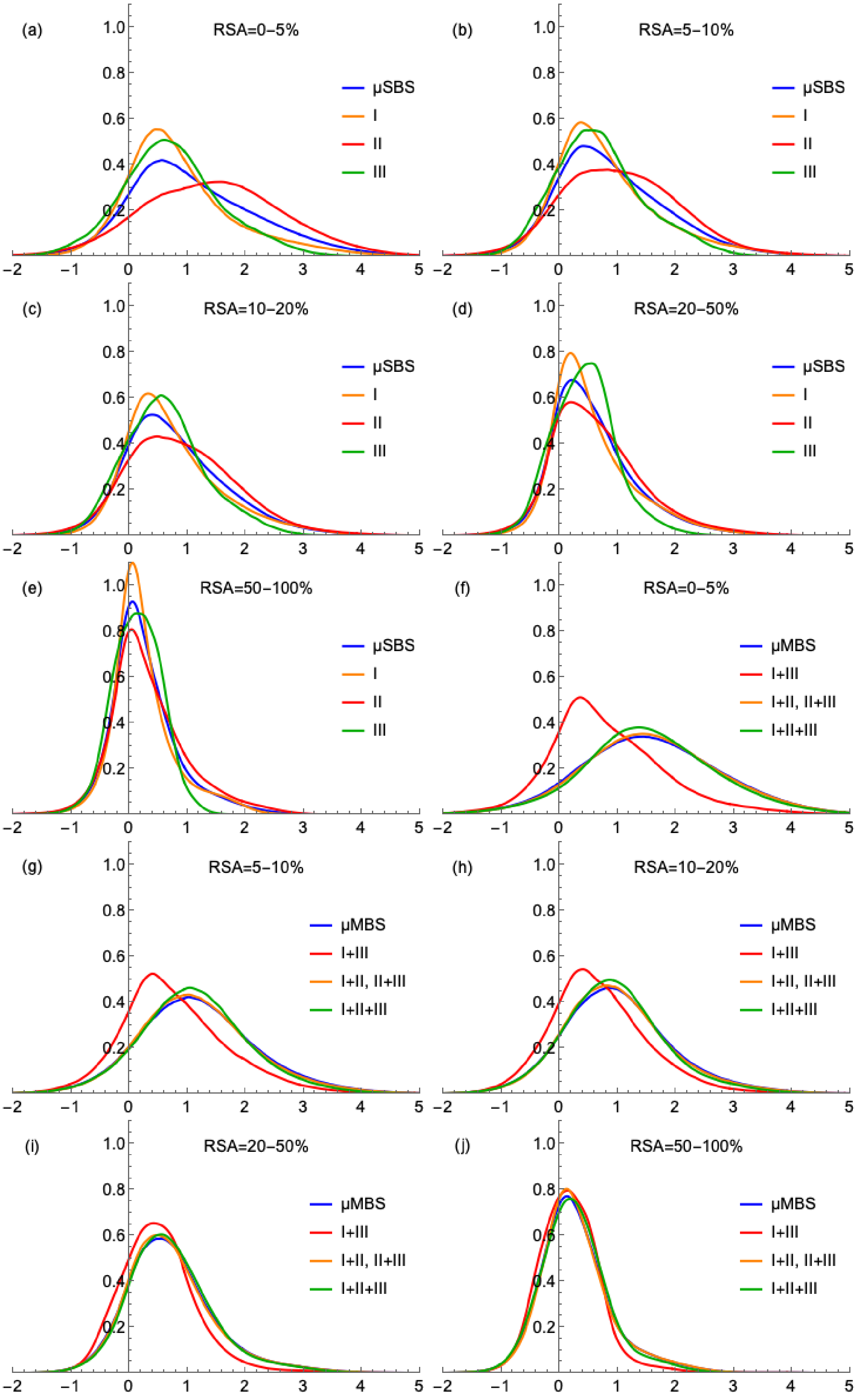

Figure S5. $\Delta\Delta G$ (in kcal/mol) distribution for different RSA ranges, and for different types of single and multiple base substitutions (μSBS and μMBS). I, II, III refer to the position of the substituted base in the codon. The degeneracy, *i.e.* the number of base substitutions leading to the same amino acid mutation, is taken into account

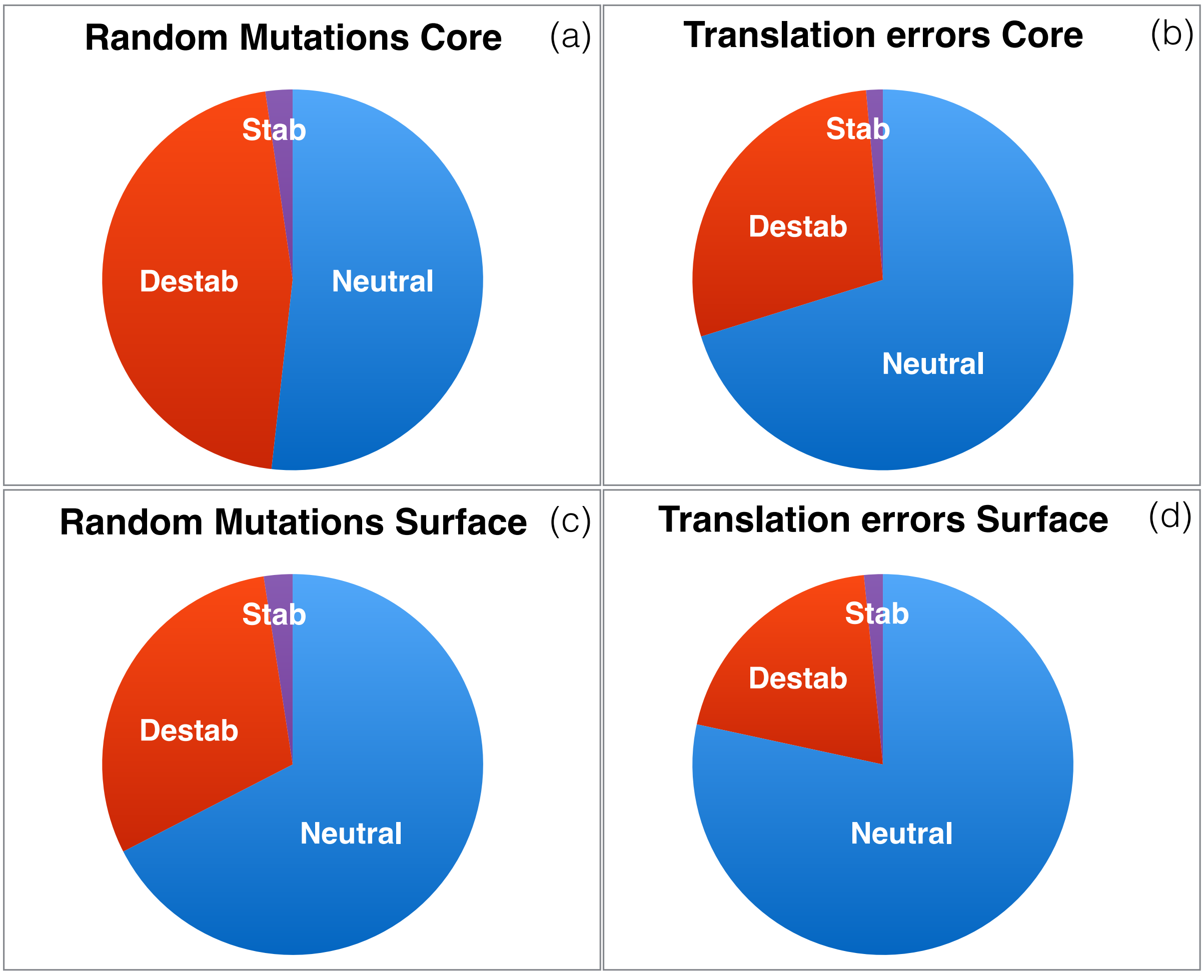

Figure S6. Ratio of stabilizing, destabilizing and neutral μSBSs considering random mutations (that occur with equal frequency at each codon position) inserted in the core (RSA$\leq$ 20%) (a) and at the surface (RSA$>$ 20%) (c) or translation errors (that occur with different frequency at each codon position) (f) inserted in the core (b) and at the surface (d).

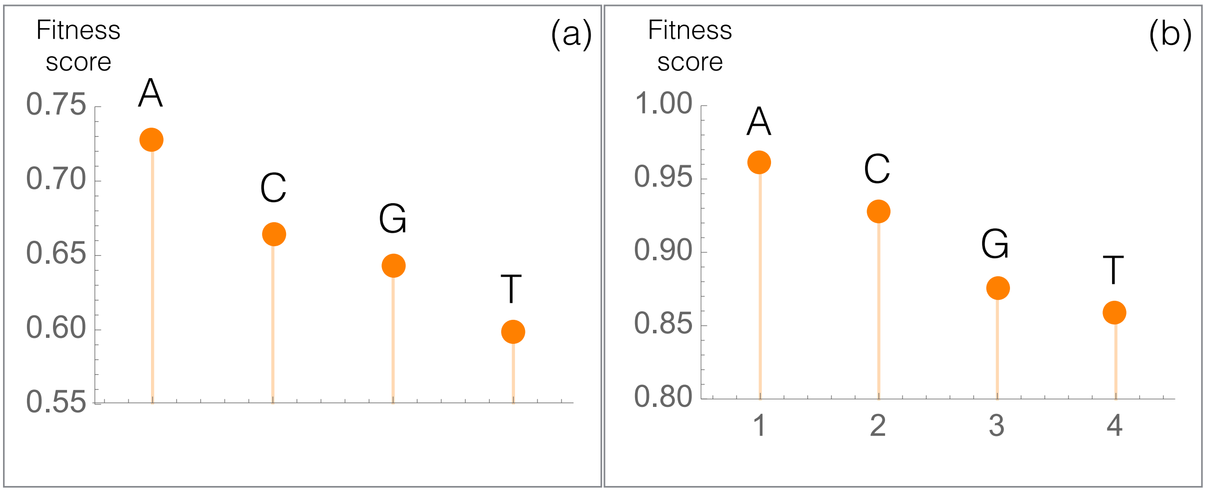

Figure S7. Average fitness score of mutations from (Weile et al., 2017) (a) and (Lind et al., 2017) (b).

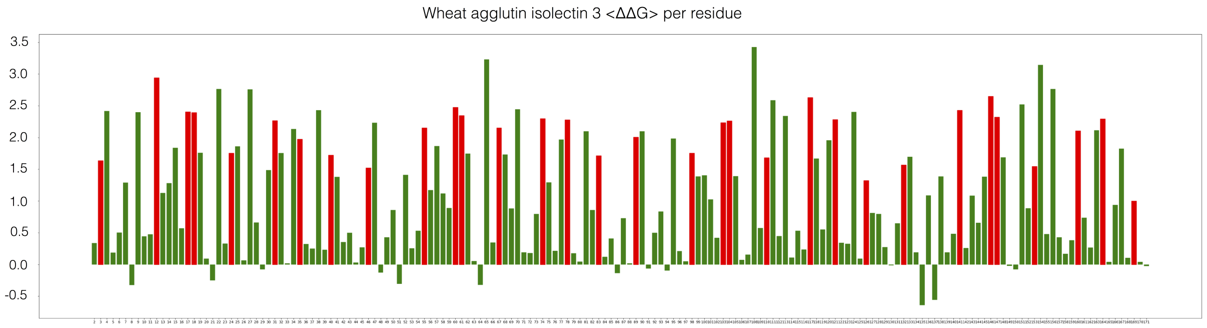

Figure S8. Average $\Delta\Delta G$ (in kcal/mol) of μSBSs per residue as a function of the position in the sequence of wheat agglutinin isolectin 3 (PDB code 2X52, chain A). The residues involved in disulfide bridges are in red and the other in green.

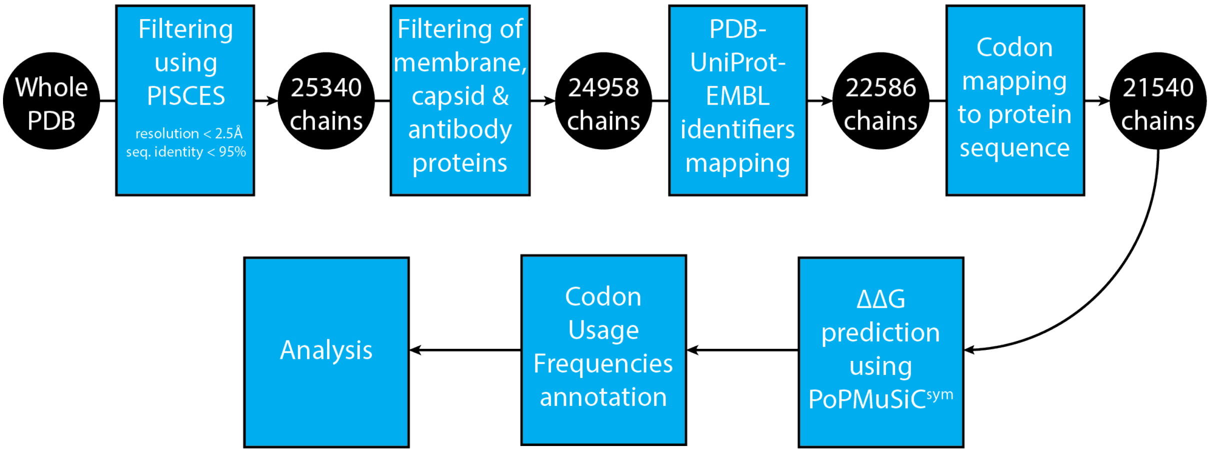

Figure S9. Schematic picture of the computational pipeline used in this paper.

Table S10. List of eukaryote organisms in the dataset $\mathcal{D}$, with their number of proteins and average $\Delta\Delta G$ upon all possible point mutations

Organism	Number of proteins	$\langle \Delta\Delta G \rangle$ (kcal/mol)
homo_sapiens	3495	0.867
saccharomyces_cerevisiae	692	0.869
mus_musculus	682	0.866
rattus_norvegicus	277	0.886
arabidopsis_thaliana	269	0.886
drosophila_melanogaster	175	0.848
bos_taurus	170	0.901
plasmodium_falciparum	121	0.870
schizosaccharomyces_pombe	88	0.858
caenorhabditis_elegans	88	0.868
trypanosoma_brucei	85	0.905
gallus_gallus	68	0.885
sus_scrofa	54	0.930
leishmania_major	50	0.890
trypanosoma_cruzi	43	0.933
danio_rerio	40	0.842
toxoplasma_gondii	40	0.908
oryctolagus_cuniculus	39	0.890
oryza_sativa	37	0.923
entamoeba_histolytica	36	0.923
cryptosporidium_parvum	32	0.885
xenopus_laevis	28	0.862
plasmodium_vivax	28	0.878
neosartorya_fumigata	27	0.930
candida_albicans	26	0.901
chlamydomonas_reinhardtii	26	0.908
hordeum_vulgare	25	0.931
zea_mays	25	0.919
bombyx_mori	22	0.865
aspergillus_niger	21	0.973
glycine_max	20	0.913
hypocrea_jecorina	20	0.966
schistosoma_mansoni	20	0.920
dictyostelium_discoideum	19	0.860
encephalitozoon_cuniculi	18	0.878
chaetomium_thermophilum	18	0.887
phanerochaete_chrysosporium	17	0.938
spinacia_oleracea	17	0.948
nicotiana_tabacum	17	0.907
pisum_sativum	16	0.945
solanum_lycopersicum	16	0.952
giardia_intestinalis	15	0.918
solanum_tuberosum	15	0.852
anopheles_gambiae	15	0.879
neurospora_crassa	15	0.898
aspergillus_oryzae	13	0.938
emericella_nidulans	13	0.910
triticum_aestivum	13	0.981
medicago_truncatula	12	0.889
rattus_rattus	12	0.871
plasmodium_yoelii	12	0.904
aedes_aegypti	11	0.873
kluyveromyces_lactis	11	0.885
equus_caballus	11	0.934
candida_glabrata	10	0.864
ovis_aries	10	0.904
trichomonas_vaginalis	9	0.911
vitis_vinifera	9	0.926
canis_lupus	9	0.858
acanthamoeba_polyphaga	9	0.840
medicago_sativa	9	0.949
leishmania_mexicana	9	0.980
naja_atra	9	0.791
leishmania_donovani	8	0.886
aequorea_victoria	8	0.829
ascaris_suum	8	0.821
deinagkistrodon_acutus	7	0.879
xenopus_tropicalis	7	0.816
physarum_polycephalum	7	0.900
cricetulus_griseus	7	0.894
coccidioides_immitis	7	0.954
aspergillus_aculeatus	7	1.002
cryptococcus_neoformans	7	0.924
coprinopsis_cinerea	7	0.940
phaseolus_vulgaris	7	0.973
naja_sagittifera	6	0.848
lupinus_luteus	6	0.905
sorghum_bicolor	6	0.948
plasmodium_knowlesi	6	0.845
arachis_hypogaea	6	0.988
ricinus_communis	6	0.880
fasciola_hepatica	6	0.858
canavalia_ensiformis	6	1.004
podospora_anserina	6	0.895
candida_tropicalis	6	0.877
physeter_catodon	5	0.833
necator_americanus	5	0.875
protobothrops_flavoviridis	5	0.849
tenebrio_molitor	5	0.963
physcomitrella_patens	5	0.887
phleum_pratense	5	0.904
manduca_sexta	5	0.847
lithobates_catesbeiana	5	0.867
aspergillus_terreus	5	0.921
lama_glama	5	0.803
populus_tremula	5	0.875
leishmania_infantum	5	0.901

candida_parapsilosis	5	0.939
hevea_brasiliensis	5	0.990
carica_papaya	5	0.970
moniliophthora_perniciosa	5	0.949
babesia_bovis	5	0.931
musca_domestica	5	0.819
apis_mellifera	5	0.856
rauvolfia_serpentina	5	0.945
magnaporthe_grisea	5	0.996
crotalus_atrox	4	0.866
musa_acuminata	4	0.997
phytolacca_americana	4	0.913
petromyzon_marinus	4	0.924
rhipicephalus_appendiculatus	4	0.795
crithidia_fasciculata	4	0.975
viscum_album	4	0.925
ipomoea_batatas	4	0.951
aplysia_californica	4	0.884
bungarus_multicinctus	4	0.774
fusarium_oxysporum	4	0.934
galdieria_sulphuraria	4	0.957
cavia_porcellus	4	0.966
anas_platyrhynchos	4	0.929
lamellibrachia_satsuma	4	0.826
rhodnius_prolixus	4	0.781
betula_pendula	4	0.852
populus_trichocarpa	4	0.833
bungarus_caeruleus	3	0.884
naja_kaouthia	3	0.902
capra_hircus	3	0.889
acanthamoeba_castellanii	3	0.942
discosoma_sp	3	0.881
branchiostoma_floridae	3	0.923
coffea_canephora	3	0.896
psophocarpus_tetragonolobus	3	0.908
thalassiosira_weissflogii	3	0.932
litopenaeus_vannamei	3	0.946
gibberella_zeae	3	0.955
spodoptera_frugiperda	3	0.871
ophiophagus_hannah	3	0.763
thermoascus_aurantiacus	3	0.982
hemiselmis_andersenii	3	0.792
agrocybe_aegerita	3	0.887
orectolobus_maculatus	3	0.746
antirrhinum_majus	3	0.813
vigna_unguiculata	3	0.886
penicillium_chrysogenum	3	0.912
fusarium_sporotrichioides	3	0.878
actinidia_chinensis	3	0.888
ustilago_maydis	3	0.929
daboia_siamensis	3	0.933
agkistrodon_contortrix	3	0.830
salmo_salar	3	0.942
anemonia_sulcata	3	0.856
oligobrachia_mashikoi	3	0.843
tachypleus_tridentatus	3	0.951
pachyrhizus_erosus	3	0.890
ginglymostoma_cirratum	3	0.849
ancylostoma_ceylanicum	3	0.925
melanocarpus_albomyces	3	1.003
eisenia_fetida	3	0.925
oncorhynchus_mykiss	3	0.897
magnaporthe_oryzae	3	0.923
gadus_morhua	3	0.915
doryteuthis_pealeii	3	0.804
humicola_grisea	3	0.980
argopecten_irradians	3	0.842
brassica_juncea	3	0.923
malassezia_sympodialis	3	0.948
bothrops_jararacussu	3	0.873
eptatretus_burgeri	3	0.898
cucurbita_moschata	2	0.886
nicotiana_glutinosa	2	0.908
mammuthus_primigenius	2	0.836
dasyatis_akajei	2	0.817
rhizomucor_miehei	2	0.923
phaseolus_angularis	2	0.976
macaca_mulatta	2	0.822
limulus_polyphemus	2	1.024
pneumocystis_carinii	2	0.883
dermatophagoides_farinae	2	0.807
aspergillus_parasiticus	2	0.933
choristoneura_fumiferana	2	0.963
cynara_cardunculus	2	0.966
meleagris_gallopavo	2	0.646
populus_nigra	2	0.939
trimeresurus_stejnegeri	2	0.901
yarrowia_lipolytica	2	0.944
gastrodia_elata	2	0.802
saccharomyces_pastorianus	2	0.943
griffithsia_monilis	2	0.871
secale_cereale	2	0.941
trichoderma_harzianum	2	0.991
paramecium_tetraurelia	2	0.896
komagataella_pastoris	2	0.856
humicola_insolens	2	0.913
mentha_piperita	2	0.864
trametes_versicolor	2	0.999
rhodomonas_sp	2	0.693
chroomonas_sp	2	0.742
sordaria_macrospora	2	0.922
cryphonectria_parasitica	2	1.035
galleria_mellonella	2	0.708

rhizopus_chinensis	2	0.928
amphidinium_carterae	2	0.676
tetrahymena_thermophila	2	0.717
neocallimastix_patriciarum	2	0.879
calystegia_sepium	2	0.906
echis_multisquamatus	2	0.897
hypogastrura_harveyi	2	1.065
diutina_rugosa	2	0.966
arenicola_marina	2	0.944
trichinella_spiralis	2	0.932
melampsora_lini	2	0.717
bubalus_bubalis	2	0.904
tetronarce_californica	2	1.024
plexaura_homomalla	2	0.885
lolium_perenne	2	0.941
chlamydomonas_moewusii	2	0.788
pichia_angusta	2	0.952
lumbricus_terrestris	2	0.821
dendroaspis_angusticeps	2	0.696
triatoma_infestans	2	0.804
kluyveromyces_marxianus	2	0.978
renilla_reniformis	2	0.869
blattella_germanica	2	0.897
podocnemis_unifilis	2	0.801
boiga_irregularis	2	0.787
laticauda_semifasciata	2	0.711
ichthyomyzon_unicuspis	2	0.794
bionectria_ochroleuca	2	0.955
oryzias_latipes	2	0.874
ancylostoma_caninum	2	0.687
thunnus_thynnus	2	0.817
perkinsus_marinus	2	0.911
remusatia_vivipara	2	0.984
brugia_malayi	2	0.931
aspergillus_awamori	2	0.917
rhizopus_niveus	2	0.933
saponaria_officinalis	2	0.937
agaricus_bisporus	2	0.919
thermomyces_lanuginosus	2	0.962
mustelus_griseus	2	0.816
zoarces_viviparus	2	0.725
vespula_vulgaris	2	0.846
schistosoma_japonicum	2	0.990
ostrinia_furnacalis	2	0.977
anopheles_cracens	2	0.873
pan_troglodytes	2	0.822
lodderomyces_elongisporus	2	0.896
zoanthus_sp	2	0.879
hirudo_medicinalis	2	0.896
sterkiella_nova	2	0.823
geotrichum_sp	2	1.031
columba_livia	2	0.944
tritrichomonas_foetus	2	0.947
scadoxus_multiflorus	2	1.026
lachancea_kluyveri	2	0.927
anopheles_stephensi	2	0.740
brassica_napus	2	0.984
mesocricetus_auratus	2	0.881
abrus_precatorius	2	0.913
wuchereria_bancrofti	2	0.839
ixodes_scapularis	2	0.833
placopecten_magellanicus	2	0.866
piromyces_equi	2	0.916
leishmania_tarentolae	2	0.841
clytia_gregaria	2	0.864
leishmania_braziliensis	2	0.871
branchiostoma_lanceolatum	2	0.934
calloselasma_rhodostoma	2	0.951
rhipicephalus_microplus	2	0.933
crotalus_durissus	2	0.868
argas_monolakensis	2	0.865
clitocybe_nebularis	2	0.821
dermatophagoides_pteronyssinus	2	0.871
holotrichia_diomphalia	2	0.867
haementeria_officinalis	2	0.898
mesobuthus_martensii	2	0.892
eremothecium_gossypii	2	0.799
millerozyma_farinosa	2	0.944
ornithodoros_moubata	2	0.766
hydra_vulgaris	2	0.942
gloydius_halys	2	0.972
macrolepiota_procera	2	0.865
schistosoma_haematobium	2	0.843
abies_grandis	2	0.879
urechis_caupo	2	0.924
haliotis_fulgens	2	0.806
pleurotus_ostreatus	2	0.815
penicillium_simplicissimum	2	0.984
malus_domestica	2	0.988
camelus_dromedarius	2	0.951
rhodosporidium_toruloides	2	0.941
datura_stramonium	2	0.920
echis_carinatus	2	0.797
peromyscus_maniculatus	2	0.829
talaromyces_funiculosus	2	0.974
anopheles_dirus	2	0.889
haliotis_rufescens	1	0.732
echinococcus_granulosus	1	0.776
pagrus_major	1	0.853
aspergillus_phoenicis	1	0.932
lethenteron_camtschaticum	1	0.887
trematomus_bernacchii	1	0.828
baliospermum_montanum	1	0.940

gossypium_hirsutum	1	0.794
sinapis_alba	1	0.964
aspergillus_kawachii	1	0.934
sporidiobolus_salmonicolor	1	0.935
bauhinia_bauhinioides	1	0.849
helicoverpa_armigera	1	0.908
chironomus_thummi	1	0.764
parengyodontium_album	1	0.988
candida_boidinii	1	0.995
penicillium_citrinum	1	0.949
trypanosoma_congolense	1	0.734
cycas_revoluta	1	0.931
morus_nigra	1	1.110
tetrahymena_pyriformis	1	0.774
malassezia_globosa	1	0.930
phoca_vitulina	1	0.623
huperzia_serrata	1	1.000
rigidoporus_microporus	1	0.974
cichorium_intybus	1	0.883
thaumatococcus_daniellii	1	1.039
hypocrea_virens	1	1.006
auricularia_auricula-judae	1	0.922
phascolopsis_gouldii	1	0.798
anopheles_albimanus	1	0.483
plectranthus_scutellarioides	1	0.972
onchocerca_volvulus	1	0.856
scytalidium_lignicola	1	0.916
stichodactyla_helianthus	1	0.975
penicillium_expansum	1	0.897
ustilago_sphaerogena	1	0.852
bothrops_asper	1	0.788
pseudechis_porphyriacus	1	0.851
ganoderma_lucidum	1	0.851
laternula_elliptica	1	0.801
talaromyces_minioluteus	1	1.078
ostertagia_ostertagi	1	0.774
leptosphaeria_maculans	1	0.753
curcuma_longa	1	0.944
tetraodon_nigroviridis	1	0.977
aspergillus_tubingensis	1	1.070
centruroides_sculpturatus	1	0.930
ctenopharyngodon_idella	1	0.766
aspergillus_ficuum	1	0.952
cimex_lectularius	1	0.921
cyanidioschyzon_merolae	1	0.834
petunia_hybrida	1	0.854
taenia_solium	1	1.017
juniperus_ashei	1	0.927
taxus_wallichiana	1	0.995
lacrymaria_velutina	1	0.983
paramecium_caudatum	1	0.786
griffonia_simplicifolia	1	0.971
sitophilus_oryzae	1	0.966
scophthalmus_maximus	1	0.773
spisula_solidissima	1	0.823
trichosurus_vulpecula	1	0.842
hyaloperonospora_parasitica	1	0.719
taxus_brevifolia	1	0.891
takifugu_rubripes	1	0.786
botrytis_aclada	1	0.945
boiga_dendrophila	1	0.707
vanderwaltozyma_polyspora	1	0.796
penicillium_canescens	1	0.940
phaeodactylum_tricornutum	1	0.757
helix_pomatia	1	0.927
pseudozyma_antarctica	1	0.909
sargassum_fusiforme	1	0.647
citrus_x	1	0.958
helianthus_tuberosus	1	1.017
momordica_balsamina	1	0.921
lyophyllum_decastes	1	0.976
babesia_divergens	1	0.857
antheraea_mylitta	1	0.778
ocimum_basilicum	1	0.910
lingulodinium_polyedrum	1	0.890
euplotes_raikovi	1	0.748
iris_hollandica	1	0.940
leiurus_quinquestriatus	1	0.946
gossypium_arboreum	1	0.830
enteroctopus_dofleini	1	0.871
chaetoceros_gracilis	1	0.667
alvinella_pompejana	1	1.006
avena_sativa	1	0.705
carcinoscorpius_rotundicauda	1	0.784
populus_tremuloides	1	0.985
hottentotta_judaicus	1	0.779
salvia_officinalis	1	0.910
centruroides_exilicauda	1	0.877
trichosanthes_kirilowii	1	0.944
galaxea_fascicularis	1	0.805
galdieria_partita	1	0.906
struthio_camelus	1	0.999
macaca_fascicularis	1	0.928
stichopus_japonicus	1	0.883
lutzomyia_longipalpis	1	0.731
loxosceles_intermedia	1	0.905
millerozyma_acaciae	1	0.817
macrobdella_decora	1	1.029
purpureocillium_lilacinum	1	0.982
capparis_masaikai	1	0.912
saccharomycopsis_fibuligera	1	0.891
cryptococcus_sp	1	1.019
vicia_narbonensis	1	0.890

epiphyas_postvittana	1	0.837
parthenium_argentatum	1	0.878
cupiennius_salei	1	0.976
trichoderma_citrinoviride	1	0.931
scaptomyza_nigrita	1	0.850
pachysandra_terminalis	1	0.915
parkia_platycephala	1	1.022
candida_cylindracea	1	0.953
lens_culinaris	1	1.006
pseudopleuronectes_americanus	1	0.642
phialidium_sp	1	0.861
strongyloides_stercoralis	1	0.755
leucoagaricus_meleagris	1	0.937
piromyces_rhizinflatus	1	0.839
suregada_multiflora	1	0.859
digitalis_lanata	1	0.888
lobophyllia_hemprichii	1	0.856
geodia_cydonium	1	0.891
cordyceps_militaris	1	0.989
epiactis_japonica	1	0.876
plodia_interpunctella	1	0.845
locusta_migratoria	1	0.812
aloe_arborescens	1	0.978
citrus_sinensis	1	1.052
lupinus_angustifolius	1	0.970
ginkgo_biloba	1	0.744
amphitrite_ornata	1	0.748
pieris_brassicae	1	0.901
solanum_cardiophyllum	1	0.857
polygonatum_cyrtonema	1	0.970
rhipicephalus_bursa	1	1.008
erythrina_crista-galli	1	0.975
aspergillus_niveus	1	0.883
entacmaea_quadricolor	1	0.816
vipera_ammodytes	1	0.817
alternaria_alternata	1	0.882
pleurotus_cornucopiae	1	0.985
bemisia_argentifolii	1	0.970
aegilops_tauschii	1	0.810
alcyonium	1	0.842
sarocladium_strictum	1	0.922
coriolopsis_caperata	1	1.000
trachyphyllia_geoffroyi	1	0.859
typhula_ishikariensis	1	1.034
conger_myriaster	1	1.000
arthromyces_ramosus	1	0.932
gerbera_hybrida	1	0.978
limnoria_quadripunctata	1	0.983
bougainvillea_spectabilis	1	0.906
vigna_radiata	1	0.798
lentinus_tigrinus	1	1.019
cochliobolus_lunatus	1	0.945
anthomedusae_sp	1	0.835
actinia_equina	1	0.933
marchantia_polymorpha	1	0.952
mesobuthus_tamulus	1	0.849
dianthus_caryophyllus	1	0.901
meyerozyma_guilliermondii	1	0.848
toxascaris_leonina	1	0.875
neurospora_sitophila	1	0.905
cryptopygus_antarcticus	1	0.941
branchiostoma_belcheri	1	0.804
plasmodium_(laverania)	1	0.798
nectria_haematococca	1	0.943
grifola_frondosa	1	0.883
penicillium_sp	1	0.991
dromaius_novaehollandiae	1	0.786
aspergillus_flavus	1	0.941
trypanosoma_rangeli	1	0.988
fungia_concinna	1	0.820
discosoma_striata	1	0.875
dugesia_japonica	1	0.821
mitrocoma_cellularia	1	0.876
leptoxyphium_fumago	1	0.802
ophiostoma_piceae	1	1.013
cucumaria_echinata	1	0.959
nilaparvata_lugens	1	0.874
manihot_esculenta	1	0.924
colletotrichum_lupini	1	1.127
saguinus_oedipus	1	0.856
amaranthus_caudatus	1	0.851
culex_quinquefasciatus	1	0.775
androctonus_australis	1	0.992
amyelois_transitella	1	0.814
picea_abies	1	0.867
leishmania_sp	1	0.979
loxosceles_laeta	1	0.948
fragaria_ananassa	1	0.744
mytilus_galloprovincialis	1	1.019
rhodotorula_graminis	1	0.944
sarcocystis_muris	1	0.872
micromonas_commoda	1	0.822
beta_vulgaris	1	0.984
aldabrachelys_gigantea	1	0.822
allium_sativum	1	0.971
daucus_carota	1	0.951
helicoverpa_zea	1	0.916
talaromyces_purpureogenus	1	1.052
xerocomellus_chrysenteron	1	0.994
pontellina_plumata	1	0.946
bothrops_moojeni	1	0.847
anemonia_manjano	1	0.904
aleuria_aurantia	1	0.964

rhodotorula_mucilaginosa	1	0.990
armoracia_rusticana	1	0.934
astacus_leptodactylus	1	0.973
malbranchea_cinnamomea	1	0.945
carassius_auratus	1	0.719
ashbya_gossypii	1	0.875
rhizopus_oryzae	1	0.812
pecten_maximus	1	0.839
rheum_palmatum	1	0.990
rhizomucor_pusillus	1	0.983
acidomyces_acidophilus	1	0.937
fusarium_solani	1	0.998
penicillium_roqueforti	1	0.758
punica_granatum	1	0.954
unclassified_mammalia	1	0.858
sinularia_lochmodes	1	0.855
guillardia_theta	1	0.769
molineria_latifolia	1	0.992
champsocephalus_gunnari	1	1.019
tityus_serrulatus	1	0.895
trema_tomentosa	1	0.767
talaromyces_emersonii	1	0.998
linum_nodiflorum	1	0.937
eschscholzia_californica	1	0.941
leucosporidium_sp	1	0.979
chlamys_nipponensis	1	0.781
curvularia_inaequalis	1	0.914
brachypodium_distachyon	1	0.873
coccomyxa_sp	1	0.960
cerebratulus_lacteus	1	0.718
fagopyrum_esculentum	1	0.749
elephantulus_edwardii	1	0.988
taenia_saginata	1	0.833
helianthus_annuus	1	0.955
chondrostereum_purpureum	1	1.140
scheffersomyces_stipitis	1	0.946
anadara_inaequivalvis	1	0.811
cyanidioschyzon_sp	1	0.916
ambystoma_tigrinum	1	0.812
daboia_russelii	1	0.769
pyrus_pyrifolia	1	0.745
geotrichum_candidum	1	0.947
centruroides_noxius	1	1.064
paecilomyces_sp	1	1.021
clitoria_ternatea	1	0.952
brevicoryne_brassicae	1	0.966
pinus_taeda	1	0.950
sparus_aurata	1	0.968
ascaris_lumbricoides	1	0.651
rhyparobia_maderae	1	0.900
potentilla_atrosanguinea	1	0.972
chrysanthemum_morifolium	1	0.879
penaeus_japonicus	1	0.893
humulus_lupulus	1	0.876
astacus_astacus	1	0.859
tropaeolum_majus	1	0.917
linum_usitatissimum	1	0.899
gibberella_moniliformis	1	1.044
photinus_pyralis	1	0.986
rhagium_inquisitor	1	1.046
protobothrops_mucrosquamatus	1	0.910
nematostella_vectensis	1	0.888
lycopersicon_hirsutum	1	0.938
narcissus_pseudonarcissus	1	0.992
schistocerca_gregaria	1	0.771
volvariella_volvacea	1	0.763
drosophila_mojavensis	1	0.928
clarkia_breweri	1	0.907
aplysia_kurodai	1	0.935
trypanosoma_vivax	1	0.975
gibberella_fujikuroi	1	0.947
obelia_longissima	1	0.914
heligmosomoides_polygyrus	1	0.852
sclerotinia_sclerotiorum	1	0.963
actinidia_deliciosa	1	0.956
thuja_plicata	1	0.861
heteractis_crispa	1	0.879
trametes_hirsuta	1	1.015
bjerkandera_adusta	1	0.878
anguilla_japonica	1	0.841
ciona_intestinalis	1	0.947
pterocarpus_angolensis	1	1.023
polyporus_squamosus	1	0.899
aspergillus_clavatus	1	0.967
nicotiana_alata	1	0.621
tabanus_yao	1	0.798
ruditapes_philippinarum	1	1.011
pythium_aphanidermatum	1	0.888
lepidium_virginicum	1	0.865
cyprinus_carpio	1	1.025
trametopsis_cervina	1	0.909
rangifer_tarandus	1	0.807
parasponia_andersonii	1	0.694
pseudonaja_textilis	1	0.841
mytilus_edulis	1	0.945
pyropia_yezoensis	1	0.675
citrullus_lanatus	1	0.925
larrea_tridentata	1	0.540
sambucus_nigra	1	0.868
colletotrichum_lindemuthianum	1	0.884
momordica_charantia	1	0.914
populus_tomentosa	1	0.935
argas_reflexus	1	0.848

phytophthora_cryptogea	1	0.815
petroselinum_crispum	1	0.941
capsicum_annuum	1	0.957
passalora_fulva	1	0.907
coriolopsis_trogii	1	1.017
candida_tenuis	1	0.989
favia_favus	1	0.839
pycnoporus_cinnabarinus	1	1.010
actinidia_eriantha	1	0.962
euprosthenops_australis	1	0.857
aspergillus_japonicus	1	0.933
flaveria_trinervia	1	0.903
gemmabryum_coronatum	1	0.892
glycera_dibranchiata	1	0.872
biomphalaria_glabrata	1	0.881
neotermes_koshunensis	1	0.941
sabellastarte_magnifica	1	0.906
nasutitermes_takasagoensis	1	1.030
anopheles_funestus	1	0.904
argyrosomus_regius	1	0.701
naja_naja	1	0.888
freesia_hybrid	1	0.991
dendronephthya_sp	1	0.840
schwanniomyces_capriottii	1	0.928
chenopodium_album	1	0.872
antheraea_pernyi	1	0.955
namalycastis_sp	1	0.916
pyrenophora_tritici-repentis	1	0.806
serendipita_indica	1	0.989
camellia_sinensis	1	0.970
chiridius_poppei	1	0.873
clupea_harengus	1	0.844
drosophila_lebanonensis	1	0.984
clonorchis_sinensis	1	0.851
cynodon_dactylon	1	0.932
cerianthus_membranaceus	1	0.871
papaver_somniferum	1	0.909
pandalus_borealis	1	0.861
lachancea_thermotolerans	1	0.747
pneumocystis_jiroveci	1	0.833
phacoides_pectinatus	1	0.786
naganishia_liquefaciens	1	1.044
beroe_abyssicola	1	0.779
pseudoplectania_nigrella	1	0.936
corallina_pilulifera	1	0.921
pelophylax_perezi	1	1.005
echinophyllia_sp	1	0.841
erythrina_corallodendron	1	0.895
davidiella_tassiana	1	0.965
renilla_muelleri	1	0.847
sepia_officinalis	1	0.851
colocasia_esculenta	1	0.663
laetiporus_sulphureus	1	0.908
silene_latifolia	1	0.901
vipera_aspis	1	0.730
acanthamoeba	1	0.793
porphyridium_purpureum	1	0.983
tuber_borchii	1	0.706
prunus_dulcis	1	0.978
debaryomyces_hansenii	1	0.810
cucurbita_maxima	1	0.937
symbiotic_protist	1	0.972
hypericum_perforatum	1	0.769
ixodes_ricinus	1	0.921
podophyllum_peltatum	1	0.990
plasmodium_berghei	1	0.797
montipora_sp	1	0.847
lucilia_cuprina	1	0.917
claviceps_purpurea	1	0.877
galanthus_nivalis	1	0.972
brassica_campestris	1	0.924
fusarium_sp	1	1.069
prunus_avium	1	0.952
haemonchus_contortus	1	0.874
urtica_dioica	1	0.926
lymnaea_stagnalis	1	0.877
gloydius_saxatilis	1	0.933
pectiniidae	1	0.805
mamestra_brassicae	1	0.754
ophiostoma_novo-ulmi	1	0.808
gloeophyllum_trabeum	1	0.903
pinus_sylvestris	1	1.002
trichosporon_cutaneum	1	1.029
aequorea_coerulescens	1	0.866
trametes_ochracea	1	0.931
mesembryanthemum_crystallinum	1	0.879
phytophthora_infestans	1	0.779
ulex_europaeus	1	1.032
plasmodium_cynomolgi	1	0.872
chlorella_variabilis	1	0.918
pseudo-nitzschia_multiseries	1	0.855
morone_saxatilis	1	0.979
tamarindus_indica	1	0.862
pleurotus_eryngii	1	0.869
nototodarus_sloanii	1	0.880
strongylocentrotus_purpuratus	1	0.853
drosophila_mauritiana	1	0.852
marasmius_oreades	1	0.915
macrophoma_commelinae	1	1.088
brachyopsis_rostratus	1	0.865
myrothecium_verrucaria	1	0.759
hyacinthoides_hispanica	1	1.004
codakia_orbicularis	1	0.765

aspergillus_restrictus	1	0.851
luciola_cruciata	1	0.952
hypoderma_lineatum	1	1.017
thielavia_terrestris	1	0.908
thalictrum_flavum	1	0.788
aplysia_limacina	1	0.796
cyanophora_paradoxa	1	0.757
gersemia_fruticosa	1	0.877
lecanicillium_psalliotae	1	0.974
naumovozyma_dairenensis	1	0.853
oxyuranus_scutellatus	1	0.815
tetradesmus_obliquus	1	0.974

Table S11. List of bacterial organisms in the dataset $\mathcal{D}$, with their number of proteins and average $\Delta\Delta G$ upon all possible point mutations

Organism	Number of proteins	$\langle \Delta\Delta G \rangle$ (kcal/mol)
escherichia_coli	1180	0.919
thermus_thermophilus	419	0.953
bacillus_subtilis	400	0.906
mycobacterium_tuberculosis	394	0.918
pseudomonas_aeruginosa	354	0.913
thermotoga_maritima	328	0.951
staphylococcus_aureus	282	0.877
salmonella_typhimurium	202	0.920
streptococcus_pneumoniae	160	0.900
bacteroides_thetaiotaomicron	142	0.897
vibrio_cholerae	141	0.907
bacillus_anthracis	132	0.915
aquifex_aeolicus	121	0.945
helicobacter_pylori	115	0.891
enterococcus_faecalis	101	0.888
pseudomonas_putida	94	0.948
streptomyces_coelicolor	93	0.910
agrobacterium_fabrum	92	0.913
mycobacterium_smegmatis	90	0.926
haemophilus_influenzae	88	0.913
bacillus_cereus	84	0.888
bacteroides_fragilis	76	0.885
streptococcus_pyogenes	75	0.867
geobacillus_stearothermophilus	74	0.950
deinococcus_radiodurans	73	0.906
neisseria_meningitidis	71	0.895
yersinia_pestis	69	0.914
klebsiella_pneumoniae	66	0.923
bacillus_halodurans	64	0.908
synechocystis_sp	64	0.917
burkholderia_pseudomallei	62	0.920
listeria_monocytogenes	62	0.887
clostridium_thermocellum	61	0.948
campylobacter_jejuni	61	0.907
legionella_pneumophila	59	0.859
rhodopseudomonas_palustris	58	0.899
corynebacterium_glutamicum	54	0.909
streptococcus_mutans	54	0.889
burkholderia_thailandensis	53	0.939
rhizobium_meliloti	52	0.945
shewanella_oneidensis	50	0.872
xanthomonas_campestris	50	0.909
burkholderia_cenocepacia	48	0.957
pseudomonas_fluorescens	48	0.934
porphyromonas_gingivalis	47	0.913
parabacteroides_distasonis	47	0.900
clostridium_perfringens	47	0.873
pseudomonas_syringae	46	0.876
rhodobacter_sphaeroides	45	0.915
vibrio_parahaemolyticus	45	0.855
coxiella_burnetii	42	0.915
peptoclostridium_difficile	41	0.900
bacteroides_vulgatus	41	0.874
bacteroides_ovatus	40	0.894
ruegeria_pomeroyi	39	0.913
rhodococcus_jostii	39	0.893
geobacillus_kaustophilus	38	0.936
clostridium_acetobutylicum	37	0.914
cupriavidus_necator	36	0.923
nostoc_sp	36	0.882
lactococcus_lactis	35	0.919
francisella_tularensis	35	0.921
brucella_abortus	34	0.944
pseudomonas_sp	34	0.960
bacillus_sp	34	0.956
desulfovibrio_vulgaris	33	0.920
streptomyces_sp	31	0.939
geobacter_sulfurreducens	31	0.837
thermosynechococcus_elongatus	31	0.903
chromobacterium_violaceum	30	0.910
novosphingobium_aromaticivorans	30	0.902
nitrosomonas_europaea	30	0.857
paraburkholderia_xenovorans	30	0.945
rhizobium_loti	28	0.923
shigella_flexneri	27	0.832
synechococcus_elongatus	26	0.901
clostridium_botulinum	26	0.851
bordetella_bronchiseptica	26	0.930
anabaena_variabilis	26	0.918
streptococcus_agalactiae	25	0.882
neisseria_gonorrhoeae	25	0.895
mycobacterium_abscessus	25	0.931

paracoccus_denitrificans	25	0.934
bartonella_henselae	24	0.941
yersinia_enterocolitica	24	0.906
streptomyces_avermitilis	24	0.915
bacteroides_uniformis	24	0.889
mycobacterium_marinum	23	0.935
lactobacillus_plantarum	23	0.894
rhizobium_radiobacter	23	0.918
brucella_melitensis	22	0.946
bordetella_pertussis	22	0.916
listeria_innocua	22	0.875
acinetobacter_baumannii	22	0.905
azotobacter_vinelandii	22	0.950
thermobifida_fusca	21	0.955
corynebacterium_diphtheriae	21	0.886
bacillus_thuringiensis	21	0.854
staphylococcus_epidermidis	21	0.865
caldanaerobacter_subterraneus	20	0.915
nostoc_punctiforme	20	0.938
pectobacterium_atrosepticum	20	0.912
bifidobacterium_longum	19	0.941
synechococcus_sp	19	0.927
mycobacterium_paratuberculosis	19	0.957
cytophaga_hutchinsonii	19	0.876
uncultured_bacterium	18	0.942
alicyclobacillus_acidocaldarius	17	0.931
desulfovibrio_desulfuricans	17	0.889
caulobacter_vibrioides	16	0.935
serratia_marcescens	16	0.945
mycobacterium_avium	16	0.939
yersinia_pseudotuberculosis	16	0.909
rhodospirillum_rubrum	16	0.923
bacillus_licheniformis	15	0.938
desulfitobacterium_hafniense	15	0.909
thermus_aquaticus	15	0.956
caulobacter_crescentus	15	0.914
exiguobacterium_sibiricum	15	0.881
streptomyces_clavuligerus	15	0.962
lactobacillus_acidophilus	15	0.918
arthrobacter_sp	14	0.954
zobellia_galactanivorans	14	0.902
pseudomonas_stutzeri	14	0.918
rhodobacter_capsulatus	14	0.934
treponema_pallidum	14	0.919
colwellia_psychrerythraea	14	0.876
chlorobium_tepidum	14	0.869
chlamydia_trachomatis	14	0.862
cellvibrio_japonicus	13	0.976
streptococcus_thermophilus	13	0.863
streptomyces_lividans	13	0.934
acinetobacter_baylyi	13	0.909
ruegeria_sp	13	0.920
chlorobaculum_tepidum	13	0.892
sphaerobacter_thermophilus	13	0.960
rhodococcus_sp	13	0.938
bordetella_parapertussis	13	0.908
chloroflexus_aurantiacus	13	0.922
vibrio_vulnificus	13	0.905
methylococcus_capsulatus	12	0.914
marinobacter_hydrocarbonoclasticus	12	0.867
clostridioides_difficile	12	0.847
prochlorococcus_marinus	12	0.913
borrelia_burgdorferi	12	0.865
sphingomonas_sp	12	0.930
planctopirus_limnophila	12	0.926
streptomyces_venezuelae	12	0.951
zymomonas_mobilis	12	0.949
leptospira_interrogans	12	0.904
bacteroides_caccae	12	0.913
shewanella_loihica	12	0.861
bacillus_circulans	12	0.957
methylobacillus_flagellatus	11	0.900
vibrio_fischeri	11	0.904
xylella_fastidiosa	11	0.889
dickeya_chrysanthemi	11	0.948
micromonospora_echinospora	11	0.935
brucella_suis	11	0.962
amycolatopsis_orientalis	11	0.897
citrobacter_freundii	11	0.932
stenotrophomonas_maltophilia	11	0.929
moorella_thermoacetica	11	0.962
bacillus_megaterium	11	0.925
enterococcus_faecium	11	0.913
shewanella_amazonensis	11	0.885
jannaschia_sp	11	0.921
bacillus_amyloliquefaciens	11	0.942
desulfovibrio_alaskensis	11	0.898
borreliella_burgdorferi	11	0.853
polaromonas_sp	10	0.935
methylobacterium_extorquens	10	0.922
kribbella_flavida	10	0.920
salmonella_typhi	10	0.934
aeromonas_hydrophila	10	0.886
anabaena_sp	10	0.863
cupriavidus_metallidurans	10	0.810
sphingomonas_paucimobilis	10	0.947
enterobacter_cloacae	10	0.910
comamonas_testosteroni	10	0.967
parabacteroides_merdae	10	0.895
allochromatium_vinosum	10	0.933
bacteroides_eggerthii	10	0.858
ruminococcus_gnavus	9	0.919
bradyrhizobium_diazoefficiens	9	0.910

pseudomonas_savastanoi	9	0.913
geobacillus_thermodenitrificans	9	0.904
bifidobacterium_adolescentis	9	0.910
geobacter_metallireducens	9	0.933
veillonella_parvula	9	0.927
shewanella_frigidimarina	9	0.878
paenarthrobacter_aurescens	9	0.935
wolinella_succinogenes	9	0.898
mycobacterium_leprae	9	0.890
rhodothermus_marinus	9	0.921
proteus_mirabilis	8	0.892
xanthomonas_oryzae	8	0.922
ochrobactrum_anthropi	8	0.962
desulfovibrio_gigas	8	0.906
pseudoalteromonas_sp	8	0.907
clostridium_cellulolyticum	8	0.951
paenibacillus_polymyxa	8	0.954
vibrio_harveyi	8	0.946
gloeobacter_violaceus	8	0.917
xanthobacter_autotrophicus	8	0.987
alcaligenes_faecalis	8	1.024
rhodococcus_erythropolis	8	0.934
shewanella_denitrificans	8	0.927
bradyrhizobium_japonicum	8	0.928
myxococcus_xanthus	8	0.921
klebsiella_oxytoca	8	0.951
rhizobium_etli	8	0.923
carboxydothermus_hydrogenoformans	8	0.978
burkholderia_cepacia	7	0.935
fusobacterium_nucleatum	7	0.791
burkholderia_ambifaria	7	0.912
xanthomonas_axonopodis	7	0.925
streptomyces_antibioticus	7	0.917
shewanella_baltica	7	0.864
lactobacillus_paracasei	7	0.934
chromohalobacter_salexigens	7	0.946
roseobacter_denitrificans	7	0.891
xanthomonas_citri	7	0.910
acidaminococcus_fermentans	7	0.943
pectobacterium_carotovorum	7	0.940
lactobacillus_brevis	7	0.916
agrobacterium_vitis	7	0.948
proteus_vulgaris	7	0.944
ralstonia_solanacearum	7	0.912
oenococcus_oeni	7	0.903
pseudomonas_mendocina	7	0.905
streptomyces_griseus	7	0.970
acidithiobacillus_ferrooxidans	7	0.921
lactobacillus_casei	6	0.944
clostridium_pasteurianum	6	0.977
saccharophagus_degradans	6	0.912
salmonella_choleraesuis	6	1.017
streptomyces_hygroscopicus	6	0.928
streptococcus_gordonii	6	0.895
thauera_aromatica	6	0.938
staphylococcus_saprophyticus	6	0.922
methylophilus_methylotrophus	6	0.976
actinoplanes_teichomyceticus	6	0.842
thermomonospora_curvata	6	0.943
klebsiella_aerogenes	6	0.984
mycobacterium_thermoresistibile	6	0.936
hathewaya_histolytica	6	0.901
streptococcus_suis	6	0.925
treponema_denticola	6	0.925
corynebacterium_sp	6	0.922
haemophilus_somnus	6	0.870
mycobacterium_ulcerans	6	0.960
mycoplasma_pneumoniae	6	0.799
pseudoalteromonas_haloplanktis	6	0.938
anaplasma_phagocytophilum	6	0.946
eubacterium_barkeri	6	0.948
anaerococcus_prevotii	5	0.906
bdellovibrio_bacteriovorus	5	0.854
psychrobacter_arcticus	5	0.923
aromatoleum_aromaticum	5	0.974
arthrospira_platensis	5	0.921
propionibacterium_freudenreichii	5	0.950
agathobacter_rectalis	5	0.875
clostridium_tetani	5	0.875
burkholderia_multivorans	5	0.949
sporosarcina_pasteurii	5	0.953
nocardia_farcinica	5	0.885
hahella_chejuensis	5	0.934
arthrobacter_globiformis	5	0.966
lactobacillus_reuteri	5	0.873
streptomyces_viridochromogenes	5	0.980
pedobacter_heparinus	5	0.913
actinobacillus_succinogenes	5	0.967
actinobacillus_pleuropneumoniae	5	0.861
methylibium_petroleiphilum	5	0.958
rickettsia_felis	5	0.895
lactobacillus_salivarius	5	0.907
streptomyces_avidinii	5	0.913
aggregatibacter_actinomycetemcomitans	5	0.913
staphylococcus_haemolyticus	5	0.887
streptomyces_fradiae	5	0.937
hyphomicrobium_denitrificans	5	0.969
rhodococcus_opacus	5	0.895
paraburkholderia_phymatum	5	0.976
streptomyces_albus	5	0.946
elizabethkingia_meningoseptica	5	0.982
micrococcus_luteus	5	0.977
ralstonia_pickettii	5	1.000

paracoccus_pantotrophus	5	0.923
streptomyces_lavendulae	5	0.955
acetobacter_aceti	5	0.972
saccharopolyspora_spinosa	4	0.965
leptotrichia_buccalis	4	0.926
lactobacillus_delbrueckii	4	0.987
acidothermus_cellulolyticus	4	1.020
thiobacillus_thioparus	4	0.947
lysinibacillus_sphaericus	4	0.911
saccharopolyspora_erythraea	4	0.895
sphingobium_yanoikuyae	4	0.918
vibrio_sp	4	0.958
photorhabdus_luminescens	4	0.868
paracoccus_versutus	4	0.915
nitrosospira_multiformis	4	0.940
sulfurospirillum_deleyianum	4	0.873
pseudoalteromonas_atlantica	4	0.932
thermoactinomyces_vulgaris	4	0.986
moorea_producens	4	0.904
clostridium_cellulovorans	4	0.994
mycoplasma_penetrans	4	0.919
haemophilus_ducreyi	4	0.950
caldicellulosiruptor_bescii	4	0.979
flavobacterium_sp	4	0.945
gluconobacter_oxydans	4	0.934
anaeromyxobacter_dehalogenans	4	0.875
jonesia_denitrificans	4	0.910
pasteurella_multocida	4	0.885
desulfovibrio_africanus	4	0.892
starkeya_novella	4	0.897
syntrophus_aciditrophicus	4	0.854
geobacillus_sp	4	0.983
shewanella_pealeana	4	0.972
mycoplasma_genitalium	4	0.825
pediococcus_pentosaceus	4	0.818
streptomyces_halstedii	4	0.960
eubacterium_ventriosum	4	0.933
shewanella_sp	4	0.877
streptomyces_globisporus	4	0.929
ehrlichia_chaffeensis	4	0.922
syntrophomonas_wolfei	4	0.895
idiomarina_loihiensis	4	0.918
halothiobacillus_neapolitanus	4	0.892
roseovarius_nubinhibens	4	0.989
bacillus_pumilus	4	0.901
thiobacillus_denitrificans	4	0.956
caldanaerobius_polysaccharolyticus	4	0.906
lachnoclostridium_phytofermentans	4	0.941
azospirillum_brasilense	4	0.969
magnetospirillum_magneticum	4	0.832
streptococcus_dysgalactiae	4	0.790
haliangium_ochraceum	4	0.882
thermoanaerobacterium_thermosaccharolyticum	4	0.938
dyadobacter_fermentans	4	0.920
rickettsia_prowazekii	4	0.947
thermotoga_neapolitana	4	0.975
verminephrobacter_eiseniae	3	0.925
oligotropha_carboxidovorans	3	1.049
streptomyces_lasaliensis	3	0.891
achromobacter_cycloclastes	3	0.973
agrobacterium_radiobacter	3	0.990
clostridium_symbiosum	3	1.000
aliivibrio_salmonicida	3	0.862
acinetobacter_calcoaceticus	3	0.988
acetivibrio_cellulolyticus	3	0.874
methylacidiphilum_infernorum	3	0.783
enterobacter_agglomerans	3	0.900
hydrogenobacter_thermophilus	3	0.863
saccharomonospora_viridis	3	0.902
caldicellulosiruptor_saccharolyticus	3	0.891
thermus_caldophilus	3	1.002
photobacterium_leiognathi	3	0.870
paenarthrobacter_nicotinovorans	3	0.953
desulfomicrobium_norvegicum	3	0.962
clostridium_leptum	3	0.822
desulfovibrio_piger	3	0.893
lechevalieria_aerocolonigenes	3	0.925
oceanobacillus_iheyensis	3	0.927
komagataeibacter_xylinus	3	0.866
streptomyces_verticillus	3	0.817
chlamydia_pneumoniae	3	0.847
halothermothrix_orenii	3	0.956
enterococcus_gallinarum	3	0.939
sorangium_cellulosum	3	0.917
burkholderia_mallei	3	0.915
citrobacter_braakii	3	0.914
thermochromatium_tepidum	3	0.908
aeribacillus_pallidus	3	1.011
thermoanaerobacterium_saccharolyticum	3	0.943
rickettsia_typhi	3	0.867
ralstonia_sp	3	0.901
conexibacter_woesei	3	0.940
burkholderia_lata	3	0.919
streptomyces_cattleya	3	1.034
streptococcus_sp	3	0.830
prochloron_didemni	3	0.918
dickeya_dadantii	3	0.796
alcaligenes_sp	3	0.954
ruminococcus_flavefaciens	3	0.841
clostridium_stercorarium	3	0.935
bradyrhizobium_sp	3	0.918
spirosoma_linguale	3	0.913
rhodococcus_rhodochrous	3	0.896

pseudomonas_pavonaceae	3	0.955
thermoanaerobacter_pseudethanolicus	3	0.971
thermotoga_petrophila	3	1.024
magnetospirillum_gryphiswaldense	3	0.792
hydrogenophaga_pseudoflava	3	0.985
oceanicola_granulosus	3	0.886
aneurinibacillus_thermoaerophilus	3	0.930
streptomyces_glaucescens	3	0.815
bacillus_clausii	3	0.949
eggerthella_lenta	3	0.932
sphingomonas_wittichii	3	0.954
pseudomonas_fragi	3	0.945
streptomyces_nogalater	3	0.847
aeromonas_salmonicida	3	0.890
mycobacterium_fortuitum	3	0.945
mastigocladus_laminosus	3	0.833
streptomyces_aureofaciens	3	0.920
rhodopirellula_baltica	3	0.913
streptomyces_sviceus	3	0.984
burkholderia_glumae	3	0.904
streptomyces_galilaeus	3	0.896
sulfitobacter_sp	3	0.876
cellvibrio_mixtus	3	0.893
amycolatopsis_mediterranei	3	0.934
acinetobacter_sp	3	0.909
clostridium_novyi	3	0.961
desulfotalea_psychrophila	3	0.905
clostridium_beijerinckii	3	1.015
bacillus_intermedius	3	0.817
streptomyces_carzinostaticus	3	0.904
deinococcus_geothermalis	3	0.923
ureaplasma_parvum	3	0.934
streptomyces_exfoliatus	3	0.975
burkholderia_sp	3	0.927
streptomyces_purpurascens	3	0.887
moraxella_catarrhalis	3	0.931
streptomyces_tendae	3	0.965
shewanella_putrefaciens	3	0.815
dichelobacter_nodosus	3	0.934
desulfovibrio_salexigens	3	0.905
corynebacterium_ammoniagenes	3	0.891
rhodovulum_sulfidophilum	2	0.883
yersinia_entomophaga	2	0.902
aeromonas_caviae	2	0.905
halomonas_sp	2	0.948
citrobacter_sp	2	0.905
eubacterium_cellulosolvens	2	0.960
aerococcus_viridans	2	0.974
paenibacillus_macerans	2	0.955
actinomadura_kijaniata	2	0.910
plesiocystis_pacifica	2	0.990
moraxella_sp	2	0.954
microcystis_aeruginosa	2	0.952
providencia_rettgeri	2	0.938
comamonas_sp	2	0.898
methylosinus_trichosporium	2	0.913
oxalobacter_formigenes	2	1.000
vibrio_proteolyticus	2	0.976
cellulomonas_fimi	2	0.975
desulfovibrio_fructosivorans	2	0.995
micromonospora_chersina	2	0.884
antarctic_bacterium	2	0.906
shigella_dysenteriae	2	0.960
bartonella_quintana	2	0.800
clostridium_scindens	2	0.934
rhizobium_leguminosarum	2	0.951
streptomyces_plicatus	2	1.041
tolypothrix_sp	2	0.913
alkaliphilus_metalliredigens	2	0.888
paraburkholderia_graminis	2	0.911
streptomyces_castaneoglobisporus	2	0.880
clostridium_scatologenes	2	0.952
aliivibrio_fischeri	2	0.868
mycoplasma_synoviae	2	0.964
streptomyces_diastaticus	2	0.931
rhizobium_sp	2	0.977
shewanella_benthica	2	0.913
agrobacterium_sp	2	0.962
bacillus_caldolyticus	2	0.932
alkaliphilus_oremlandii	2	0.838
brucella_ovis	2	0.907
clostridium_butyricum	2	0.946
thermovibrio_ammonificans	2	0.979
xenorhabdus_nematophila	2	0.882
bifidobacterium_bifidum	2	0.937
lactobacillus_johnsonii	2	0.954
planktothrix_agardhii	2	0.998
chitinophaga_pinensis	2	0.942
paenibacillus_barcinonensis	2	0.906
francisella_philomiragia	2	0.931
nakamurella_multipartita	2	0.942
streptomyces_caespitosus	2	0.950
bacillus_selenitireducens	2	0.900
eubacterium_eligens	2	0.905
paraburkholderia_rhizoxinica	2	0.992
acetoanaerobium_sticklandii	2	0.847
paenibacillus_sp	2	0.914
bacteroides_plebeius	2	0.862
alcanivorax_borkumensis	2	0.812
pseudobacteroides_cellulosolvens	2	0.906
sebaldella_termitidis	2	0.948
salmonella_paratyphi	2	0.991
phormidium_laminosum	2	0.909

selenomonas_ruminantium	2	0.948
chelativorans_sp	2	0.960
halorhodospira_halophila	2	0.879
blastochloris_viridis	2	0.918
mycobacterium_vanbaalenii	2	0.817
salmonella_enterica	2	0.908
vibrio_alginolyticus	2	0.829
bacillus_agaradhaerens	2	1.002
streptococcus_parasanguinis	2	0.960
lysobacter_enzymogenes	2	1.002
rickettsia_rickettsii	2	0.995
thermanaerovibrio_acidaminovorans	2	0.930
streptomyces_scabiei	2	0.852
salmonella_dublin	2	0.842
streptomyces_cyanogenus	2	0.996
desulfomicrobium_baculatum	2	0.987
phormidium_lapideum	2	1.024
actinomyces_naeslundii	2	0.809
marinomonas_primoryensis	2	0.914
microchaete_diplosiphon	2	0.818
photobacterium_profundum	2	0.892
janthinobacterium	2	0.918
staphylococcus_carnosus	2	0.789
symbiobacterium_thermophilum	2	1.017
enterococcus_mundtii	2	0.945
photobacterium_sp	2	0.837
halomonas_elongata	2	0.906
catenulispora_acidiphila	2	0.847
streptomyces_himastatinicus	2	0.865
enterococcus_hirae	2	0.961
bartonella_schoenbuchensis	2	0.794
streptomyces_argillaceus	2	0.977
clostridium_cochlearium	2	1.056
salinibacter_ruber	2	1.003
nonomuraea_sp	2	0.904
streptomyces_actuosus	2	0.886
burkholderia_vietnamiensis	2	0.975
rickettsia_bellii	2	0.955
desulfuromonas_acetoxidans	2	0.724
streptococcus_sanguinis	2	0.879
shewanella_massilia	2	0.895
flavobacterium_johnsoniae	2	1.032
streptomyces_thermoviolaceus	2	0.948
lactobacillus_sp	2	0.972
aquifex_pyrophilus	2	0.966
leuconostoc_mesenteroides	2	0.952
streptomyces_wedmorensis	2	0.943
serratia_proteamaculans	2	0.937
blautia_obeum	2	0.930
enterobacter_sp	2	0.828
clostridium_aminobutyricum	2	0.946
hydrogenovibrio_marinus	2	0.999
mycoplasma_mycoides	2	0.898
lyngbya_majuscula	2	0.827
psychromonas_ingrahamii	2	0.892
clostridium_cellulolyticum	2	0.977
streptomyces_thioluteus	2	0.869
micromonospora_griseorubida	2	0.909
sinorhizobium_fredii	2	0.831
weissella_paramesenteroides	2	0.964
streptoalloteichus_tenebrarius	2	0.951
clostridium_sporogenes	2	0.870
sporomusa_ovata	2	0.988
labrenzia_aggregata	2	1.011
enterococcus_casseliflavus	2	0.905
streptomyces_atroolivaceus	2	0.947
nocardioides_aromaticivorans	2	0.895
paraburkholderia_phytofirmans	2	0.968
gluconacetobacter_diazotrophicus	2	0.950
alcaligenes_xylosoxydans	2	1.012
geobacillus_thermoleovorans	2	0.914
eubacterium_siraeum	2	0.832
brevibacillus_brevis	2	0.908
mycoplasma_capricolum	2	0.880
acetobacterium_woodii	2	0.866
streptomyces_cinnamonensis	2	0.954
brachyspira_murdochii	2	0.921
prevotella_bryantii	2	0.874
serratia_fonticola	2	0.936
rhodoferax_ferrireducens	2	0.927
thermoanaerobacterium_thermosulfurigenes	2	0.964
clostridiales_bacterium	1	0.893
serratia	1	0.941
alteromonas_sp	1	0.881
streptomyces_aurantiacus	1	0.950
leifsonia_xyli	1	0.727
mycoplasma_mobile	1	0.830
herbaspirillum_seropedicae	1	0.946
leifsonia_aquatica	1	1.046
streptomyces_mobaraensis	1	0.906
paenibacillus_larvae	1	0.738
bacillus_pseudofirmus	1	0.742
moritella_profunda	1	0.786
marine_actinobacterium	1	0.957
streptomyces_rugosporus	1	0.960
bacillus_clarkii	1	0.959
slackia_exigua	1	0.999
salinispora_arenicola	1	0.920
pseudomonas_resinovorans	1	1.009
streptomyces_albogriseolus	1	0.994
symbiobacterium_toebii	1	0.978
novosphingobium_capsulatum	1	0.921
pandoraea_pnomenusa	1	0.996

mannheimia_haemolytica	1	0.926
flavobacterium_frigoris	1	0.975
kosmotoga_olearia	1	0.988
streptomyces_griseoruber	1	1.001
nitratireductor_pacificus	1	0.912
butyrivibrio_proteoclasticus	1	0.956
streptomyces_flavoviridis	1	0.791
amycolatopsis_balhimycina	1	0.838
alteromonas_macleodii	1	0.905
zoogloea_ramigera	1	0.971
clavibacter_michiganensis	1	0.982
ensifer_adhaerens	1	0.912
pseudonocardia_autotrophica	1	0.830
fervidobacterium_nodosum	1	1.028
exiguobacterium_oxidotolerans	1	0.877
xenorhabdus_bovienii	1	0.941
bacteroides_intestinalis	1	0.906
histophilus_somni	1	0.761
pseudomonas_alkylphenolica	1	0.936
psychrobacter_cryohalolentis	1	0.881
neptuniibacter_caesariensis	1	0.807
clostridium_hylemonae	1	0.790
cellulomonas_bogoriensis	1	1.028
gallionella_capsiferriformans	1	1.030
micromonospora_sp	1	0.881
hafnia_alvei	1	0.945
variovorax_paradoxus	1	1.018
kitasatospora_setae	1	0.780
mycoplasma_arginini	1	0.951
streptomyces_rimofaciens	1	0.910
bizionia_argentinensis	1	0.824
lyngbya_aestuarii	1	0.928
rhodococcus	1	0.841
bermanella_marisrubri	1	0.855
alicyclobacillus_sendaiensis	1	1.041
sinorhizobium_medicae	1	0.974
thermincola_potens	1	0.852
brevibacillus_agri	1	1.003
mitsuokella_multacida	1	0.868
streptomyces_maritimus	1	0.994
pseudomonas_dacunhae	1	1.002
mycobacterium_bovis	1	0.930
bacillus_caldotenax	1	0.773
rickettsia_africae	1	0.934
dictyoglomus_thermophilum	1	0.965
streptomyces_rimosus	1	0.825
thermoanaerobacter_ethanolicus	1	0.931
clostridium_propionicum	1	0.893
rubrobacter_xylanophilus	1	1.025
brevibacillus_laterosporus	1	0.609
xanthomonas_euvesicatoria	1	0.816
bartonella_sp	1	0.783
liberibacter_asiaticus	1	0.878
coryneform_bacterium	1	0.890
rhodospirillum_centenum	1	0.780
staphylococcus_cohnii	1	0.892
vitreoscilla_stercoraria	1	0.730
azospirillum_sp	1	0.977
mycobacterium_rhodesiae	1	0.901
bacillus_caldovelox	1	1.042
actinomycete_sp	1	0.886
lactobacillus_sanfranciscensis	1	0.904
providencia_alcalifaciens	1	0.898
streptomyces_pristinaespiralis	1	0.845
aeromonas_sobria	1	0.925
cellulomonas_gilvus	1	1.001
pelobacter_carbinolicus	1	0.811
clostridium_subterminale	1	0.944
thermus_brockianus	1	0.995
nitrospira_defluvii	1	0.905
methylacidiphilum_fumariolicum	1	1.041
aquaspirillum_arcticum	1	0.943
azoarcus_evansii	1	0.830
nocardia_otitidiscaviarum	1	0.890
streptomyces_noursei	1	0.821
pantoea_ananas	1	0.937
colwellia_sp	1	0.940
salmonella_enteritidis	1	0.817
streptococcus_mitis	1	0.788
jeotgalibacillus_marinus	1	0.879
thioalkalivibrio_paradoxus	1	0.786
streptomyces_morookaense	1	0.996
xylanimonas_cellulosilytica	1	1.014
sphingopyxis_alaskensis	1	0.851
paeniclostridium_sordellii	1	0.775
leptospirillum_rubarum	1	0.792
faecalibacterium_prausnitzii	1	0.959
polynucleobacter_necessarius	1	0.985
mycobacterium_goodii	1	0.966
vibrionales_bacterium	1	0.981
delftia_sp	1	1.021
pantoea_agglomerans	1	0.961
persephonella_marina	1	0.848
bartonella_clarridgeiae	1	0.871
pseudomonas_pseudoalcaligenes	1	0.967
acidaminococcus_sp	1	0.927
pseudarthrobacter_chlorophenolicus	1	0.942
salinispora_tropica	1	0.946
meiothermus_ruber	1	1.028
planomicrobium_okeanokoites	1	0.867
sphingopyxis_macrogoltabida	1	0.788
variovorax_sp	1	1.009
sanguibacter_keddieii	1	0.914

citrobacter_koseri	1	0.828
kuenenia_stuttgartiensis	1	0.815
shimwellia_blattae	1	0.853
bartonella_birtlesii	1	0.827
fluoribacter_gormanii	1	0.979
clostridium_hiranonis	1	0.794
hydrogenophilus_thermoluteolus	1	0.764
microbulbifer_thermotolerans	1	0.923
streptomyces_violaceoruber	1	0.776
streptomyces_rubiginosus	1	1.030
geobacillus_thermoglucosidasius	1	0.903
tannerella_forsythia	1	0.909
shigella_sonnei	1	0.766
clostridium_acidurici	1	0.802
francisella_novicida	1	0.934
rhodococcus_ruber	1	1.024
lactobacillus_gasseri	1	0.975
photobacterium_damselae	1	0.848
streptomyces_luridus	1	0.910
sphingosinicella_xenopeptidilytica	1	1.058
bifidobacterium_breve	1	0.959
actinomyces_odontolyticus	1	0.852
pseudoxanthomonas_mexicana	1	0.898
dinoroseobacter_shibae	1	0.851
thiobacillus_sp	1	1.054
actinomyces_urogenitalis	1	1.038
acholeplasma_laidlawii	1	1.016
bacillus_alcalophilus	1	0.946
thermus_scotoductus	1	1.040
citrobacter_sedlakii	1	0.852
thioalkalivibrio_nitratireducens	1	0.783
actinoalloteichus_cyanogriseus	1	0.871
prevotella_intermedia	1	0.946
cellulomonas_sp	1	0.960
rhodobacter_blasticus	1	0.962
clostridium_clariflavum	1	0.865
micromonospora_viridifaciens	1	0.941
streptomyces_chartreusis	1	0.895
micrococcus_antarcticus	1	0.958
kutzneria_sp	1	0.957
photobacterium_phosphoreum	1	0.853
streptomyces_nodosus	1	0.977
cycloclasticus_sp	1	0.882
roseovarius_sp	1	1.033
candidatus_cloacimonas	1	1.029
streptomyces_ansochromogenes	1	0.938
streptomyces_peucetius	1	0.883
fibrobacter_succinogenes	1	0.924
streptomyces_matensis	1	0.909
streptomyces_griseolus	1	0.873
pseudomonas_denitrificans	1	0.986
microbacterium	1	1.005
hathewaya_limosa	1	0.798
fervidobacterium_pennivorans	1	1.049
streptomyces_macromomyceticus	1	1.011
flavobacterium_frigidimaris	1	0.925
pseudothermotoga_lettingae	1	0.965
rhodococcus_hoagii	1	0.884
cupriavidus_taiwanensis	1	0.981
glutamicibacter_protophormiae	1	0.952
actinoplanes_missouriensis	1	0.968
idiomarina_baltica	1	0.943
actinobacillus_suis	1	0.795
thermosynechococcus_vulcanus	1	0.643
chlorobium_limicola	1	0.922
listeria_ivanovii	1	0.867
acidovorax_citrulli	1	0.957
thermobifida_alba	1	0.945
citrobacter_rodentium	1	0.838
chromobacterium_sp	1	0.977
delftia_acidovorans	1	0.996
sulfurospirillum_multivorans	1	0.905
pseudaminobacter_salicylatoxidans	1	0.882
nocardiopsis_alba	1	1.044
pseudomonas_chlororaphis	1	0.851
pelagibacter_ubique	1	0.944
ectothiorhodospira_shaposhnikovii	1	0.892
streptomyces_platensis	1	0.968
pseudomonas_mevalonii	1	0.972
mycoplana_ramosa	1	1.052
alteromonas_fortis	1	1.020
staphylococcus_staphylolyticus	1	0.926
streptomyces_seoulensis	1	0.857
pseudomonas_cichorii	1	1.002
streptomyces_roseochromogenus	1	0.963
staphylococcus_warneri	1	0.825
klebsiella_sp	1	0.945
streptomyces_griseocarneus	1	0.863
streptomyces_cacaoi	1	0.906
laribacter_hongkongensis	1	0.822
bacillus_velezensis	1	0.878
achromobacter_lyticus	1	0.981
pseudomonas	1	0.927
bacillus_firmus	1	0.914
streptomyces_bottropensis	1	0.928
streptomyces_melanosporofaciens	1	0.848
azorhizobium_caulinodans	1	0.952
sphingobium_chlorophenolicum	1	0.945
streptomyces_achromogenes	1	0.932
pelagibaca_bermudensis	1	1.014
paenarthrobacter_nitroguajacolicus	1	0.862
mycoplasma	1	0.871
azospira_oryzae	1	0.897

desulfarculus_baarsii	1	0.801
mesorhizobium_sp	1	0.979
brevibacterium_fuscum	1	0.886
haemophilus_aegyptius	1	0.887
clostridium_scindens	1	0.938
bacillus_akibai	1	0.900
thermopolyspora_flexuosa	1	0.909
moraxella_bovis	1	0.885
streptomyces_albidoflavus	1	0.895
sulfurihydrogenibium_sp	1	0.915
herpetosiphon_aurantiacus	1	0.887
pseudovibrio_sp	1	0.940
pantoea_stewartii	1	0.899
leptolyngbya_boryana	1	0.864
bradyrhizobium_elkanii	1	0.936
ochrobactrum_sp	1	0.966
pseudomonas_amyloderamosa	1	1.072
edta-degrading_bacterium	1	0.980
vibrio_splendidus	1	0.866
clostridium_papyrosolvens	1	0.975
sphingobacterium_multivorum	1	1.005
methylophaga_aminisulfidivorans	1	0.963
weissella_viridescens	1	0.893
clostridium_tetanomorphum	1	0.949
oceanobacter_kriegii	1	0.839
uncultured_prochloron	1	0.494
pseudomonas_reinekei	1	0.772
escherichia_fergusonii	1	0.912
streptomyces_griseoflavus	1	0.986
haemophilus_haemolyticus	1	0.894
leptospira_biflexa	1	0.865
acidimicrobium_ferrooxidans	1	0.857
cellulosimicrobium_cellulans	1	0.981
streptomyces_fimbriatus	1	0.844
methylobacterium_sp	1	0.870
chryseobacterium_indologenes	1	0.906
paucimonas_lemoignei	1	0.996
weissella_confusa	1	0.985
desulfococcus_multivorans	1	0.938
cyanothece_sp	1	0.825
streptomyces_bikiniensis	1	0.989
acidobacterium_capsulatum	1	0.987
thermoanaerobacter_brockii	1	1.016
borrelia_turicatae	1	0.785
erythrobacter_litoralis	1	0.733
neisseria_polysaccharea	1	0.999
nitrobacter_winogradskyi	1	0.718
kineococcus_radiotolerans	1	0.888
streptomyces_lydicus	1	0.933
streptococcus_anginosus	1	0.925
prochloron_sp	1	0.880
brachybacterium_faecium	1	0.899
kocuria_varians	1	0.835
jannaschia	1	0.933
wolbachia_pipientis	1	0.785
lactobacillus_helveticus	1	0.825
lactobacillus_pentosus	1	1.021
ammonifex_degensii	1	1.006
burkholderia_gladioli	1	1.023
uncultured_thermotogales	1	0.814
pseudoflavonifractor_capillosus	1	0.903
chryseobacterium_proteolyticum	1	0.877
anaerobiospirillum_succiniciproducens	1	0.995
prosthecobacter_dejongeii	1	0.987
xanthomonas_sp	1	0.913
plesiomonas_shigelloides	1	0.857
anoxybacillus_sp	1	0.928
helicobacter_felis	1	0.923
pseudomonas_amygdali	1	0.883
nitratiruptor_sp	1	0.972
chloroflexus_aggregans	1	0.957
streptomyces_rubellomurinus	1	0.966
citrobacter_amalonaticus	1	1.022
cellvibrio_sp	1	1.005
rickettsia_conorii	1	0.728
staphylococcus_xylosus	1	0.928
streptomyces_natalensis	1	0.860
dickeya_paradisiaca	1	1.034
brevibacillus_centrosporus	1	0.821
methylobacterium_radiotolerans	1	0.865
streptomyces_niveus	1	0.915
vibrio_fluvialis	1	1.038
amycolatopsis_sp	1	0.995
clostridium_josui	1	0.911
uncultured_soil	1	0.916
nonlabens_ulvanivorans	1	0.878
erysipelothrix_rhusiopathiae	1	0.812
streptococcus_cristatus	1	0.965
fulvimarina_pelagi	1	0.918
aneurinibacillus_sp	1	0.935
aeromonas_jandaei	1	1.002
pelobacter_acetylenicus	1	0.974
streptomyces_vinaceus	1	0.888
thermobaculum_terrenum	1	0.951
pseudoalteromonas_carrageenovora	1	0.875
bartonella_grahamii	1	0.831
microbacterium_arborescens	1	0.883
yersinia_kristensenii	1	0.889
thermobispora_bispora	1	0.997
leeuwenhoekiella_blandensis	1	0.886
streptomyces_ghanaensis	1	1.083
beutenbergia_cavernae	1	0.926
photorhabdus_asymbiotica	1	0.842

psychrobacter_sp	1	0.947
brevibacillus_parabrevis	1	0.988
mannheimia_succiniciproducens	1	0.852
cutibacterium_acnes	1	0.978
eubacterium_rectale	1	0.954
streptomyces_reticuli	1	1.021
streptomyces_sioyaensis	1	0.950
xanthomonas_albilineans	1	0.918
gulosibacter_molinativorax	1	1.011
streptoalloteichus_hindustanus	1	0.903
virgibacillus_salexigens	1	0.829
lactobacillus_rhamnosus	1	0.891
sulfurimonas_denitrificans	1	0.899
ketogulonicigenium_vulgare	1	0.865
streptococcus_uberis	1	0.878
granulibacter_bethesdensis	1	1.002
corynebacterium_callunae	1	0.922
brevibacterium_sterolicum	1	0.980
vibrio_campbellii	1	0.954
bacteroides_xylanisolvens	1	0.935
streptomyces_cyaneus	1	0.859
methylomicrobium_album	1	0.867
streptomyces_collinus	1	0.919
bacillus_thermoproteolyticus	1	0.942
paenarthrobacter_ureafaciens	1	0.934
nocardioides_sp	1	0.884
bordetella_petrii	1	1.025
slackia_heliotrinireducens	1	0.953
actinoplanes_sp	1	0.904
thermoanaerobacter_italicus	1	0.951
clostridium_sardiniense	1	0.826
listeria_grayi	1	0.929
thermobacillus_xylanilyticus	1	0.941
agromyces_sp	1	1.012
cellulomonas_uda	1	0.994
acinetobacter_johnsonii	1	0.941
mycobacterium_xenopi	1	0.970
mycobacterium_gastri	1	0.964
lactobacillus_leichmannii	1	0.955
atopobium_parvulum	1	0.952
frankia_sp	1	0.891
acinetobacter_radioresistens	1	0.867
serratia_sp	1	0.940
haemophilus_parasuis	1	0.925
pseudomonas_mesoacidophila	1	0.960
lactobacillus_hilgardii	1	0.974
cyanothece	1	0.570
nocardiopsis_sp	1	0.987
mycoplasma_hyorhinis	1	0.817
mycoplasma_arthritidis	1	0.758
microcystis_viridis	1	1.009
erwinia_rhapontici	1	0.962
megasphaera_elsdenii	1	0.930
sphingopyxis_sp	1	0.980
nocardioides_simplex	1	0.882
moritella_marina	1	0.909
marinomonas_mediterranea	1	0.830
komagataeibacter_hansenii	1	0.980
chromobacterium_viscosum	1	0.959
fischerella_thermalis	1	0.866
acidithiobacillus_thiooxidans	1	0.960
caulobacter_sp	1	0.994
thermus_sp	1	1.019
granulicella_tundricola	1	0.833
jeotgalicoccus_sp	1	0.896
hoeflea_phototrophica	1	0.993
streptomyces_resistomycificus	1	0.894
uncultured_murine	1	0.925
micromonospora_chalcea	1	0.926
marinomonas_sp	1	0.802
gluconobacter_frateurii	1	1.018
actinomadura_sp	1	0.921
ruegeria_lacuscaerulensis	1	0.923

Table S12. List of archaea organisms in the dataset $\mathcal{D}$, with their number of proteins and average $\Delta\Delta G$ upon all possible point mutations

Organism	Number of proteins	$\langle\Delta\Delta G\rangle$ (kcal/mol)
pyrococcus_horikoshii	189	0.950
archaeoglobus_fulgidus	138	0.932
methanocaldococcus_jannaschii	130	0.930
pyrococcus_furiosus	118	0.946
sulfolobus_solfataricus	106	0.933
sulfolobus_tokodaii	60	0.943
thermoplasma_acidophilum	56	0.923
aeropyrum_pernix	44	0.977
methanothermobacter_thermautotrophicus	44	0.928
haloarcula_marismortui	37	0.787
thermococcus_kodakarensis	31	0.950
pyrococcus_abyssi	29	0.927
methanosarcina_mazei	29	0.900
pyrobaculum_aerophilum	24	0.985
methanopyrus_kandleri	16	0.978
sulfolobus_acidocaldarius	13	0.962
thermoplasma_volcanium	12	0.925
methanosarcina_barkeri	12	0.941
methanococcus_maripaludis	9	0.906
halobacterium_salinarum	9	0.832
thermococcus_litoralis	8	0.961

methanosarcina_acetivorans	8	0.915
thermococcus_onnurineus	8	0.922
haloferax_volcanii	6	0.920
pyrobaculum_calidifontis	6	0.950
methanothermobacter_marburgensis	5	0.970
thermococcus_sp	4	0.910
uncultured_archaeon	4	0.935
sulfolobus_islandicus	4	0.891
thermoproteus_tenax	4	1.059
pyrococcus_woesei	3	1.052
nanoarchaeum_equitans	3	0.912
methanothermus_fervidus	3	0.892
methanosarcina_thermophila	3	0.966
picrophilus_torridus	3	0.935
sulfolobus_shibatae	3	0.985
pyrococcus_sp	2	0.914
natronomonas_pharaonis	2	0.764
acidilobus_saccharovorans	2	1.020
pyrobaculum_neutrophilum	2	1.076
staphylothermus_marinus	2	0.942
haloferax_mediterranei	2	0.926
methanococcus_voltae	2	0.945
acidianus_ambivalens	2	0.994
halomicrobium_mukohataei	2	0.836
thermococcus_profundus	2	1.013
methanoculleus_marisnigri	1	0.920
thermofilum_pendens	1	0.899
acidianus_sp	1	0.968
ignicoccus_hospitalis	1	0.996
acidianus_hospitalis	1	0.913
thermococcus_sibiricus	1	1.067
ferroplasma_acidiphilum	1	0.978
korarchaeum_cryptofilum	1	0.762
thaumarchaeota_archaeon	1	0.921
methanospirillum_hungatei	1	0.927
vulcanisaeta_moutnovskia	1	1.035
thermococcus_thioreducens	1	1.187
methanoculleus_thermophilus	1	0.994
thermosphaera_aggregans	1	0.947
metallosphaera_sedula	1	1.040
methanosaeta_thermophila	1	0.894
pyrobaculum_islandicum	1	1.030
thermoproteus	1	0.907
candidatus_micrarchaeum	1	0.873
pyrobaculum_ferrireducens	1	0.965
thermococcus_celer	1	0.996
methanocorpusculum_labreanum	1	0.845

Table S13. List of virus organisms in the dataset $\mathcal{D}$, with their number of proteins and average $\Delta\Delta G$ upon all possible point mutations

Organism	Number of proteins	$\langle\Delta\Delta G\rangle$ (kcal/mol)
mus_musculus_polyomavirus	682	0.866
thermus_thermophilus_bacteriophage	419	0.953
enterobacteria_phage	72	0.863
influenza_a	67	0.904
escherichia_phage	36	0.864
pyrococcus_abyssi	29	0.927
clostridium_botulinum	26	0.851
human_immunodeficiency	26	0.860
vaccinia_virus	26	0.814
bombyx_mori	22	0.865
bacillus_phage	20	0.869
human_herpesvirus	17	0.862
human_adenovirus	16	0.898
hepatitis_c	16	0.920
pseudomonas_phage	13	0.890
human_sars	12	0.871
dengue_virus	12	0.835
salmonella_phage	11	0.924
human_papillomavirus	9	0.835
murid_herpesvirus	9	0.849
acanthamoeba_polyphaga	9	0.840
epstein-barr_virus	9	0.875
paramecium_bursaria	8	0.902
sulfolobus_turreted	8	0.967
influenza_b	7	0.911
lactococcus_phage	7	0.848
human_cytomegalovirus	6	0.879
infectious_bronchitis	6	0.894
murine_coronavirus	6	0.824
autographa_californica	6	0.827
west_nile	6	0.850
pyrobaculum_spherical	5	0.862
human_coronavirus	5	0.811
ectromelia_virus	5	0.882
acidianus_filamentous	5	0.777
staphylococcus_phage	5	0.835
moloney_murine	5	0.821
human_respiratory	5	0.800
rotavirus_a	4	0.848
rous_sarcoma	4	0.837
rabbit_hemorrhagic	4	0.904
nipah_virus	4	0.826
cowpox_virus	4	0.909
zaire_ebolavirus	4	0.783
streptococcus_phage	4	0.899
human_rhinovirus	4	0.907
simian_immunodeficiency	4	0.864

japanese_encephalitis	4	0.847
shigella_phage	4	0.899
sulfolobus_islandicus	4	0.891
lymphocytic_choriomeningitis	3	0.794
white_spot	3	0.831
rabies_virus	3	0.784
simian_rotavirus	3	0.753
unidentified_phage	3	0.986
murine_norovirus	3	0.862
norwalk_virus	3	0.926
chikungunya_virus	3	0.846
crimean-congo_hemorrhagic	3	0.813
human_betacoronavirus	3	0.839
mason-pfizer_monkey	3	0.821
vesicular_stomatitis	3	0.877
parainfluenza_virus	3	0.870
equine_arteritis	3	0.952
ustilago_maydis	3	0.929
simian_virus	3	0.881
enterovirus_a71	3	0.916
human_polyomavirus	3	0.958
venezuelan_equine	3	0.920
avian_infectious	3	0.846
adeno-associated_virus	3	0.878
acidianus_two-tailed	2	0.884
human_calicivirus	2	0.921
poliovirus_type	2	0.903
avian_orthoreovirus	2	0.851
yaba-like_disease	2	0.844
jc_polyomavirus	2	0.886
mycobacterium_phage	2	0.893
clostridium_virus	2	0.898
machupo_virus	2	0.853
rift_valley	2	0.886
clostridium_phage	2	0.950
african_swine	2	0.675
bovine_coronavirus	2	0.900
feline_immunodeficiency	2	0.797
bovine_papillomavirus	2	0.814
listeria_phage	2	0.895
borna_disease	2	0.888
reovirus_sp	2	0.979
coxsackievirus_a16	2	0.918
bunyavirus_la	2	0.822
murray_valley	2	0.834
reston_ebolavirus	2	0.855
norwalk-like_virus	2	0.920
foot-and-mouth_disease	2	0.901
hepatitis_e	2	1.006
human_rotavirus	2	0.888
pseudoalteromonas_phage	2	0.860
coxsackievirus_b3	2	0.910
porcine_transmissible	2	0.823
rubella_virus	2	0.951
sudan_ebolavirus	2	0.831
lassa_mammarenavirus	2	0.813
infectious_pancreatic	2	0.888
porcine_reproductive	2	0.935
megavirus_chiliensis	2	0.872
yellow_fever	2	0.849
thogoto_virus	2	0.806
sindbis_virus	1	0.952
bluetongue_virus	1	0.876
human_astrovirus-8	1	0.897
suid_alphaherpesvirus	1	0.897
enterovirus_d68	1	0.928
myxoma_virus	1	0.825
porcine_torovirus	1	0.912
kunjin_virus	1	0.823
bk_polyomavirus	1	0.885
mimivirus	1	0.935
blotched_snakehead	1	0.872
aura_virus	1	0.927
norovirus_hu/gii-4/kumamoto5/2006/jp	1	0.906
mammalian_rubulavirus	1	0.991
norovirus_hu/gii4/sydney/nsw0514/2012/au	1	0.860
granada_virus	1	0.837
human_spumaretrovirus	1	0.920
wiseana_signata	1	0.772
hepatitis_delta	1	0.792
ostreococcus_tauri	1	0.800
macacine_betaherpesvirus	1	0.702
snake_adenovirus	1	0.793
norovirus_cat/giv2/cu081210e/usa/2010	1	0.921
xmrv	1	0.735
haemophilus_phage	1	0.870
bordetella_phage	1	0.983
lettuce_necrotic	1	0.670
bovine_viral	1	0.746
african_horse	1	0.954
middle_east	1	0.796
singapore_grouper	1	0.755
grouper_iridovirus	1	1.001
tick-borne_encephalitis	1	0.952
merkel_cell	1	0.968
enterovirus_b	1	0.892
reovirus_type	1	0.921
orf_virus	1	0.763
semliki_forest	1	0.905
hepatitis_b	1	0.805
bovine_immunodeficiency	1	0.773
mengo_encephalomyocarditis	1	0.886
feline_coronavirus	1	0.811

rotavirus_sp	1	0.924
human_parainfluenza	1	0.959
dhori_virus	1	0.808
unidentified_influenza	1	0.922
friend_murine	1	1.011
satellite_tobacco	1	0.927
escherichia_virus	1	1.052
avian_sarcoma	1	0.884
fowl_adenovirus	1	0.969
nodamura_virus	1	0.776
hendra_virus	1	0.663
vibrio_phage	1	0.933
human_t-lymphotropic	1	0.940
porcine_circovirus	1	0.833
infectious_hypodermal	1	0.932
cyprinid_herpesvirus	1	0.699
tomato_bushy	1	0.842
avian_avulavirus	1	0.945
southampton_virus	1	0.876
pestivirus_strain	1	0.859
human_metapneumovirus	1	0.849
feline_leukemia	1	0.881
newcastle_disease	1	0.910
aeromonas_virus	1	0.737
modoc_virus	1	0.891
tellina_virus	1	0.902
rice_hoja	1	0.806
simian_adenovirus	1	0.881
measles_morbillivirus	1	0.765
norovirus_hu/gii4/farmington	1	0.919
carnation_italian	1	0.779
human_mastadenovirus	1	0.910
dg-75_murine	1	0.953
yellowtail_ascites	1	0.912
lake_victoria	1	0.862
operophtera_brumata	1	0.796
norovirus_hu/gi7/tch-060/usa/2003	1	0.922
tomato_mosaic	1	0.825
breda_virus	1	0.934
hepatovirus_a	1	0.839
satellite_panicum	1	1.005
sulfolobus_virus	1	0.881
silicibacter_phage	1	0.883
molluscum_contagiosum	1	0.831
mopeia_lassa	1	0.819
staphylococcus_virus	1	0.822
avian_myeloblastosis	1	0.696
akv_murine	1	0.848
classical_swine	1	0.875
sheep_pulmonary	1	0.782
tobacco_mosaic	1	0.866
murine_minute	1	0.792
norovirus_hu/gii4/2004/nl	1	0.868
transmissible_gastroenteritis	1	0.931
sesbania_mosaic	1	0.906
phlebovirus_js2010-018	1	0.888
schmallenberg_virus	1	0.750
turnip_yellow	1	0.923
kokobera_virus	1	0.840
sulfolobus_spindle-shape	1	0.745
synechococcus_phage	1	0.786
turkey_astrovirus	1	0.877
lactococcus_virus	1	0.826
maedi_visna	1	1.015
tobacco_vein	1	0.866
sin_nombre	1	0.575
bacteriophage_h30	1	1.009
mumps_rubulavirus	1	0.809
ebola_virus	1	0.837
junin_mammarenavirus	1	0.791
lassa_virus	1	0.821
human_astrovirus-1	1	1.023
prochlorococcus_phage	1	0.752
turnip_mosaic	1	0.761
norovirus	1	0.891
thermus_phage	1	0.808
equid_alphaherpesvirus	1	0.847
enterobacterio_phage	1	0.793
b-lymphotropic_polyomavirus	1	0.897
equine_infectious	1	0.887
acinetobacter_bacteriophage	1	0.843
canine_adenovirus	1	0.972
sapporo_virus	1	0.899
monkeypox_virus	1	0.874
tobacco_etch	1	0.949
